# Exploring Biological Activity of 4-Oxo-4*H*-furo[2,3-*h*]chromene Derivatives as Potential Multi-Target-Directed Ligands Inhibiting Cholinesterases, β-Secretase, Cyclooxygenase-2, and Lipoxygenase-5/15

**DOI:** 10.3390/biom9110736

**Published:** 2019-11-13

**Authors:** Malose J. Mphahlele, Emmanuel N. Agbo, Samantha Gildenhuys, Itumeleng B. Setshedi

**Affiliations:** 1 Department of Chemistry, College of Science, Engineering and Technology, University of South Africa, Private Bag X06, Florida 1710, South Africa; piruesbest@yahoo.com; 2 Department of Life & Consumer Sciences, College of Agriculture and Environmental Sciences, University of South Africa, Private Bag X06, Florida 1710, South Africa; setshib@unisa.ac.za

**Keywords:** furochromones, cholinesterases, β-secretase, cyclooxygenase-2, lipoxygenases-5/15, anti-oxidant activity, cytotoxicity, molecular docking

## Abstract

A series of 5-oxo-5*H*-furo[3,2-*g*]chromene-6-carbaldehydes and their hydrazone derivatives were evaluated as potential multi-target-directed ligands in vitro against cholinesterases, β-secretase, cyclooxygenase-2, and lipoxygenase-15 (LOX-15), as well as for free radical-scavenging activities. The most active compounds against LOX-15 were also evaluated for activity against the human lipoxygenase-5 (LOX-5). Kinetic studies against AChE, BChE, and β-secretase (BACE-1) were performed on 2-(3-fluorophenyl)- (**3b**) and 2-(4-chlorophenyl)-6-[(4-trifluoromethylphenyl)hydrazonomethyl]furo[3,2-*h*]chromen-5-one (**3e**) complemented with molecular docking (in silico) to determine plausible protein-ligand interactions on a molecular level. The docking studies revealed hydrogen and/or halogen bonding interactions between the strong electron-withdrawing fluorine atoms of the trifluoromethyl group with several residues of the enzyme targets, which are probably responsible for the observed increased biological activity of these hydrazone derivatives. The two compounds were found to moderately inhibit COX-2 and lipoxygenases (LOX-5 and LOX-15). Compounds **3b** and **3e** were also evaluated for cytotoxicity against the breast cancer MCF-7 cell line and Hek293-T cells.

## 1. Introduction

Alzheimer’s disease (AD) is a neurodegenerative disorder that affects elderly people worldwide, and is characterized by various symptoms, including memory loss, emotional behavior, and impaired decision-making [1]. AD is pathologically characterized by amyloid beta (Aβ) peptide, phosphorylated tau protein, oxidative stress, and cholinergic system. A major hallmark of AD is the formation and accumulation of fibrillar β-amyloid (Aβ) peptides in the brain, which starts many years before the first symptoms occur [2]. Over-the-counter anti-Alzheimer’s drugs such as tacrine, donepezil, rivastigmine, and galantamine have been found to temporally improve cognitive functions in AD patients by inhibiting the breakdown of the neurotransmitter acetylcholine (ACh) [3]. These drugs act by inhibiting enzymes involved in ACh termination of action in neuron synapsis, namely, acetylcholinesterase (AChE) and butyrylcholinesterase (BChE) [4]. AChE is selectively responsible for hydrolyzing the neurotransmitter acetylcholine (ACh) in the early stages of AD, while BChE acts as the major ACh degrading enzyme in AD progression [5]. As the disease progresses, the level of AChE in the brain of AD patients has been found to decrease extensively, while BChE activity is maintained at the normal or even at a higher level [6]. Selective inhibition of BChE activity could be advantageous for the treatment of advanced AD, because it may circumvent classical cholinergic toxicity like nausea and vomiting, which are common side effects of AChE inhibition; however, it can also lead to adverse peripheral side effects [7]. Thus, it is necessary to design cholinesterase (ChE) inhibitors that simultaneously block both the catalytic and peripheral anionic sites of AChE and the catalytic activity of BChE; this would be of dual benefit by increasing cholinergic transmission and potentially slowing down the formation of the neurotoxic extracellular plaques [8]. Chronic inflammation is a common phenomenon present in the background of multiple human disorders such as AD, diabetes, and cancer [9], and has come to be recognized as an important pathophysiological feature and early hallmark of AD [10,11]. AD arises from a complex interplay of different pathways, and the inhibition of cholinesterase enzymes alone fails to stop the progression of this disease [12]. In the search for novel and effective treatment strategies, multi-target-directed ligand design strategies (MTDLs) have been recognized as being more effective for the treatment of AD than one-target one-drug concepts and drug-combination therapies [13,14]. Targeting cholinesterase and inflammatory pathways simultaneously, for example, could help diminish the initiation and progression of AD [8]. Enzymes play essential roles in life processes and represent attractive targets for drug therapy because they are highly responsive to inhibition by small molecular weight, drug-like molecules. Moreover, about 47% of all current drugs in the market have been found to inhibit enzyme targets [15]. Acetylcholinesterase (AChE) and butyrylcholinesterase (BChE) are the key targets employed in the early discovery and development of drug candidates for the treatment of AD. Cyclooxygenases and lipoxygenases, on the other hand, are important enzymes for the discovery of mechanism-based drugs for the treatment of inflammatory disorders [16,17,18].

**Figure 1:**
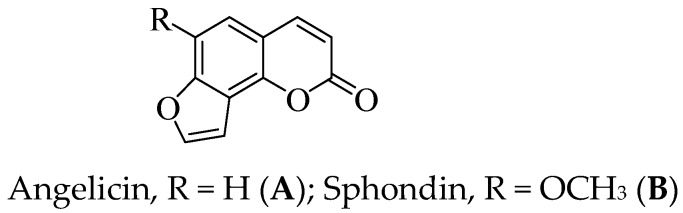
Structures of angelicin (**A**) and sphondin (**B**).

Naturally-occurring and chemically-synthesized benzofuran- and chromone-based compounds have found pharmacological applications in the field of neurodegenerative and inflammatory diseases as well as cancer [19]. Benzofuran- and chromone-based compounds have previously been evaluated for their anti-Alzheimer’s properties against cholinesterase enzymes (AChE and BCHE), β-secretase (BACE-1), and monoamine oxidae B (MAO-B) [20,21,22]. The anti-inflammatory potential of these classes of heterocyclic compounds is due to their targeting of cyclooxygenase and lipoxygenase enzymes [21,23]. Although cyclooxygenases and lipoxygenases are primarily responsible for inflammation and pain, studies have shown that COX-2 is related to cancers, mainly breast cancer [24]. Benzofurans and chromones also target cancer by directly or indirectly inhibiting the process of tubulin polymerization, mitosis, and DNA replication by inhibiting various enzymes like protein kinases, caspases, and heat shock proteins [23]. Naturally-occurring furocoumarins, angelicin **A** [25], and sphondin **B**, shown in Figure 1, exhibit anti-inflammatory activity [26]. We decided to merge the two pharmacophores to construct angular furochromone carbaldehyde, which is analogous of angelicin with potential anti-Alzheimer’s and anti-inflammatory properties. The inhibitory effects of the prepared furochromone carbaldehydes and their hydrazone derivatives were evaluated in vitro against the following enzyme targets involved in AD: AChE, BChE, and β-secretase. Kinetic and molecular docking studies were undertaken on the most active compounds to try to determine plausible protein-ligand interactions on a molecular level. Since chronic inflammation is implicated in the early stages of AD, and due to the complexity and multifactorial origins of the disease, the scope of the present investigation was extended to include cyclooxygenase-2 (COX-2) and lipoxygenases-5/15 (LOX-5 and LOX-15). Most lipoxygenase inhibitors exhibit inhibitory effects via antioxidant mechanisms by either scavenging radicals or preventing the oxidation of iron at the active site to inhibit enzyme activity [27]. Consequently, the aforementioned compounds were evaluated for their antioxidant potential.

## 2. Results and Discussion

### 2.1. Chemistry

**Scheme 1:**
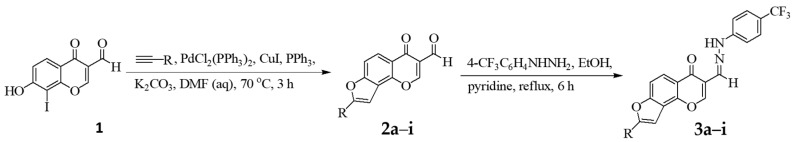
Synthesis of angular furochromones **2a**–**i** and hydrazone derivatives **3a**–**i**.

The synthetic pathways and substitution patterns of the 8-carbo–substituted 4-oxo-4*H*-furo[2,3-*h*]chromene-3-carbaldehydes and their hydrazone derivatives are represented in Scheme 1 and Table 1, respectively. The substrate 8-hydroxy-7-iodo-4-oxo-4*H*-chromene-3-carbaldehyde (**1**) was prepared in 73% yield by the treatment of 2,4-dihydroxy-3-iodoacetophenone with a mixture of phosphoryl chloride and dimethyl formamide (Vilsmeier-Haack reagent); the compound was then heated under reflux for 2 h. Attempted tandem Sonogashira cross-coupling of this *o*-iodophenol derivative with phenylacetylene in the presence of dichlorobis(triphenylphosphine)palladium(II) and a copper(I) iodide catalyst mixture and potassium carbonate as a base in 4:1 dimethyl formamide-water at room temperature for 18 h resulted in the recovery of the starting material. After several attempts varying the base and solvents without success, we resorted to the use of triphenyl phosphine as a ligand in the presence of a PdCl_2_(PPh_3_)_2_–CuI catalyst mixture. Triphenyl phosphine has previously been found to accelerate the oxidative addition (rate limiting step) and reductive elimination steps in palladium-catalyzed cross-coupling reactions [28,29]. Compound **1** was reacted with various terminal acetylenes in the presence of a PdCl_2_(PPh_3_)_2_-CuI catalyst mixture, triphenyl phosphine, and K_2_CO_3_ in aqueous DMF at 70 °C. Thin layer chromatography monitoring revealed that the reaction was completed within 3 h. Aqueous work-up and purification by column chromatography on silica gel afforded the corresponding furochromone-3-carbaldehydes **2a**–**i**. The latter are the result of one-pot tandem Sonogashira cross-coupling and Cacchi-type annulation. The ^1^H-NMR spectra of derivatives **2a**–**h** revealed the presence of an increased number of proton signals in the aromatic region and the absence of the signal for the hydroxyl group. 2-Cyclohexenyl–substituted furochromone **2i**, on the other hand, showed multiplets in the aliphatic region, as well as a triplet around δ 6.64 ppm for =CH. The ^13^C-NMR spectra of compounds **2a**–**i** lacked the two singlets in the region δ 80–100 ppm which are typical of the acetylene moiety, thereby ruling out the possibility of the alkynylated intermediates. The accurately-calculated *m*/*z* values of compounds **2a**–**i**, on the other hand, were found to be consistent with the assigned molecular structures of these angular furochromone derivatives. The presence of a trifluoromethyl group in a molecule has been found to increase biological activity due to its metabolic stability and enhanced lipid solubility, and therefore, membrane permeability [30]. Moreover, the strong electron-withdrawing effect of the halogen atoms helps in forming halogen and/or hydrogen bonds that help to increase the number of interactions of drug molecules with their protein targets, thereby enhancing biological activity [31]. With these considerations in mind, we decided to link a 4-(trifluoromethyl)phenyl group with a furochromone scaffold through a potential hydrogen bond-donating diazamethylidene (-CH=N-NH-) bridge/spacer. The spacer may reduce the hydrophobicity of the entire molecule and increase the solubility to generate compounds with the potential to interact with the peripheral anionic site (PAS) and catalytic site (CAS) of AChE. Compounds **2a**–**i** were subjected to nucleophilic addition with 4-(trifluoromethyl)phenylhydrazine in the presence of pyridine in ethanol under reflux for 6 h. The corresponding hydrazone derivatives **3a**–**i** were isolated by aqueous work-up and purification by silica gel column chromatography. The hydrazone nature of these compounds was corroborated using a combination of NMR (^1^H-, ^13^C-, and ^19^F-), infrared, and mass spectrometric techniques. Their ^1^H- and ^13^C-NMR spectra revealed the presence of an increased number of signals in the aromatic region, which distinguishes their structures from those of the corresponding substrates.

Compounds **2a**–**i** and **3a**–**i** were evaluated for inhibitory effect in vitro against cholinesterases (AChE and BChE), β-secretase, COX-2, and LOX-15, as well as for antioxidant potential, as described below.

### 2.2. Biological Activity Studies

The structure activity relationship (SAR) of these angular furochromone derivatives was studied with respect to the substitution pattern on the 2-position of the furan ring and also the effect of transforming the carbaldehyde into hydrazone functionality.

#### 2.2.1. AChE and BChE Inhibitory Activities of Compounds **2a**–**i** and **3a**–**i**

The 3-carbaldehyde-substituted derivatives **2a**–**i** were found to be generally less active against AChE when compared to the reference standards donepezil (IC_50_ = 0.02 μM) and galantamine (IC_50_ = 0.01 μM), with IC_50_ values in the range 15.2–34.2 μM (Table 2). Compound **2b** and **2i** substituted with a 3-fluorophenyl group or cyclohexenyl moiety on the furan moiety were found to be moderately inhibiting and to exhibit comparable activity against AChE with IC_50_ values of 15.2 μM and 15.3 μM, respectively. Compound **2b** also exhibited a significant inhibitory effect against BChE activity, with an IC_50_ value of 9.2 μM (Table 2). The presence of a 3-chlorophenyl group on the furan ring of **2d** also resulted in moderate activity against AChE (IC_50_ = 19.2 μM) and BChE (IC_50_ = 13.2 μM). The presence of a halogen atom on the para-position of the phenyl substituent on the furan scaffold of **2c** and **3e** resulted in a reduced inhibitory effect against both enzymes. Likewise, the electron-donating group (by inductive effect: -CH_3_ or resonance effect: -OCH_3_) on the para- (**2e** and **2h**) or both meta- (**2h**) positions of the phenyl group on the furan scaffold resulted in reduced inhibitory effect in this series. Within the series **3a**–**i**, only the 3-fluorophenyl **3b** and 4-chlorophenyl–substituted 4-(trifluoromethyl)phenylhydrazone derivative **3e** were found to a exhibit dual inhibitory effect against AChE (IC_50_ values 10.4 μM and 5.4 μM, respectively) and BChE (IC_50_ = 7.7 μM and 9.9 μM, respectively) activities. Their isomeric 4-fluorophenyl– **3c** and 3-chlorophenyl–substituted hydrazone derivative **3d** exhibited moderate activity against AChE (IC_50_ = 18.1 μM and 24.3 μM, respectively) and a reduced inhibitory effect against BChE (IC_50_ = 15.6 μM and 30.1 μM, respectively). The presence of the strongly electron-donating methoxy group on the para-position of the phenyl ring of **3f** or at both meta-positions of the phenyl ring of **3g** resulted in reduced activity against both enzymes. However, a less lipophilic 4-tolyl group on the furan ring of **3h** imparted significant activity against AChE (IC_50_ = 9.10 μM) and moderate activity against BChE (IC_50_ = 14.24 μM). A moderate inhibitory effect against AChE and BChE was observed for the cyclohexenyl–substituted hydrazone derivative **3i** with IC_50_ values of 13.30 μM and 17.9 μM, respectively. Since the activity of AChE is known to decline during the development of AD while BChE activity increases, compound **2b**, which is more active against BChE, may be more beneficial in alleviating the cognitive symptoms associated with moderate forms of AD. Compounds **3b** and **3e**, on the other hand, have the potential to block both the catalytic and peripheral anionic sites of AChE and the catalytic activity of BChE.

The overproduction of amyloid beta (Aβ) by β-secretase has been found to result in toxic fibrils which cause neurodegeneration which is characteristic of the AD [32]. Compounds **3b** and **3e**, with a dual inhibitory effect against both cholinesterase enzymes, were, in turn, evaluated for their inhibitory potential against β-secretase activity.

#### 2.2.2. β-Secretase Inhibitory Activities of Compounds **3b** and **3e**

Compounds **3b** and **3e** not only displayed better dual activity against cholinesterases, but also against β-secretase with lower IC_50_ values in both cases. Both compounds were found to exhibit significant inhibitory effects against β-secretase when compared to quercetin (IC_50_ = 12.9 µM); their IC_50_ values were 15.8 µM and 13.6 µM, respectively (Table 3).

Chromones as anti-inflammatory agents inhibit cyclooxygenase (COX) and lipoxygenase (LOX) production [33], which are important for the discovery of mechanism-based drugs for the treatment of inflammatory disorders. In an attempt to address the complexity and multifactorial origins of AD, we widened the spectrum of biological activity of the title compounds to include the inhibition of cyclooxygenase and lipoxygenase activities.

#### 2.2.3. In Vitro Inhibitory Assays against Cyclooxygenase-2 and Lipoxygenases

Both series of compounds were evaluated for their potential anti-inflammatory properties against COX-2 and LOX-15; the corresponding results expressed as means of IC_50_ values in µM from duplicate runs are summarized in Table 4 below. Although relatively less active against COX-2 compared to the anti-inflammatory drug celecoxib, used as a reference standard, the trend in the inhibitory effects of compounds **2a**–**i** seems to be influenced by the type of substituent and the pattern of substitution on the furan ring. The carbaldehyde derivatives substituted with an electron-donating group at the para position of the phenyl substituent exhibited significant inhibitory activity against COX-2 compared to derivatives substituted on the meta position of the ring with a halogen (**2b:** F or **2d:** Cl) or methoxy group (**2g**: 3,5-dimethoxy). The trend in the inhibitory effects of these compounds is as follows: **2f** (4-MeOC_6_H_4_-: 13.7 μM) > **2c** (4-FC_6_H_4_-: 15.8 μM) > **2e** (4-ClC_6_H_4_-: 18.6 μM) > **2h** (4-CH_3_C_6_H_4_-: 19.8 μM) > **2a** (C_6_H_5_-: 25.6 μM). The cyclohex-1-en-1-yl derivative **2i** was found to be the most active within this series, with an IC_50_ value of 12.1 μM. Amongst the hydrazone derivatives, only compounds **3e**, **3f*,*** and **3i** substituted on C-2 of the furan ring with a 4-chlorophenyl-, 4-methoxyphenyl-, or cyclohex-1-en-1-yl group exhibited a significant inhibitory effect against COX-2, with IC_50_ values of 10.4, 14.7, and 13.6 μM, respectively. Compound **3e**, with a dual inhibitory effect against cholinesterases and β-secretase, was found to be the most active against COX-2 within this series. The potential dual cholinesterase and β-secretase inhibitor **3b**, on the other hand, exhibited reduced inhibitory effects against this enzyme. The results for compounds **2a**–**i** against the soybean lipoxygenases-15 (LOX-15) show that activity against this enzyme is favored by electron-donating substituent/s on the 8-phenyl substituent. Compound **2f** substituted with a strong electron delocalizing 4-methoxyphenyl group on the furan ring was found to be the most active against LOX-15, with an IC_50_ value of 8.2 μM. The 3,5-dimethoxyphenyl–substituted derivative **2g**, which exhibited reduced activity against COX-2, was found to exhibit significant inhibitory effect against LOX-15 (IC_50_ = 10.6 μM), though it was relatively less active than **2f**. This is presumably because the propensity of the methoxy group for electron-pair delocalization is more pronounced when at the ortho or para position of the phenyl ring. The electron-donating inductive effect of the methyl group at the para position of the phenyl ring, on the other hand, resulted in significant activity for the 4-tolyl–substituted derivative **2h** against LOX-15 (IC_50_ = 9.2 μM). This compound exhibits moderate activity against COX-2 and a significant inhibitory effect against LOX-15. Compounds **3b** and **3e** were found to be moderately active against LOX-15, with IC_50_ values of 24.6 μM and 14.9 μM, respectively. Compound **3e**, which exhibited dual inhibition against cholinesterases (AChE and BChE) and β-secretase activities, was also found to exhibit dual activity against COX-2 and LOX-15. Within the series of hydrazone derivatives, the 4-methoxyphenyl-, 3,5-dimethoxyphenyl-, and 4-tolyl- substituted derivatives were found to be the most active against LOX-15; the trend in activity is as follows **3f** (IC_50_ = 6.1 μM), **3g** (IC_50_ = 9.4 μM), and **3h** (IC_50_ = 18.6 μM), respectively. This trend in activity presumably reflects the polarity or lipophilicity of the substituent on the phenyl ring. The cyclohexenyl-substituted hydrazone derivative **3i**, which is the most inhibiting against COX-2 within this series, was found to be less active or inactive against LOX-15. Although the results of this assay cannot be extrapolated to the inhibition of mammalian lipoxygenase, the inhibition of plant LOX activity by nonsteroidal anti-inflammatory agents has been found to be qualitatively similar to the inhibition they cause to the rat mast cell LOX [34]. Compounds **2f**–**h** and **3b**, **3e**–**g** with moderate or significant activity against LOX-15 were, in turn, screened for their inhibitory effects against the human LOX-5 using quercetin and zileuton as reference standards (Table 4). Zileuton has been approved by the Food and Drug Administration as a LOX-5 inhibitor for the treatment of bronchial asthma [35]. These carbaldehydes and hydrazone derivatives were found to be moderately inhibiting against LOX-5, with IC_50_ values in the range 17.3–34.1 μM. Compound **2f** was found to be less active against AChE and BChE; however, this compound exhibited a significant inhibitory effect against COX-2 (IC_50_ = 13.7 μM), LOX-5 (IC_50_ = 17.3 μM), and LOX-15 (IC_50_ = 8.2 μM). Similar behavior was observed for the 3,5-dimethoxyphenyl-substituted hydrazone derivative **3g** against COX-2 (IC_50_ = 17.6 μM) and lipoxygenases with IC_50_ values of 19.1 μM and 9.4 μM for LOX-5 and LOX-15, respectively. Compounds **2f** and **3g**, with significant activity against COX-2 and lipoxygenase-5/15, represent suitable scaffolds for the development of anti-inflammatory agents. Compound **3e** exhibited a significant inhibitory effect against lipoxygenase-5/15 compared to **3b**, though both represent potential dual inhibitors against cholinesterases and β-secretase.

Although the title compounds lack the hydroxyl group/s and are unsubstituted at the C-2 position, they contain the requisite radical scavenging 2,3-double bond in conjugation with a 4-oxo function in the pyrone ring for possible electron delocalization. Both series of compounds were evaluated for their antioxidant potential using the 2,2-diphenyl-1-picrylhydrazyl (DPPH) radical scavenging assay. The carbaldehyde precursors **2a**–**i** were found to exhibit stronger anti-oxidant effects than the corresponding hydrazone derivatives **3a**–**I** when compared to ascorbic acid (IC_50_ = 4.6 μM) with IC_50_ values 7.5–29.2 µM and 16.3–32.5 µM, respectively (Table 4). Compounds **2f**–**h**, which exhibit significant inhibitory effects against LOX-15 and moderate activity against LOX-5, as well as significant radical scavenging activity, could serve better as anti-inflammatory agents. The most active compounds against cholinesterase and β-secretase, namely **3b** and **3e**, were found to exhibit moderate antioxidant effects in the DPPH radical-scavenging assay.

Understanding the mechanism of action of the target enzyme/s is critical in the early discovery and development of drug candidates. In an effort to understand the plausible protein-ligand interactions on a molecular level, we performed kinetic studies on compounds **3b** and **3e** with dual inhibitory potential against AChE, BChE, and β-secretase activities.

### 2.3. Kinetic Studies of **3b** and **3e** against AChE, BChE and BACE-1

The mechanisms of action of compounds **3b** and **3e** were evaluated by constructing the Lineweaver-Burk double reciprocal plots and the Dixon plots at increasing inhibitor and substrate concentrations (0.1, 0.5, 2.5, and 5 µM). The corresponding graphs for compounds **3b** and **3e** against AChE (Figure S2), BChE (Figure S3), and β-secretase (Figure S4) form part of the Supplementary Information (SI). The Lineweaver-Burk plot for compound **3b** against AChE activity showed a relatively unchanged Michaelis constant (K_m_) of 0.14 ± 0.03 with decreasing velocity of the reaction (V_max_) value 0.014–0.004. This observation supports a non-competitive mode of inhibition, but an intercepting set of straight lines above the x-axis of the Dixon plot with a K_i_ values of 4.3 ± 0.01 indicates a competitive mode of inhibition against AChE activity. Thus, it can be concluded that **3b** displays mixed inhibition against AChE. The Lineweaver-Burk plot for compound **3e** against AChE activity showed relatively unchanged Michaelis constant (K_m_) values of 0.21 ± 0.01 with decreasing the velocity of the reaction (V_max_) 0.07–0.02. This observation supports a non-competitive mode of inhibition against AChE activity. An intercepting set of straight lines on the x-axis of the Dixon plot of **3e** with a K_i_ value of 1.6 ± 0.01 further supports the non-competitive mode of inhibition against AChE activity by this compound. Compounds **3b** and **3e** were also found to exhibit a non-competitive mode of inhibition against BChE activity with decreasing V_max_ values of 0.027–0.007 and 0.009–0.003, and relatively unchanged Michaelis constant values of 0.32 ± 0.01 and 0.27 ± 0.02, respectively. The non-competitive mode of inhibition was also confirmed by the Dixon-plot with the Ki values of **3b** and **3e** determined to be 0.75 ± 0.03 and 1.92 ± 0.01, respectively. A kinetic study was also conducted for compounds **3b** and **3e** against BACE-1 activity in the presence of increasing substrate concentrations 150, 300, and 450 nM (Figure S4). Both compounds displayed a mixed mode of inhibition with increasing K_m_ and decreasing V_max_ values, respectively. The Dixon plots for these compounds against BACE-1 revealed sets of intercepting straight lines above the x-axis, with K_i_ values of 2.3 ± 0.02 and 2.5 ± 0.01, respectively. This observation further supports a mixed mode of inhibition of the compounds against BACE-1 activity.

### 2.4. Molecular Docking Studies of Selected Active Compounds against the Enzyme Targets

Molecular docking (in silico) is frequently used to determine the hypothetical binding orientation and interaction of small molecules and drug candidates with protein targets in order to predict their affinity and activity. The most active compounds against cholinesterases and β-secretase were docked into the active pockets of AChE (PDB: 4EY7), BChE (PDB: 1P0I), and BACE-1 (PDB: 3IXJ).

#### 2.4.1. Molecular Docking Studies within the AChE and BChE Binding Sites

**Figure 2:**
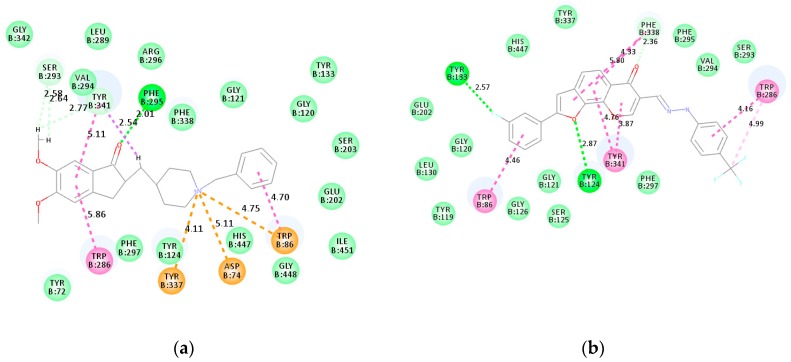

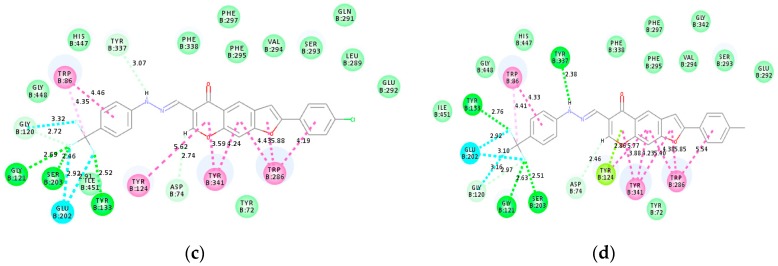
2-Dimensional (2D) docked binding poses of donepezil (**a**), **3b** (**b**), **3e** (**c**), and **3h** (**d**) into AChE showing ligand-protein residue interactions and their distances. Bright green: conventional hydrogen bonds; very light green: carbon–hydrogen bonds; dark pink: pi–pi interactions; light pink: pi–alkyl interactions; blue: halogen interaction; bright green/yellow: pi–lone pair interactions; and orange: pi–anion or cation interactions.

**Figure 3:**
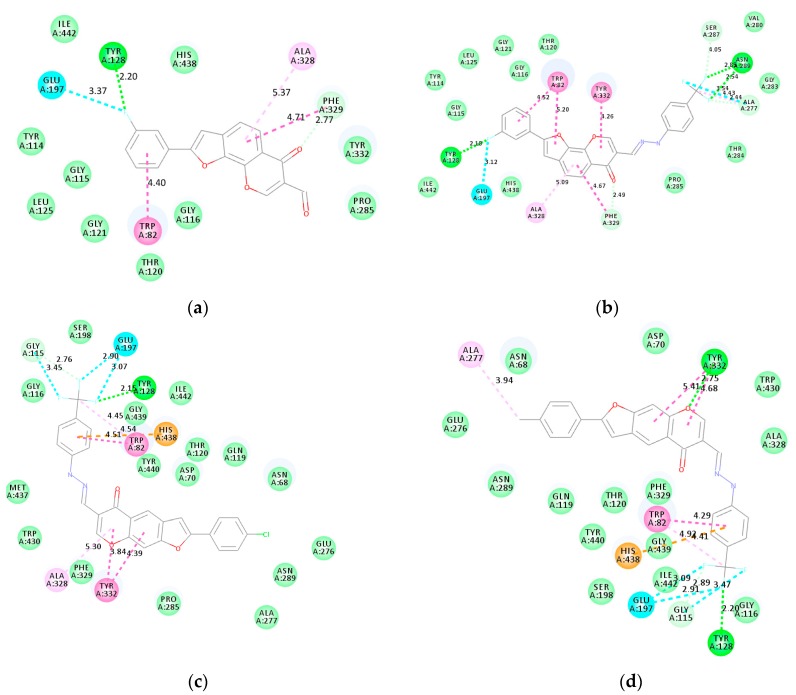
2D docked binding poses of **2b** (**a**), **3b** (**b**), **3e** (**c**), and **3h** (**d**) into BChE. Bright green: conventional hydrogen bonds; light green: van der Waals interactions; very light green: carbon–hydrogen bonds; dark pink: pi–pi interactions; light pink: pi–alkyl interactions; blue: halogen interactions; and orange: pi–anion or cation interactions.

BChE and AChE share more than 54% of their amino acid sequence and an active site located in a 20 Å deep gorge. The active site of both enzymes is divided into two sub-sites: the catalytic site (CAS) located close to the bottom of the gorge, and the peripheral anionic site (PAS) located at the entrance of the gorge [36]. CAS is composed of the catalytic triad or the esteratic site (ES), an oxyanion hole (OH), an acyl-binding pocket (ABP), and a choline binding site [37]. The crystal structure of AChE co-crystallized with donepezil was downloaded from the Protein Data Bank (PDB code: 4EY7). Donepezil was docked into the active site of this crystal and the top-scoring docked pose with the calculated binding free energy (BE) of −126.18 kcal/mol (Figure 2a) was applied as a starting point for molecular dockings into AChE binding pocket. Compounds **3b**, **3e**, and **3h** were docked individually into the active site of AChE as donepezil. The 2-dimensional docking poses of donepezil and compounds **3b**, **3e**, and **3h**, showing interactions with the AChE protein residues and their distances, are represented in Figure 2. The fluorine atom of the 3-fluorophenyl group of compound **3b** (Figure 2b) is involved hydrogen bonding interaction with Tyr133 in the anionic subsite (AS) with the phenyl ring in pi–pi stacking interaction with Trp86 of the catalytic anionic site (CAS). Hydrogen bonding interaction is also predicted between oxygen atom of the furan scaffold and Tyr124 of the peripheral anionic site (PAS). The furan and benzo rings are involved in pi–pi stacking interaction with Phe338 (ABP residue). Similar interaction also exists between the benzo and 4-pyrone rings with Tyr341 (PAS). A weak carbon–hydrogen bonding interaction is envisaged between the carbonyl oxygen and Phe338. The protein residue Trp286 (PAS) is involved in a pi–alkyl interaction with the carbon atom of the trifluoromethyl group, and also a pi–pi stacking interaction with the adjacent phenyl ring. Compounds **3e** (Figure 2c) and **3h** (Figure 2d) adopt similar docking poses within the active site of AChE. Aromatic–aromatic (pi–pi) stacking interactions exist between the 4-chloro/methylphenyl, furan, and benzo rings of **3e** and **3h** with Trp286 (PAS residue), and also between the 4-pyrone and fused benzo rings with Tyr341. The latter protein reside also forms a pi–pi stacking interaction with the furan ring of **3h**. The protein residue Tyr124 forms pi–pi, T-shaped interactions with the pyrone ring of **3e**. Tyr124 in the case of **3h** is involved in a pi–pi, T-shaped stacking interaction with the benzo ring and pi lone pair interaction with the pyranone ring. The vinylic hydrogen of the pyrone ring of both compounds is involved in pi-donor hydrogen bond interaction with Asp74 (PAS). The protein residue Tyr337 (PAS) is involved in weak carbon–hydrogen bonding interaction or conventional hydrogen bonding interaction with NH of the diazamethylidene spacer of **3e** or **3h**, respectively. The phenyl ring of the hydrazone arm is involved in pi–pi, T-shaped interactions with Trp86 (AS) for both compounds. This protein residue also forms a pi–alkyl interaction with the carbon atom of the trifluoromethyl group in both cases. There are hydrogen bonding interactions between the trifluoromethyl group of these two compounds with Gly121 (OH residue), Ser203 of the catalytic triad (CAS), and Tyr133 (AS). Halogen bonding interactions are predicted between the trifluoromethyl group of both compounds and the protein residues Gly120 and Glu202 (AS). The protein residue Gly120 is involved in a carbon–hydrogen bonding interaction with the fluorine atom of the trifluoromethyl group. The hydrazone derivatives **3b**, **3e**, and **3h** interact with protein residues in the catalytic anionic subsite (Glu202, Trp86, Tyr133) and the peripheral anionic subsite (Asn74, Trp286, Tyr337, Tyr121, Trp279, and Phe331). Compound **3b** also interacts with Phe338 in the acyl-binding pocket with no interaction of protein residues of the catalytic triad. Compounds **3e** and **3h** have no interaction with protein residues in the acyl-binding pocket. However, there is an increased number of hydrogen bonding interactions of their trifluoromethyl group with Gly121 (OH residue), Ser203 (catalytic triad) and Tyr133 (AS); their activity is consistent with their calculated binding energies (Table 5). This group is also involved in halogen bonding interactions with the protein residues Gly120 and Glu202 (AS). Increased hydrogen and halogen bonding interactions of the 4-(trifluoromethyl)phenylhydrazone wing of compounds **3e** and **3h**, including hydrogen bonding interactions with Ser203 of the catalytic triad, probably account for their significant inhibitory effects against AChE activity when compared to **3b**. These subsites are known to be buried at the bottom of a 20 Å-deep aromatic cleft. Molecular docking studies predict that these compounds will interact with protein residues in PAS and CAS like donepezil does. The dual binding of compounds to PAS and CAS of AChE is considered to be a successful example in the design of MTDLs for the effective management of AD [38].

Compounds **2b**, **3b**, **3e**, and **3h** were docked into the same active site of BChE as donepezil; the top scored docking poses of these compounds, with calculated binding free energy (BE) values of −23.24, −38.66, −60.71, and −74.00 kcal/mol, are shown in Figure 3, respectively. The 2D docking pose of donepezil (BE = −39.00 kcal/mol) into BChE showing interaction distances is included as Figure S5 in the SI. The fluorine atom of 3-fluorophenyl group of **2b** (Figure 3a) and **3b** (Figure 3b) is involved in halogen and hydrogen bonding interactions with Glu197 and Tyr128, respectively. The ring of **2b** participates in a pi–pi stacking interaction with Trp82 at the choline-binding site (PAS). The phenyl ring of this group, together with the furan ring of **3b**, are involved in pi–pi, T-shaped stacking interactions with the protein residue Trp82. The fused benzo ring of these compounds, on the other hand, is involved in a hydrophobic pi–pi stacking interaction with Phe329 in the acyl-binding pocket and a pi–alkyl interaction with Ala328 located at the choline-binding (or cation-pi) site. A weak carbon–hydrogen bonding interaction is envisaged between the carbonyl oxygen of the pyrone ring of **2b** and Phe329. The trifluoromethyl group of this compound is involved in a weak carbon–hydrogen bonding interaction and halogen bonding interaction with Ala277 of the acyl-binding pocket, as well as carbon–hydrogen bonding with Ser287 and a hydrogen bonding interaction with Asn289.

The difference between the docking poses of **3b** and **3e**, as well as **3h**, is the bent orientation of the arylhydrazone moiety and the positioning of the furanochromone framework in the large active site of BChE. The larger BChE active site allowed bent conformation to occur of the arylhydrazone moiety of **3e** (Figure 3c) and **3h** (Figure 3d). The 4-pyrone ring of **3e** is involved in a pi–alkyl interaction with Ala328 located in the choline binding site. The fused benzo and pyrone rings of **3e** and **3h** are involved in a pi–pi stacking interaction with Tyr332 of the peripheral anionic site. This protein residue in the case of **3h** is also involved in a hydrogen bonding interaction with the endocyclic oxygen atom of the pyrone ring. The 4-(trifluoromethyl)phenyl group of both compounds is involved in a pi–pi stacking interaction and a pi–cation interaction with Trp82 inside the primary binding site and His438 of the catalytic triad, respectively. A pi–alkyl interaction is also predicted between Trp82 and the carbon atom of the trifluoromethyl group of both compounds. The fluorine atoms of this group, in both cases, are involved in halogen bonding interactions with Gly115 and Glu197, respectively. Conventional hydrogen bonding interaction exists between the trifluoromethyl group of both compounds and Tyr128. The methyl carbon of the 4-tolyl group of **3h** is involved in an alkyl interaction with Ala277. This additional interaction of the tolyl moiety with Ala277 probably accounts for the low binding energy of **3h** (BE = −74.00 Kcal/mol) compared to that of **3e** (BE = −60.71 kcal/mol), with no interaction between the 4-chlorophenyl group and any of the BChE protein residues. The reverse trend in the inhibitory effect of **3h** (IC_50_ = 14.2 μM) and **3e** (IC_50_ = 9.9 μM) presumably reflects the lipophilicity of the substituent on the para position of the phenyl group on the furan ring. The difference in binding energies of **2b** (BE = −23.24 kcal/mol) and **3b** (BE = −38.66 kcal/mol) with similar alignment of the molecular frameworks within the BChE binding pocket can be attributed to increased interactions of the 4-(trifluoromethyl)phenyl group with this enzyme. Although the bent orientation of the 4-(trifluoromethyl)phenylhydrazono group of **3e** and **3h** resulted in an increased number of interactions with BChE protein residues, this conformation reduced the number of interactions of the furochromenone framework with protein residues in the active site of BChE. No interaction is predicted between the BChE residues and the furan nucleus or the 4-substituted phenyl substituent. The lack of interaction of these components with BChE residues presumably accounts for the observed less inhibitory effects of **3e** and **3h**, compared to **3b** with reduced binding strength. Interaction with Trp82 has proven to be decisive for selectivity towards BChE [39] and for potent dual binding inhibitors [40]. The predicted strong interactions of compounds **2b**, **3b**, **3e**, and **3h** with protein residues in the acyl-binding pocket or choline-binding site are consistent with their activity against these enzymes. Compound **3e**, which also interacts with His438 of CAS, would simultaneously block both the catalytic and peripheral anionic sites of AChE and the catalytic activity of BChE.

#### 2.4.2. Molecular Docking into β-secretase (PDB: 3IXJ) Binding Sites

**Figure 4:**
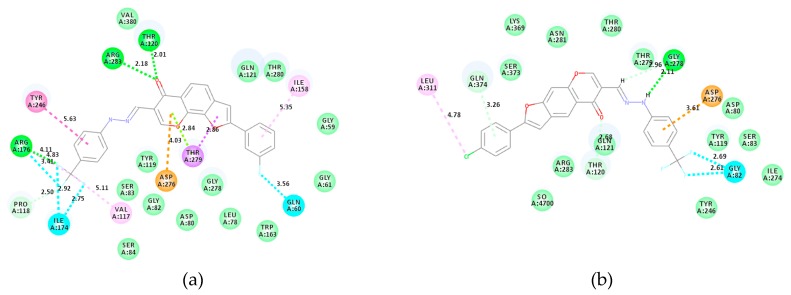
2D docked binding poses of **3b** (**a**) and **3e** (**b**) into BACE-1. Bright green represents conventional hydrogen bonds, light green: van der Waals interactions; very light green: carbon–hydrogen bonds; dark pink: pi–pi interactions; light pink: pi–alkyl interactions; purple: pi–sigma interactions; orange: pi–anion or cation interactions; blue: halogen interactions; and green yellow: pi lone pair interactions.

Quercetin was first docked into β-secretase active sites; the top scored docking pose of this ligand with BE= −32.74 Kcal/mol is included as Figure S5 in the SI. Compounds **3b** and **3e** were then docked into the same site of this enzyme as quercetin to obtain the top scored docking poses with BE values −53.61 Kcal/mol and −58.40 Kcal/mol respectively (Figure 4). These calculated binding free energy values are consistent with their IC_50_ values of 15.8 and 13.6 μM, respectively. The docking pose of **3b** shows a hydrogen bonding interaction between the fluorine atom of the 3-fluorophenyl group and Glu60 with the phenyl ring involved in a pi–alkyl bonding interaction with Ile158 (Figure 4a). The protein residue Thr279 is involved in pi–sigma and pi–lone pair bonding interactions with the furan and pyrone rings of this compound, respectively. The pyrone ring is also involved in a pi–anion bonding interaction with Asp276. The protein residues Arg283 and Thr120 are involved in a hydrogen bonding interaction with the carbonyl oxygen atom of the 4-pyrone ring. The ring of the phenylhydrazone wing is involved in a pi–pi, T-shaped interaction with Tyr246. The carbon atom of the trifluoromethyl group, on the other hand, interacts with Arg176 and Val117 via alkyl bonding interactions. Both Arg176 and Ile174 are involved in halogen bonding interactions with the trifluoromethyl group, with a hydrogen bonding interaction predicted between Arg176 and fluorine atoms. Compound **3e** adopts a different conformation, with the 4-(trifluoromethyl)phenylhydrazono wing in a bent orientation (Figure 4b). The chlorine atom of the 4-chlorophenyl group is envisaged to be involved in alkyl interaction with Leu311, while the ring itself interacts with Gly374 through a weak carbon–hydrogen bond. The carbonyl oxygen and Gln121 are involved in a carbon–hydrogen bonding interaction (Hb = 2.68 Å). The bent orientation of the phenylhydrazono moiety would facilitate a weak van der Waals interaction of the azomethine proton (-C**H**=N) with Thr279, and more importantly, a hydrogen bonding interaction of NH with Gly278 (Hb = 2.11 Å). The phenyl ring of the hydrazone functionality, on the other hand, is involved in a pi–anion interaction with Asp276. Halogen and hydrogen bonding interactions exist between the fluorine atoms of the trifluoromethyl group and Gly82. Although both **3e** and **3b** show no interactions with the catalytic aspartic acids, i.e., Asp32 and Asp228, these predictions, in our view, are consistent with the observed, non-competitive mode of inhibition by these compounds. This indicates that their binding affects the conformational dynamics of the protein and the enzyme’s ability to catalyze the reaction.

Since cyclooxyegenase-2 is also related to cancer, compounds **3b** and **3e** were screened for antigrowth effects in vitro against the breast cancer MCF-7 cell line using the 3-(4,5-dimethylthiazol-2-yl)-2,5-diphenyltetrazolium bromide (MTT) tetrazolium reduction assay (see Figure S7 in the SI). The preliminary results of this assay revealed that these compounds exhibit significant antigrowth effect properties against this cancer cell line when compared to against doxorubicin hydrochloride (IC_50_ = 1.59 ± 0.01 μM), with IC_50_ values of 4.20 ± 0.05 μM (**3b**) and 6.82 ± 0.02 μM (**3e**). Moreover, these compounds were found to be non-toxic in Hek293-T cells up to a concentration of 50 μM (see Figure S6 in the SI).

## 3. Experimental

### 3.1. General

The melting point values of the prepared compounds were recorded on a Thermocouple digital melting point apparatus (Mettler Toledo LLC, Columbus, OH, USA). The IR spectra were recorded on a Bruker VERTEX 70 FT-IR Spectrometer (Bruker Optics, Billerica, MA, USA) with a diamond ATR (attenuated total reflectance) accessory using the thin-film method. Merck kieselgel 60 (0.063–0.200 mm) (Merck KGaA, Frankfurt, Germany) was employed as a stationary phase for column chromatography. The NMR spectra were recorded as DMSO-*d*_6_ solutions on a Varian Mercury 300 MHz NMR spectrometer (Varian Inc., Palo Alto, CA, USA) operating at 300 MHz (^1^H-NMR), 75 MHz (^13^C-NMR) or 282 MHz (^19^F{^1^H}-NMR). The chemical shift values are reported relative to tetramethylsilane (TMS) as the internal standard. The low- and high-resolution mass spectra were recorded at an ionization potential of 70 eV using Micromass Autospec Time-of-Flight (double focusing high resolution) instrument (Waters Corp., Milford, MA, USA).

### 3.2. Synthesis of 3-iodo-2,4-dihydroxyacetophenone

A stirred solution of 2,4-dihydroxyacetophenone (10.0 g, 65.73 mmol) in ethanol (80 mL) and water (50 mL) was treated with potassium iodate (2.18 g, 13.13 mmol) and iodine (7.51 g, 29.59 mmol). The mixture was stirred at room temperature (RT) for 12 h, and then poured into a solution of sodium thiosulfate and ice-cold water. The product was extracted into chloroform and the combined organic phases were washed with water and dried using sodium sulfate. The salt was filtered off and the solvent was evaporated under reduced pressure. The residue was purified by column chromatography on silica gel using 30% ethyl acetate-hexane mixture as an eluent to afford 3-iodo-2,4-dihydroxyacetophenone as a solid (8.00 g, 87%), R*_f_* (toluene-ethyl acetate) 0.61, mp. 157–158 °C (Lit. [41] 164 °C); ^1^H-NMR (300 MHz, DMSO-*d*_6_) 2.56 (3H, s, -OCH_3_), 6.51 (1H, d, *J* = 8.7 Hz, H-5), 7.80 (1H, d, *J* = 8.7 Hz, H-6), 11.57 (1H, s, -OH), 13.70 (1H, s, -OH); δ_C_ (75 MHz, DMSO-*d*_6_) 26.4, 75.4, 107.5, 113.1, 133.7, 164.0, 164.8, 20.3.3.

### 3.3. Synthesis of 8-hydroxy-7-iodo-4-oxo-4H-chromene-3-carbaldehyde (**1**)

A stirred mixture of 2,4-dihydroxy-3-iodoacetophenone (5.00 g, 15.8 mmol) and DMF (5.78 g 79.1 mmol) at 0 °C was treated slowly with phosphoryl chloride (12.13 g 79.1 mmol). After 30 minutes at this temperature, the mixture was allowed to warm to RT, and stirring was continued for an additional 12 h. The reaction mixture was poured slowly onto crushed ice. The product was extracted into chloroform, and the combined organic phases were dried over anhydrous magnesium sulfate, after which the salt was filtered off. The solvent was evaporated under reduced pressure on a rotary evaporator, and the residue was purified by column chromatography on silica gel using 30% ethyl acetate-hexane mixture as an eluent to afford **1** as a brown solid (3.66 g, 73%), R*_f_* (ethyl acetate-hexane) 0.64, mp. 205–206 °C; ν_max_ (ATR) 500, 766, 856, 1292, 1605, 1679, 2763, 3052 cm^−1^; δ_H_ (300 MHz, DMSO-*d*_6_) 7.10 (1H, d, *J* = 8.1 Hz, H-8), 7.90 (1H, d, *J* = 8.1 Hz, H-2), 8.86 (1H, s, H-5), 10.05 (1H, s, -CHO); δ_C_ (75 MHz, DMSO-*d*_6_) 75.5, 114.8, 118.4, 119.9, 127.1, 157.2, 163.5, 164.1, 174.4, 189.0; HRMS (ES^+^): *m*/*z* [M + H]^+^ calculated (calc.) for C_10_H_6_O_4_I: 316.9311; found 316.9314.

### 3.4. Typical Procedure for Tandem Sonogashira Cross-Coupling–Heteroannulation of **1**

A mixture of **1** (0.50 g, 1.58 mmol), PdCl_2_(PPh_3_)_2_ (0.06 g, 0.08 mmol), CuI (0.03 g, 0.16 mmol), K_2_CO_3_ (0.33 g, 2.37 mmol), and triphenyl phosphine (0.04 g, 0.16 mmol) in 9:1 DMF-water (*v*/*v*, 20 mL) was placed in a two necked round-bottom flask equipped with a stirrer bar, condenser, and rubber septum, and then purged with nitrogen gas for 30 min A solution of phenylacetylene (0.19 g, 1.90 mmol) in DMF (1 mL) was added to the mixture by means of a syringe. A balloon filled with nitrogen gas was connected to the top of the condenser and the mixture was heated at 70 °C for 3 h under an inert atmosphere. The mixture was poured into an ice-cold water, and the product was extracted with chloroform. The combined organic layers were washed with brine, and dried over anhydrous magnesium sulfate. The salt was filtered off, and the solvent was evaporated under reduced pressure on a rotary evaporator. The residue was purified by column chromatography on silica gel using 2:1 toluene-ethyl acetate mixture as an eluent. Compounds **2a**–**i** were prepared in this fashion.

4-Oxo-8-phenyl-4*H*-furo[2,3-*h*]chromene-3-carbaldehyde (**2a**)

Brown solid (0.31 g, 68%), R*_f_* (toluene-ethyl acetate) 0.52, mp.210–211 °C; ν_max_ (ATR) 485, 523, 689, 758, 1230, 1315, 1572, 1624, 1654 (C(4)=O), 1687 (-CH=O), 2924, 3051 cm^−1^; δ_H_ (300 MHz, DMSO-*d*_6_) 7.45–7.54 (3H, m, Ar), 7.83 (1H, d, *J* = 9.3 Hz, H-9), 7.89 (1H, s, H-3), 8.00 (2H, d, *J* = 9.3 Hz, Ar), 8.01 (1H, d, *J* = 9.3 Hz, H-8), 9.07 (1H, s, H-5), 10.15 (1H, s, -CHO); δ_C_ (75 MHz, DMSO-*d*_6_) 99.5, 111.5, 119.1, 120.7, 120.9, 121.7, 125.5, 129.1, 129.7, 130.2, 150.2, 157.5, 158.0, 163.0, 175.1, 189.1; HRMS (ES^+^): *m*/*z* [M + H]^+^ calc. for C_19_H_12_O_5_: 321.0773; found 291.0652. *Anal calcd* for C_18_H_10_O_4_: C, 74.48; H, 3.47; Found: C, 74.29; H, 3.43.

8-(3-Fluorophenyl)-4-oxo-4*H*-furo[2,3-*h*]chromene-3-carbaldehyde (**2b**)

Yellow solid (0.29 g, 60%), R*_f_* (toluene-ethyl acetate) 0.51, mp. 255–256 °C; ν_max_ (ATR) 485, 686, 764, 851, 980, 1179, 1272, 1325, 1417, 1574, 1614, 1627 (C(4)=O), 1699 (-CH=O), 3115, 3090 cm^−1^; δ_H_ (300 MHz, DMSO-*d*_6_) 7.28 (2H, t, *J* = 2.7 Hz ), 7.53 (2H, dd, *J* = 8.1 Hz and 14.7 Hz), 7.77 (1H, d, *J* = 9.0 Hz, Ar), 7.90 (1H, s, H-3), 8.00, (1H, d, *J* = 8.7 Hz, H-8), 8.96 (1H, s, H-5), 10.11 (1H, s, -CHO) δ_C_ (75 MHz, DMSO-*d*_6_) 100.7, 111.5, 112.1 (d, ^2^*J*_CF_ = 23.6 Hz), 116.8 (d, ^2^*J*_CF_ = 20.8 Hz), 119.2, 120.8, 120.9, 121.5 (d, ^4^*J*_CF_ = 2.0 Hz), 122.0, 131.1 (d, ^3^*J*_CF_ = 8.62 Hz), 131.7 (d, ^3^*J*_CF_ = 8.5 Hz), 150.2, 156.1, 158.0, 162.8, 162.9 (d, ^1^*J*_CF_ = 242.7 Hz), 175.0, 188.9; HRMS (ES^+^): *m*/*z* [M + H]^+^ calc. for C_18_H_10_FO_4_: 309.0561; found 309.0563. *Anal calcd* for C_18_H_9_FO_4_: C, 70.13; H, 2.94; Found: C, 70.1; H, 2.86.

8-(4-Fluorophenyl)-4-oxo-4*H*-furo[2,3-*h*]chromene-3-carbaldehyde (**2c**)

Brown solid (0.35 g, 73%), R*_f_* (toluene-ethyl acetate) 0.50, mp. 213–214 °C; ν_max_ (ATR) 508, 592, 764, 777, 828, 1158, 1229, 1325, 1416, 1503, 1571, 1626, 1651 (C(4)=O), 1692 (-CH=O), 2924, 3072 cm^−1^; δ_H_ (300 MHz, DMSO-*d*_6_) 7.37 (2H, t, J = 8.7 Hz, H-2′,6′); 7.81 (1H, d, *J* = 9.0 Hz, H-9), 7.85 (1H, s, H-3), 8.01 (1H, d, *J* = 8.7 Hz, H-8), 8.04 (2H, dd, *J*_HH_ = 5.4 Hz and *J*_HF_ = 9.3 Hz, H-3′,5′), 9.00 (1H, s, H-5), 10.15 (1H, s, -CHO); δ_C_ (75 MHz, DMSO-*d*_6_) 99.3, 111.5, 116.7 (d, ^2^*J*_CF_ = 21.75 Hz),119.4, 120.7 (d, ^4^*J*_CF_ = 2.25 Hz), 121.6, 125.8, 127.9(d, ^3^*J*_CF_ = 8.025 Hz), 128.7, 129.3, 150.1, 156.7, 157.9, 162.9, 163.2 (d, ^1^*J*_CF_ = 246.2 Hz), 175.0, 189.0; HRMS (ES^+^): *m*/*z* [M + H]^+^ calc. for C_18_H_9_O_4_F: 309.0559; found 309.0562. *Anal calcd* for C_18_H_9_FO_4_: C, 70.13; H, 2.94; Found: C, 70.04; H, 2.54.

8-(3-Chlorophenyl)-4-oxo-4*H*-furo[2,3-*h*]chromene-3-carbaldehyde (**2d**)

White solid (0.33 g, 65%), R*_f_* (toluene-ethyl acetate) 0.50, mp. 237–238 °C; ν_max_ (ATR) 430, 454, 680, 772, 907, 962, 1306, 1559, 1636, 1634 (C(4)=O), 1691 (-CH=O), 2893, 3065 cm^−1^; δ_H_ (300 MHz, DMSO-*d*_6_) 7.49 (1H, t, *J* = 1.8 Hz, Ar), 7.51 (1H, d, *J* = 7.0 Hz, H-9), 7.80 (1H, d, *J* = 8.7 Hz, H-9), 7.91 (1H, d, *J* = 7.5 Hz, H-8), 7.96 (1H, s, H-3), 8.00–8.04 (3H, m, Ar), 8.99 (1H, s, H-5), 10.14 (1H, s, -CHO); δ_C_ (75 MHz, DMSO-*d*_6_) 100.9, 111.6, 119.2, 120.8, 121.0, 122.1, 124.0, 125.1, 129.8, 131.0, 131.5, 134.5, 150.2, 155.9, 158.0, 163.0, 175.0, 189.0; HRMS (ES^+^): *m*/*z* [M + H]^+^ calc. for C_18_H_10_^35^ClO_4_: found 325.0261 requires 325.0268. *Anal calcd* for C_18_H_9_ClO_4_: C, 66.58; H, 2.99; Found: C, 66.49; H, 2.97.

8-(4-Chlorophenyl)-4-oxo-4*H*-furo[2,3-*h*]chromene-3-carbaldehyde (**2e**)

Yellow solid (0.30 g, 58%), R*_f_* (toluene-ethyl acetate) 0.56, mp. 244–245 °C; ν_max_ (ATR) 495, 560, 766, 777, 977, 1094, 1410, 1574, 1663 (C(4)=O), 1698 (-CH=O), 3071 cm^−1^; δ_H_ (300 MHz, DMSO-*d*_6_) 7.54 (2H, d, *J* = 8.1 Hz, H-3′,5′), 7.80 (2H, d, *J* = 9.0 Hz, H-2′,6′), 7.86 (1H, s, H-3), 7.95 (1H, d, *J* = 8.7 Hz, H-9), 8.00 (1H, d, *J* = 8.7 Hz, H-8), 8.96 (1H, s, H-5), 10.13 (1H, s, -CHO); δ_C_ (75 MHz, DMSO-*d*_6_) 100.2, 111.5, 119.3, 120.7, 122.0, 127.2, 127.9, 128.4, 128.7, 129.3, 130.0, 134.6, 156.4, 158.0, 163.0, 175.0, 189.0; HRMS (ES^+^): *m*/*z* [M + H]^+^ calc. for C_18_H_10_^35^ClO_4_: 325.0261; found 325.0264. *Anal calcd* for C_18_H_9_ClO_4_: C, 66.58; H, 2.99; Found: C, 66.49; H, 2.93.

8-(4-Methoxyphenyl)-4-oxo-4*H*-furo[2,3-*h*]chromene-3-carbaldehyde (**2f**)

Brown solid (0.37 g, 74%), R*_f_* (toluene-ethyl acetate) 0.52, mp. 214–215 °C; ν_max_ (ATR) 499, 582, 777, 831, 1025, 1175, 1251, 1305, 1505, 1572, 1606, 1650 (C(4)=O), 1690 (-CH=O), 3059 cm^−1^; δ_H_ (300 MHz, DMSO-*d*_6_); 3.80 (3H, s, -OCH_3_), 7.03 (2H, d, *J* = 9.0 Hz, H-3′,5′), 7.61 (1H, s, H-3), 7.73 (1H, d, *J* = 8.7 Hz, H-9), 7.86 (2H, d, *J* = 8.4 Hz, H-2′,6′), 7.94 (1H, d, *J* = 8.7 Hz, H-8), 8.93 (1H, s, H-5), 10.13 (1H, s, -CHO); δ_C_ (75 MHz, DMSO-*d*_6_) 55.8, 97.6, 111.3, 115.1, 119.7, 120.6, 120.8, 121.0, 121.6, 127.2, 150.1, 157.8, 157.9, 160.8, 163.0, 175.2, 189.1; HRMS (ES^+^): *m*/*z* [M + H]^+^ calc. for C_19_H_12_O_5_: 321.0773; found 321.0773. *Anal calcd* for C_19_H_12_O_5_: C, 71.25; H, 3.78; Found: C, 71.03; H, 3.73.

8-(4-Methyphenyl)-4-oxo-4*H*-furo[2,3-*h*]chromene-3-carbaldehyde (**2g**)

Yellow solid (0.36 g, 76%), R*_f_* (toluene-ethyl acetate) 0.52, mp. 214–215 °C; ν_max_ (ATR) 482, 584, 726, 845, 1033, 1168, 1254, 1300, 1509, 1569, 1635, 1657 (C(4)=O), 1682 (-CH=O), 3059 cm^−1^; δ_H_ (300 MHz, DMSO-*d*_6_); 2.32 (3H, s, -CH_3_), 7.26 (2H, d, *J* = 8.1 Hz, H-3′,5′), 7.66 (1H, s, H-3), 7.73 (1H, d, *J* = 9.6 Hz, H-9), 7.79 (2H, d, *J* = 8.1 Hz, H-2′,6′), 7.94 (1H, d, *J* = 9.0 Hz, H-8), 8.92 (1H, s, H-5), 10.12 (1H, s, -CHO); δ_C_ (75 MHz, DMSO-*d*_6_) 20.8, 100.2, 111.5, 119.3, 120.7, 120.9, 121.9, 127.2, 127.9, 129.7, 130.9, 134.3, 134.6, 150.1, 156.4, 158.0, 163.0, 175.0, 189.0; HRMS (ES^+^): *m*/*z* [M + H]^+^ calc. for C_20_H_13_O_6_ 351.0869; found 351.0862. *Anal calcd* for C_20_H_14_O_6_: C, 68.57; H, 4.03; Found: C, 68.49; H, 3.99.

8-(3,5-Dimethoxyphenyl)-4-oxo-4*H*-furo[2,3-*h*]chromene-3-carbaldehyde (**2h**)

Grey solid (0.39 g, 71%), R*_f_* (toluene-ethyl acetate) 0.52, mp. 236–237 °C; ν_max_ (ATR) 575, 602, 669, 791, 863, 1053, 1363, 1581, 1631 (C(4)=O), 1673 (-CH=O), 2896, 2938 cm^−1^; δ_H_ (300 MHz, DMSO-*d*_6_); 3.81 (6H, s, -OCH_3_), 6.55 (1H,t, *J* = 2.4 Hz, H-4′), 7.10 (2H, d, *J* = 2.1 Hz, H-2′,6′), 7.80 (1H, d, *J* = 8.7 Hz, H-9), 7.94 (1H, s, H-3), 8.00 (1H, d, *J* = 8.1 Hz, H-8), 8.98 (1H, s, H-5), 10.14 (1H, s, -CHO); δ_C_ (75 MHz, DMSO-*d*_6_) 55.0 (2×C), 101.4, 102.4, 103.5, 111.6, 119.5, 120.8, 121.0, 121.8, 125.8, 128.7, 129.4, 130.9, 150.3, 157.5, 158.0, 161.5, 163.1, 175.1, 189.1; HRMS (ES^+^): *m*/*z* [M + H]^+^ calc. for C_20_H_13_O_6_: 351.0869; found 351.0862; HRMS (ES^+^): *m*/*z* [M + H]^+^ calc for C_20_H_13_O_6_: 351.0869; found 351.0862. *Anal calcd* for C_19_H_12_O_4_: C, 74.99; H, 3.97; Found: C, 74.98; H, 3.93.

8-(Cyclohex-1-en-1-yl)-4-oxo-4*H*-furo[2,3-*h*]chromene-3-carbaldehyde (**2i**)

Brown solid (0.34 g, 74%), R*_f_* (toluene-ethyl acetate) 0.52, mp. 183–184 °C; ν_max_ (ATR) 467, 560, 764, 1223, 1312, 1418, 1573, 1621, 1651 (C(4)=O), 1702 (-CH=O), 2924, 3080 cm^−1^; δ_H_ (300 MHz, DMSO-*d*_6_) 1.63 (2H, t, *J* = 1.5 Hz -CH_2_), 1.73 (2H, t, *J* = 3.9 Hz –CH_2_), 2.25 (2H, t, *J* = 3.6 Hz, -CH_2_), 2.39 (2H, t, *J* = 1.6 Hz -CH_2_), 6.64 (1H, t, *J* = 3.9 Hz -CH), 7.12 (1H, s, H-3), 7.72 (1H, d, *J* = 8.7 Hz, H-9), 8.00 (1H, d, *J* = 8.7 Hz, H-8), 8.96 (1H, s, H-5), 10.15 (1H, s, -CHO); δ_C_ (75 MHz, DMSO-*d*_6_) 21.9, 22.1, 24.6, 25.4, 97.9, 111.2, 119.2, 120.6, 121.3, 126.7, 128.6, 150.0, 157.7, 159.0, 163.0, 175.1, 189.1; HRMS (ES^+^): *m*/*z* [M + H]^+^ calc. for C_18_H_14_ O_4_; 295.0970; found 295.0968. *Anal calcd* for C_18_H_14_O_4_: C, 73.46; H, 4.79; Found: C, H.

### 3.5. Typical Procedure for the Synthesis of Hydrazones **3a**–**i** from **2a**–**i**

A stirred mixture of **2a** (0.20 g, 0.63 mmol) and pyridine (0.05 g, 0.63 mmol) in EtOH (15 mL) was treated with 4-trifluoromethylphenyhydrazine (0.13 g, 0.76 mmol). The mixture was heated under reflux for 6 h and then poured into an ice-cold water. The resulting precipitate was filtered off and purified by column chromatography on silica gel using a 40% ethyl acetate-hexane mixture as an eluent to afford compound **3a** as a brown solid. Compounds **3b–i** were prepared in a similar fashion.

2-Phenyl-6-[(4-trifluoromethylphenyl)hydrazonomethyl]-furo[3,2-*h*]chromen-5-one (**3a**)

Yellow solid (0.19 g, 64%), R*_f_* (ethyl acetate-hexane) 0.62, mp. 258–259 °C; ν_max_ (ATR) 491, 599, 759, 833, 1063, 1097, 1214, 1324, 1615 (C=N), 1633 (C=O), 3036, 3260 (NH) cm^−1^; δ_H_ (300 MHz, DMSO-*d*_6_) 7.19 (2H, d, *J* = 8.4 Hz, H-) 7.46 (1H, d, *J* = 7.5 Hz, H-8), 7.45–7.54 (5H, m, Ar), 7.81 (1H, d, *J* = 8.7 Hz, H-9), 7.90 (1H, s, H-3), 8.01 (2H, d, *J* = 8.4 Hz, Ar), 8.08 (1H, s, H-5), 8.96 (1H, s, -CH=N), 10.96 (1H, s, NH); δ_C_ (75 MHz, DMSO-*d*_6_) 99.7, 110.9, 112.2, 119.0 (q, ^2^*J* = 31.25 Hz), 120.0, 121.8, 124.4, 125.5, 126.3 (q, ^1^*J* = 274.1 Hz) 126.5, 126.9, 129.3, 129.6, 130.0, 131.2, 148.4, 150.4, 152.6, 157.3, 157.6, 175.1; δ_F_ (282.2 MHz, DMSO-*d_6_*) -59.3; HRMS (ES^+^): *m*/*z* [M + H]^+^ calc. for C_25_H_16_N_2_O_3_F_3_: 449.1113; found 449.1104. *Anal calcd* for C_25_H_15_N_2_O_3_F_3_: C, 66.97; H, 3.37; N, 6.25. Found: C, 66.94; H, 3.33; N, 6.20.

2-(3-Fluorophenyl)-6-[(4-trifluoromethylphenyl)hydrazonomethyl]furo[3,2-*h*]chromen-5-one (**3b**)

Brown solid (0.18 g, 62%), R*_f_* (ethyl acetate-hexane) 0.58, mp. 221–222 °C; (ATR) 461, 590, 621, 775, 833, 1044, 1104, 1279, 1328, 1606, 1618 (C=N), 1649 (C=O), 2362, 3044, 3261 (NH) cm^−1^; δ_H_ (300 MHz, DMSO-*d*_6_) 6.31 (2H, d, *J* = 1.8 Hz, H-2′,6′ ), 6.44 (2H, dd, *J* = 2.4 Hz and 11.1 Hz, H-3′,5′), 7.91 (3H, d, *J* = 8.7 Hz, H-8 and H-2″,6″), 8.20 (3H, d, *J* = 8.7 Hz, H-9 and H-3″,5″), 8.30 (1H, s, H-3), 9.27 (1H, s, H-5), 10.72 (1H, s, -CH=N), 12.55 (1H, s, NH); δ_C_ (75 MHz, DMSO-*d*_6_) 99.4, 111.5, 116.8 (d, ^2^*J_CF_* = 21.75 Hz), 119.3 (q, ^2^*J* = 38.8 Hz), 120.8, 121.0, 121.7, 122.0, 125.8 (d, ^4^*J_CF_* = 2.87 Hz), 127.6 (q, ^1^*J* = 266.2 Hz), 127.8, 127.9, 128.0, 131.0, 145.1, 150.2, 156.8, 158.0, 160.0, 163.2 (d, ^1^*J_CF_* = 246.5 Hz), 163.0, 175.1; δ_F_ (282.2 MHz, DMSO-*d_6_*) -59.4; HRMS (ES^+^): *m*/*z* [M + H]^+^ calc. for C_25_H_15_N_2_O_3_F_4_: 467.1019; found 467.1006. *Anal calcd* for C_25_H_14_N_2_O_3_F_4_: C, 64.38; H, 3.03; N, 6.01. Found: C, 64.37; H, 3.00; N, 5.89.

2-(4-Fluorophenyl)-6-[(4-trifluoromethylphenyl)hydrazonomethyl]furo[3,2-*h*]chromen-5-one (**3c**)

Brown solid (0.18 g, 65%), R*_f_* (ethyl acetate-hexane) 0.62, mp. 209–211 °C; ν_max_ (ATR) 512, 613, 654, 787, 831, 1062, 1105, 1168, 1229, 1316, 1321, 1508, 1601,1613 (C=N), 1643 (C=O), 2357, 3067, 3264 (NH) cm^−1^; δ_H_ (300 MHz, DMSO-*d*_6_) 6.33 (2H, d, *J* = 1.8 Hz, H-2′,6′), 6.42 (2H, dd, *J* = 2.4 Hz and 8.7 Hz, H-3′,5′), 7.90 (3H, d, *J* = 8.7 Hz, H-8 and H-2″,6″), 8.21 (3H, d, *J* = 8.7 Hz, H-9 and H-3″,5″), 8.30 (1H, s, H-3), 9.27 (1H, s, H-5), 10.72 (1H, s, -CH=N), 12.55 (1H, s, NH); δ_C_ (75 MHz, DMSO-*d*_6_) 101.4, 102.3, 112.2, 116.8 (d, ^2^*J*_CF_ = 21.87 Hz), 117.8, 118.6 (q, ^2^*J* = 43.6 Hz) 120.7, 124.4, 125.8 (q, ^1^*J* = 268.6 Hz) 126.0, (d, ^4^*J*_CF_ = 2.25 Hz), 127.8 (d, ^3^*J*_CF_ = 7.6 Hz), 128.4, 128.7, 131.4, 148.5, 153.6, 154.2, 157.3, 157.7, 163.2 (d, ^1^*J*_CF_ = 245.5 Hz), 175.5; δ_F_ (282.2 MHz, DMSO-*d_6_*) -111.1 (1F, ddd, *J* = 2.7, 9.0, 13.9), -59.4; HRMS (ES^+^): *m*/*z* [M + H]^+^ calc. for C_25_H_15_N_2_O_3_F_4_: 467.1014; found 467.1007. *Anal calcd* for C_25_H_14_N_2_O_3_F_4_: C, 64.38; H, 3.03; N, 6.01. Found: C, 64.33; H, 3.02; N, 5.87**.**

2-(3-Chlorophenyl)-6-[(4-trifluoromethylphenyl)hydrazonomethyl]furo[3,2-*h*]chromen-5-one (**3d**)

Yellow solid (0.19 g, 68%), R*_f_* (ethyl acetate-hexane) 0.59, mp. 233–234 °C; ν_max_ (ATR) 491, 597, 775, 823, 1063, 1095, 1214, 1327, 1616 (C=N), 1637 (C=O), 3049, 3265 (NH) cm^−1^; δ_H_ (300 MHz, DMSO-*d*_6_) 7.20 (2H, d, *J* = 9.0 Hz, H-2″,6″), 7.52–7.56 (4H, m, Ar), 7.81 (1H, d, *J* = 9.3 Hz, H-8), 7.99 (1H, d, *J* = 7.8 Hz, H-9), 8.03 (1H, s, H-3), 8.06 (2H, d, *J* = 8.1 Hz, H-3″,5″), 8.12 (1H, s, H-5), 8.97 (1H, s, -CH=N), 10.96 (1H, s, NH); δ_C_ (75 MHz, DMSO-*d*_6_) 101.2, 111.0, 112.3, 119.6 (q, ^2^*J* = 57.87 Hz), 122.3, 125.1, 125.7 (q, ^1^*J* = 269.9 Hz), 126.9, 128.7, 129.4, 129.7, 131.1, 131.3, 131.6, 134.5, 137.8, 148.4, 150.6, 152.7, 155.6, 157.7, 175.1; δ_F_ (282.2 MHz, DMSO-*d_6_*) -59.4; HRMS (ES^+^): *m*/*z* [M + H]^+^ calc. for C_25_H_15_N_2_O_3_F_3_^35^Cl^+^ 483.0693; 483.0723. *Anal calcd* for C_25_H_14_N_2_O_3_F_3_Cl: C, 62.19; H, 2.29; N, 5.80. Found: C, 62.14; H, 2.26; N, 5.40.

2-(4-Chlorophenyl)-6-[(4-trifluoromethylphenyl)hydrazonomethyl]furo[3,2-*h*]chromen-5-one (**3e**)

Yellow solid (0.18 g, 64%), R*_f_* (ethyl acetate-hexane) 0.60, mp. 241–242 °C; ν_max_ (ATR) 495, 603, 772, 826, 1065, 1108, 1176, 1205, 1279, 1326, 1616 (C=N), 1627 (C=O), 3047, 3263 (NH); δ_H_ (300 MHz, DMSO-*d*_6_) 7.19 (2H, d, *J* = 8.7 Hz, H-2′,6′), 7.53 (2H, d, *J* = 9.0 Hz, H-3′,5′), 7.58 (2H, d, *J* = 8.7 Hz, H-2″,6″), 7.79 (1H, d, *J* = 8.7 Hz, H-8), 7.93 (1H, s, H-3), 8.05 (2H, d, *J* = 9.0 Hz, H-3″,5″), 8.06 (1H, s, H-5), 8.23 (1H, d, *J* = 7.8 Hz, H-9), 8.94 (1H, s, -CH=N), 10.93 (1H, s, NH); δ_C_ (75 MHz, DMSO-*d*_6_) 101.0, 103.9, 112.0, 112.2, 118.6 (q, ^2^*J* = 20.6 Hz), 120.1, 121.6, 126.9, 128.1, 129.8 (q, ^1^*J* = 263.4 Hz), 131.3, 131.6, 132.0, 148.5, 153.6, 154.4, 157.3, 158.2, 161.4, 175.5; δ_F_ (282.2 MHz, DMSO-*d_6_*) -59.3; HRMS (ES^+^): *m*/*z* [M + H]^+^ calc. for C_25_H_15_N_2_O_3_F_3_^35^Cl: 483.0723; found 483.0735. *Anal calcd* for C_25_H_14_N_2_O_3_F_3_Cl _3_: C, 62.19; H, 2.29; N, 5.80. Found: C, 62.18; H, 2.25; N, 5.53.

2-(4-Methoxyphenyl)-6-[(4-trifluoromethylphenyl)hydrazonomethyl]furo[3,2-*h*]chromen-5-one (**3f**)

Brown solid (0.21 g, 70%), R*_f_* (ethyl acetate-hexane) 0.55, mp. 235–236 °C; max (ATR) 490, 599, 771, 830, 1064, 1100, 1179, 1307, 1328, 1505, 1616 (C=N), 1627 (C=O), 3270 (NH) cm^−1^, δ_H_ (300 MHz, DMSO-*d*_6_) 7.07 (2H, d, *J* = 8.4 Hz, H-2′,6′), 7.19 (2H, d, *J* = 7.5 Hz, H-3′,5′), 7.53 (2H, d, *J* = 8.4 Hz, H-2″,6″), 7.70 (1H, s, H-3),7.76 (1H, d, *J* = 8.1 Hz, H-8), 7.93 (2H, d, *J* = 8.7 Hz, H-3″,5″), 7.98 (1H, d, *J* = 9.0 Hz, H-9), 7.08 (1H, s, H-5), 8.93 (1H, s, -CH=N), 10.93 (1H, s, NH); δ_C_ (75 MHz, DMSO-*d*_6_) 55.7, 97.8, 110.8, 112.2, 115.0, 115.1, 119.8 (q, ^2^*J* = 34.2 Hz), 121.2, 122.0, 126.8, 126.9, 127.0, 127.2 (q, ^1^*J* = 268.7 Hz), 131.2, 148.5, 150.2, 152.6, 157.4, 157.6, 160.8, 175.2; δ_F_ (282.2 MHz, DMSO-*d_6_*) -59.4; HRMS (ES^+^): *m*/*z* [M + H]^+^ calc. for C_26_H_18_N_2_O_4_F_3_ 479.1219; found 479.1223. *Anal calcd* for C_26_H_17_N_2_O_4_F_3_: C, 65.27; H, 3.58; N, 5.86. Found: C, 65.07; H, 3.54; N, 5.85.

2-(3,5-Dimethoxyphenyl)-6-[(4-trifluoromethylphenyl)hydrazonomethyl]furo[3,2-*h*]chromen-5-one (**3g**)

Yellow solid (0.16 g, 60 %), R*_f_* (ethyl acetate-hexane) 0.50, mp. 220–222 °C; ν_max_ (ATR) 514, 644, 790, 843, 1076, 1101, 1166, 1212, 1250, 1301, 1481, 1511, 1549, 1615 (C=N), 1654, (C=O), 2923, 3074, 3242 (NH)cm^−1^; δ_H_ (300 MHz, DMSO-*d*_6_) 3.82 (3H, s, -OCH_3_), 3.83 (3H, s, -OCH_3_), 6.56 (1H,t, *J* = 2.4 Hz, H-4′), 7.15 (2H, d, *J* = 2.4 Hz, H-2′,6′), 7.19 (2H, d, *J* = 8.7 Hz, H-2″,6″), 7.53 (2H, d, *J* = 9.0 Hz, H-3″,5″), 7.79 (1H, d, *J* = 8.7 Hz, H-9), 7.94 (1H, s, H-3), 8.00 (1H, d, *J* = 8.7 Hz, H-8), 8.02 (1H, s, H-5), 8.92 (1H, s, -CH=N), 10.94 (1H, s, NH); δ_C_ (75 MHz, DMSO-*d*_6_) 55.7 (2 × C), 98.5, 98.5, 104.2, 110.8, 112.2, 115.0, 120.0 (q, ^2^*J* = 55.0 Hz), 121.9, 122.3, 125.3 (q, ^1^*J* = 268.7 Hz), 127.2, 127.4, 128.4, 131.2, 132.7, 142.2, 143.6, 148.5, 152.6, 155.9, 156.9, 158.8, 160.8, 175.2; δ_F_ (282.2 MHz, DMSO-*d_6_*) -59.3; HRMS (ES^+^): *m*/*z* [M + H]^+^ calc. for C_27_H_20_N_2_O_5_F_3_: 509.1324; found 509.1311. *Anal calcd* for C_27_H_19_N_2_O_5_F_3_: C, 63.78; H, 3.77; N, 5.51. Found: C, 63.73; H, 3.74; N, 5.48.

2-(*p*-Tolyl)-6-[(4-trifluoromethylphenyl)hydrazonomethyl]furo[3,2-*h*]chromen-5-one (**3h**)

Yellow solid (0.15 g, 69 %), R*_f_* (ethyl acetate-hexane) 0.66, mp. 249–251 °C; max (ATR) 490, 593, 773, 835, 1063, 1099, 1157, 1211, 1276, 1327, 1417, 1616 (C=N), 1630 (C=O), 3258 (NH) cm^−1^, δ_H_ (300 MHz, DMSO-d6) 7.18 (2H, d, *J* = 8.4 Hz, H-2′,6′), 7.33 (2H, d, *J* = 8.1 Hz, H-3′,5′), 7.52 (2H, d, *J* = 9.0 Hz, H-2″,6″), 7.79 (1H, s, H-3),7.91 (1H, d, *J* = 8.4 Hz, H-8), 8.00 (1H, d, *J* = 9.3 Hz, H-9), 7.08 (1H, s, H-5), 823 (2H, d, *J* = 7.8 Hz, H-3″,5″), 8.93 (1H, s, -CH=N), 10.93 (1H, s, NH); δ_C_ (75 MHz, DMSO-*d*_6_) 31.1, 99.7, 101.9, 119.8 (q, ^2^*J* = 55.0 Hz), 122.3, 124.0, 125.1, 125.8, 126.9 (q, ^1^*J* = 269.1 Hz), 128.7, 129.4, 130.0, 131.1, 134.5, 148.4, 150.4, 152.7, 157.2, 157.6, 161.2, 175.1; δ_F_ (282.2 MHz, DMSO-*d_6_*) -59.4; HRMS (ES^+^): *m*/*z* [M + H]^+^ calc. for C_26_H_18_N_2_O_3_F_3_: 460.1267; found 460.1270. *Anal calcd* for C_26_H_17_N_2_O_3_F_3_: C, 67.53; H, 3.71; N, 6.06. Found: C, 67.51; H, 3.57; N, 6.02.

2-(Cyclohex-1-en-1-yl)-6-((2-(4-(trifluoromethyl)phenyl)hydrazono)methyl)-5*H*-furo[3,2-*h*]chromen-5-one (**3i**)

Brown solid (0.20 g, 66 %), R*_f_* (ethyl acetate-hexane) 0.63, mp 235–237 °C;ν_max_ (ATR) 593, 632, 787, 829, 1053, 1104, 1175, 1223, 1246, 1282, 1465, 1506, 1546, 1606, 1617 (C=N), 1635 (C=O), 2837, 2932, 3065, 3249 (NH) cm^−1^; δ_H_ (300 MHz, DMSO-*d*_6_) 1.63 (2H, t, *J* = 1.5 Hz -CH_2_), 1.70 (2H, t, *J* = 3.9 Hz –CH_2_), 2.22 (2H, t, *J* = 3.6 Hz, -CH_2_), 2.34 (2H, t, *J* = 1.6 Hz -CH_2_), 6.60 (1H, t, *J* = 3.9 Hz -CH), 6.69 (1H, s, H-3), 7.16 (2H, d, J = 8.4 Hz, (2H, d, *J* = 8.4 Hz, H- H-2″,6″), 7.51 (2H, d, *J* = 8.4 Hz, H-3″,5″), 7.72 (1H, d, *J* = 8.7 Hz, H-9), 8.00 (1H, d, *J* = 8.7 Hz, H-8), 8.93 (1H, s, -CH=N), 10.87 (1H, s, NH); δ_C_ (75 MHz, DMSO-*d*_6_) 21.9, 22.1, 24.7, 25.4, 101.1, 118.9, 119.5 (q, ^2^*J* = 17.2 Hz), 120.0, 121.8, 123.7, 124.9 (q, ^1^*J* = 271.4 Hz), 125.5, 126.9, 127.3, 129.3, 129.6, 130.0, 153.9, 157.2, 160.4, 163.8, 175.5; δ_F_ (282.2 MHz, DMSO-*d_6_*) -59.1; HRMS (ES^+^): *m*/*z* [M + H]^+^ calc. for C_25_H_20_N_2_O_3_F_3_: 453.1426; found 453.1421. *Anal calcd* for C_25_H_19_N_2_O_3_F_3_: C, 66.37; H, 4.23; N, 6.19. Found: C, 66.34; H, 4.02; N 5.98.

### 3.6. Biological Activity Studies

#### 3.6.1. Cholinesterase Enzyme Assays of **2a**–**i** and **3a**–**i**

The following were purchased through BIOCOM Africa (Pty) Ltd (Biocom Africa, Pretoria, South Africa): 768104 BL Recombinant human AChE (carrier-free) and RPC348Hu0 2_100 CLC Recombinant BChE (Type, cloud-clone corp.). Their anti-AChE and BChE activities were determined by spectrophotometric Ellman’s method [42]. The stock solutions of the test compounds (200 µM) were prepared in a 2:8 DMSO-water mixture (*v*/*v*) followed by dilution with 50 mM tris buffer (pH 7.7) to obtain final assay concentrations of 10, 25, 50, and 100 µM. The assays were conducted in triplicate using donepezil and galantamine as positive controls (10, 25, 50, and 100 µM), respectively. One microliter (1.0 µL) of the respective enzyme, 9.0 µL of the test compounds (various concentrations of **2b**, **2f**, **3b**, **3d**, **3f**, and controls, respectively), and 70 µL of tris buffer were added into the corresponding wells of a 96-well plate with the aid of a multi-channel pipette. The mixtures were incubated for 15 min at RT. The reaction was initiated by adding 10 µL of a mixture solution of 5 mM 5,50-dithio-bis-nitrobenzoic acid (DTNB) in 50 mM tris buffer (pH 7.7) and 10 µL acetylthiocholine (ASCh) (5 mM) in 50 mM tris buffer, pH 7.7. The hydrolysis of ASCh was determined by monitoring the formation of the yellow 5-thio-2-nitrobenzoate anion resulting from reaction of DTNB with thiocholines (SCh). The mixtures were further incubated for 5 min at RT, and its absorbance was measured at a wavelength of 405 nm using Varioskan flash microplate spectrophotometer (Thermo Scientific, Waltham, MA, USA). Inhibition of BChE was measured in the same manner as described for AChE. The percentage of inhibition was calculated by the following equation:

$$\mathrm{Inhibition}\;\mathrm{activity}\;\left(\%\right)=\;1/4\frac{\mathrm{Absorbance}\;\mathrm{of}\;\mathrm{control}\;-\;\mathrm{Absorbance}\;\mathrm{of}\;\mathrm{sample}nx}{\mathrm{Absorbance}\;\mathrm{of}\;\mathrm{control}}\times 100$$

The IC_50_ values were determined graphically from inhibition curves (inhibitor concentration and absorbance) using the graph pad prism.

#### 3.6.2. β-Secretase Assays of Compounds **3b** and **3e**

The β-secretase inhibitory activities of compounds **3b** and **3e** were evaluated by a fluorescence resonance energy transfer (FRET) assay (Pan Vera) with a recombinant baculovirus-expressed β-secretase and a specific substrate (Rh-EVNLDAEFK-Quencher), according to the manufacturer’s instructions. A mixture of human recombinant BACE-1 (1.0 U/mL), the substrate (75 µM in 50 mM ammonium bicarbonate), and test compound (various concentration of **3b**, **3e**, and quercetin, respectively) dissolved in an assay buffer (50 mM sodium acetate, pH 4.5) was incubated for 60 min at 25 °C in a 96-well plate. The assays were performed in triplicate, and the increase in fluorescence intensity produced by the substrate was observed on a fluorescence microplate reader with an excitation wavelength of 545 nm and an emission wavelength of 590 nm. The inhibition ratio was calculated using the following equation:

$$\mathrm{Inhibition}\left(\%\right)\;=\;\frac{1\;-\;\left(S-S_{0}\right)}{\left(C-C_{0}\right)}\times 100$$

where C is the fluorescence of control (enzyme, assay buffer, and substrate) after 60 min of incubation, C_0_ is the fluorescence of control at time 0, S is the fluorescence of tested samples (enzyme, sample solution, and substrate) after 60 min of incubation, and S_0_ the fluorescence of the tested samples at time 0.

#### 3.6.3. COX-2 Assays for Compounds **2a**–**i** and **3a**–**i**

The reagents for the COX-2 assay were prepared according to the standard protocol enclosed in the Cyclooxygenase inhibitor screening kit (Item No. K547; Bio Vision, Milpitas Blvd, Milpitas, CA USA). Stock solutions (200 µM) of the test compounds were prepared in DMSO and then diluted with tris buffer (50 Mm, pH 7.7) to obtain final assay concentrations of 0.001, 0.01, 0.1, 1, 10, and 100 µM. All assays were performed in duplicate against celecoxib as a positive control. The assay mixtures in a 96-well plate were distributed as follows: two wells taken as enzyme control (10µL assay buffer) and two wells as inhibitor control (2 µL of celecoxib and 8 µL of COX assay buffer). Ten microliters of the test compounds concentrations and those of celecoxib were added to the remaining wells with the aid of a multi-channel pipette. Then, 80 µL of the reaction mix (a mixture of 76 µL COX assay buffer, 1 µL of COX probe, 2 µL of diluted COX cofactor and 1 µL of COX-2 enzyme) were added to the reaction mixture. The assay mixtures in the 96-well plate were incubated for 5 min. The reactions were initiated by the addition of 10 µL of diluted arachidonic acid and sodium hydroxide solution (10 µL of arachidonic acid and 90 µL of sodium hydroxide) to each well. The absorbance was measured using a Varioskan flash microplate spectrophotometer, and the IC_50_ was calculated with the aid of a graph pad prism

#### 3.6.4. Soybean LOX-15 Inhibitory Assay of Compounds **2a**–**i** and **3a**–**i**

The assay was conducted as described in the literature [43] with some modification; each compound was tested in duplicate with quercetin as a reference standard. The reagents were prepared according to the standard protocol (Lipoxygenase inhibitor screening assay kit, Item No. 760700. Cayman Chemicals, Ann Arbor, MI, USA). Stock solutions of the test samples prepared in DMSO were further diluted with 50 mM Tris buffer of pH 7.7 to obtain concentrations of 0.01, 0.1, 1, 10, and 100 µM. The cells of a 96-well plate where arranged as follows: two wells taken as blanks (100 µL 50mM Tris buffer pH, 7.7), two wells taken as enzyme controls (90 µL of 15-LO Standard and 10 µL of DMSO), and two as 100% initial activity wells (90 µL lipoxygenase enzyme solution and 10 µL tris buffer). Then, 90 µL of lipoxygenase enzyme solution and 10 µL of each test compound (and reference standards) from the above concentrations were added to the other wells of a 96-well plate, and the plate was incubated for 5 min at room temperature. The reaction was initiated by addition of 10 µL of the arachidonic acid to each well containing the assay mixtures. The 96-well plate was shaken for 5 min on a shaker, and 100 µL of the chromogen (solution of equal volume developing reagent 1 and 2) was added to each well to initiate the reaction. The plate was again shaken for 5 min and the absorbance read at 490 nm in a Varioskan flash microplate spectrophotometer reader. The inhibitory concentration was expressed as a percentage using the formula below.

$$\mathrm{Inhibitory}\;\mathrm{concentration}\;=\;\frac{A_{\mathrm{Initial}\;\mathrm{activity}}\;-\left(A_{\mathrm{Sample}}\;-{\;A}_{\mathrm{Blank}\;\mathrm{sample}}\right)}{A_{\mathrm{Initial}\;\mathrm{activity}}}\times 100$$

where A_Sample_ is the absorbance of the reaction mixture of the test sample, A_Blank_ sample is the absorbance of the reaction mixture containing all reagents except enzyme, and A_Initial activity_ is the absorbance of 100% initial activity.

#### 3.6.5. Human LOX-5 Inhibitory Assay of Compounds 2f–h, 3b and 3e–g

The reagents were prepared according to the Lipoxygenase inhibitor screening kit (Item No. K980; BioVision, Milpitas Blvd, Milpitas, CA, USA) standard protocol. Stock solutions of the test compounds were prepared in DMSO, and further diluted with 50 mM Tris HCl buffer (pH 7.7) to obtain final concentrations of 0.01, 0.1, 1, 5, and 10 µM. Each compound and the reference standards (quercetin and zileuton) were tested in duplicate for this assay. The wells of a 96-well plate were arranged as follows: two wells were taken as blanks (40 µL of assay buffer) and two as enzyme controls (2 µL of LOX-5 Standard and 38 µL of tris buffer). Two microliters (2 µL) of the test compounds (various concentration of test compounds and reference standards, respectively) followed by 38 µL of LOX assay buffer were added to the other wells of the 96-well plate in duplicate. Then, 40 µL of the reaction mixture (prepared by mixing thoroughly 36 µL of LOX assay buffer, 2 µL of LOX probe and 2 µL of LOX-5 enzyme) was added to each well containing the test mixtures, and the wells were incubated for 10 min at RT. The DMSO concentration in each reaction well was 1% to prevent enzyme structural deformation and, therefore, reduced activity. The reaction was initiated by adding 20 µL of LOX substrate solution (prepared by diluting LOX substrate in a ratio 1:25 with assay buffer) to each well, and the absorbance was read at 234 nm using a Varioskan flash microplate spectrophotometer reader. The percentage inhibition (%) was calculated using the equation below.

$$\%\;\mathrm{Inhibition}\;=\;\frac{\mathrm{Control}\;-\;\mathrm{Test}}{\mathrm{Control}}\times 100$$

### 3.7. Kinetic Studies AChE, BChE and BACE-1

#### 3.7.1. Kinetic Studies of **3b** and **3e** Against AChE and BChE

Kinetic evaluations of compounds **3b** and **3e** against these enzymes at concentrations, 0.1, 0.5, 2.5, and 5 µM, were conducted following procedures described in our previous investigation [44]. The Lineweaver–Burk plots (plots of the inverse of velocity against the inverse of the substrate concentration) were used to ascertain the mode of inhibition of these compounds. The Dixon plots of the inverse of velocity (1/v) against the concentration of the inhibitor at each concentration of substrate were used to determine their inhibitor constants (K_i_).

#### 3.7.2. Kinetic Studies of **3b** and **3e** Against β-Secretase

Compounds **3b** and **3e** were selected for kinetic studies with substrate concentrations 150, 300, and 450 nM for β-secretase following the procedure described in our previous investigation [44]. The Lineweaver–Burk plot (plot of the inverse of velocity (1/v) against the inverse of the substrate concentration (1/[S])) was used to ascertain the mode of inhibition of this compound. The plot of 1/v against concentration of inhibitor at each concentration of substrate (the Dixon plot) was used to determine the inhibitor constant (K_i_).

### 3.8. DPPH Assays of Compounds **2a**–**i** and **3a**–**i**

The DPPH radical scavenging activity of compounds **2a**–**i** and **3a**–**i** was evaluated following the literature method by Zhu et al. [45]. The compounds were evaluated against ascorbic acid (Sigma Aldrich, Saint Louis, Missouri, USA) as a positive control. Various concentrations of the test compounds and control (0 µM to 40 µM) in DMSO were mixed with a solution of DPPH (0.20 mM) in methanol. The mixtures were incubated in the dark for 45 min and then the absorbances were recorded at 512 nm using Varioskan flash microplate spectrophotometer reader. All tests and analyses were run in triplicate and averaged. The inhibition was calculated in terms of percentage using the formula below:

$$\mathrm{DPPH}\;\mathrm{radical}\;\mathrm{scavenged}\;\left(\%\right)=\frac{\mathrm{AbC}\;-\;\mathrm{AbS}}{\mathrm{AbC}}\times 100$$

where AbC is absorbance of control and AbS the absorbance of the test sample. A graph of percentage inhibition of free radical activity was plotted against the concentration of the sample, and the IC_50_ value (compound concentration required to reduce the absorbance of the DPPH control solution by 50%) was obtained from the graph.

### 3.9. Molecular Docking Studies into AChE, BChE and BACE-1 Active Sites

Molecular dockings were performed using the CDOCKER module of Discovery studio software (version 17.1.0.16143; Accelrys, San Diego, CA, USA). The Protein Data Bank (PDB) structures used were as follows: 4EY7 for AChE, 1P0I for BChE and 3IXJ for β-Secretase. The compounds were drawn in discovery studio and then prepared using default parameters prior to docking. The proteins structures were prepared prior to docking using default settings of Discovery Studio without the co-crystalized ligands. The binding sites used to dock compounds represented co-crystalized ligands or substrate locations, as identified by Discovery Studio software. The x, y, and z coordinates for the binding spheres were as follows: 4EY7 (8.873, −59.10, −25.65), 1POI (135.617, 115.53, 38.362), and 3IXJ (−1.47841, 15.4838, 32.8487). The resulting docking poses were ranked according to CDOCKER and CDOCKER interaction energies, and the top-scoring pose without unfavorable interactions was selected and represented as 2D plots using Discovery Studio. Binding energies were calculated for the best-scoring pose using the calculate binding energy tool with the default settings.

### 3.10. Cytotoxicity Studies of **3b** and **3e** Against the Hek293-T Cells

Briefly, the Hek293-T cells and MCF-7 cancer cells were seeded in a 96-well plate at a density of 20 × 10^3^ cells per well, and then incubated at 37 °C in 5% CO_2_ to allow cell attachment. The medium was removed and replaced with fresh medium containing various concentrations of the test sample and doxorubicin hydrochloride (10, 25, 50, 100, and 200 μM) and incubated for 24 h. After treatment for 24 h, 10 μL MTT (5 mg/mL) was added to each well and the plate was further incubated for 4 h. The supernatant was removed, and 100 μL of DMSO was added to each well to dissolve the resulting formazan crystals. The absorbance was read at 570 nm using the Varioskan flash microplate spectrophotometer reader and the cell viability. The percentages of cell viability were used to determine the IC_50_ values.

## 4. Conclusions

The replacement of the carbaldehyde moiety with a 4-(trifluoromethyl)phenylhydrazono group resulted in dual inhibition of cholinesterases (AChE and BChE) and β-secretase by the angular furochromone derivatives **3b** and **3e**. Kinetic studies suggest a mixed mode and a non-competitive inhibitory mode of action of these compounds due to simultaneous binding to CAS and PAS of AChE. These observations have been corroborated by molecular docking studies, which predict interactions of **3b** and **3e** with several protein residues in PAS and CAS. The presence of the diazamethylidene (-CH=N-NH-) spacer in these compounds helps anchor the conformations with the 4-(trifluoromethyl)phenylhydrazono group in linear or bent orientation into the active site of AChE or BChE. Aromatic-aromatic (pi–pi) stacking and pi–cation interactions, and halogen and hydrogen bonding interactions help to stabilize the conformation of the molecules in the enzyme active sites to enhance their inhibitory effect. Compounds **2f** and **3g** were less active against cholinesterases, but have the potential to serve as anti-inflammatory agents due to their increased activity against COX-2, LOX-5, and LOX-15. In general, the presence of bulky group/s on the phenyl substituent such as 4-methoxyphenyl (**2f**), 3,5-dimethoxyphenyl (**2g**), and 4-tolyl group (**2h**) favors activity against LOX-15 compared to LOX-5 for the carbaldehyde and the 4-methoxyphenyl– **3f** and 3,5-dimethoxyphenyl–substituted hydrazone derivative **3g**. Preliminary cytotoxicity results showed that compounds **3b** and **3e** exhibit significant antigrowth effect against the breast cancer MCF-7 cell line and no toxicity in the Hek293-T cells. The results described in this investigation expand our knowledge of multipotent compounds by showing the possibility of the 4-(trifluoromethylphenyl)hydrazono-substituted furochromone **3e** concurrently inhibiting several targets involved in AD, namely, ChEs, BACE-1, COX, and LOX, including oxidative stress. This compound represents a potential multi-target-directed ligand suitable for pharmacological application in the field of neurodegenerative and inflammatory diseases, including cancer. Further studies are required to determine whether this compound or its analogues have the same effects in vivo, and to clarify the underlying mechanisms.

## Figures and Tables

**Table 1:** Substitution pattern and percentage yields of **2a**–**i** and **3a**–**i**.

Entry	R	%Yield of 2	%Yield of 3
1	C_6_H_5_-	68 (**2a**)	64 (**3a**)
2	3-FC_6_H_4_-	60 (**2b**)	62 (**3b**)
3	4-FC_6_H_4_-	63 (**2c**)	65 (**3c**)
4	3-ClC_6_H_4_-	75 (**2d**)	68 (**3d**)
5	4-ClC_6_H_4_-	58 (**2e**)	64 (**3e**)
6	4-MeOC_6_H_4_-	74 (**2f**)	70 (**3f**)
7	3,5-MeO(C_6_H_3_)-	76 (**2g**)	60 (**3g**)
8	4-MeC_6_H_4_-	71 (**2h**)	69 (**3h**)
9	-Cyclohex-1-en-1-yl	74 (**2i**)	66 (**3i**)

**Table 2:** AChE and BChE inhibitory activities of compounds **2a**–**i** and **3a**–**i**.

Compound	IC_50_ (µM)		
	AChE	BChE	SI
**2a**	25.1 ± 0.04	34.2 ± 0.02	0.8
**2b**	15.2 ± 0.03	9.2 ± 0.01	1.7
**2c**	30.4 ± 0.01	28.3 ± 0.03	1.1
**2d**	19.8 ± 0.03	13.2 ± 0.05	1.5
**2e**	27.1 ± 0.04	32.6 ± 0.02	0.8
**2f**	34.2 ± 0.02	21.8 ± 0.02	1.6
**2g**	25.3 ± 0.02	23.2 ± 0.04	1.1
**2h**	20.9 ± 0.01	25.9 ± 0.03	0.8
**2i**	15.3 ± 0.03	31.6 ± 0.02	0.5
**3a**	15.4 ± 0.01	18.7 ± 0.03	0.8
**3b**	10.4 ± 0.02	7.2 ± 0.01	1.4
**3c**	18.1 ± 0.01	15.6 ± 0.02	1.2
**3d**	24.3 ± 0.03	30.1 ± 0.03	0.8
**3e**	5.4 ± 0.02	9.9 ± 0.04	0.5
**3f**	18.4 ± 0.05	27.6 ± 0.04	0.7
**3g**	22.3 ± 0.02	30.5 ± 0.03	0.7
**3h**	9.1 ± 0.02	14.2 ± 0.01	0.6
**3i**	13.3 ± 0.04	17.9 ± 0.03	0.7
Donepezil	0.02 ± 0.03	4.7 ± 0.01	0.004
Galantamine	0.01 ± 0.01	3.6 ± 0.02	0.003

Selectivity index (SI) means IC_50_ (AChE)/IC_50_ (BChE).

**Table 3:** β-Secretase inhibitory activity of compounds **3b** and **3e**.

Compound	IC_50_ (µM) BACE-1
**3b**	15.8 ± 0.01
**3e**	13.6 ± 0.02
Quercetin	12.9 ± 0.01

**Table 4:** Activity of **2a**–**i** and **3a**–**i** against COX-2 and LOX-15; **2f**–**h**, **3f**, and **3g** against LOX-5 and anti-oxidant activity of **2a**–**i** and **3a–i**.

Compound	IC_50_ Values in µM			
	COX-2	LOX-15	LOX-5	DPPH
**2a**	25.6 ± 0.01	12.5 ± 0.03	-	10.1 ± 0.03
**2b**	43.7 ± 0.01	18.6 ± 0.01	-	27.6 ± 0.02
**2c**	15.8 ± 0.03	14.3 ± 0.01	-	7.5 ± 0.02
**2d**	23.1 ± 0.02	22.9 ± 0.02	-	29.1 ± 0.01
**2e**	18.6 ± 0.01	15.2 ± 0.04	-	19.9 ± 0.01
**2f**	13.7 ± 0.04	8.2 ± 0.03	17.3 ± 0.01	14.3 ± 0.02
**2g**	23.5 ± 0.03	10.6 ± 0.04	24.6 ± 0.02	8.3 ± 0.02
**2h**	19.8 ± 0.03	9.2 ± 0.03	28.7 ± 0.03	11.6 ± 0.03
**2i**	12.1 ± 0.01	21.5 ± 0.02	-	15.9 ± 0.02
**3a**	29.7 ± 0.02	16.3 ± 0.01	-	15.3 ± 0.01
**3b**	31.7 ± 0.04	24.6 ± 0.01	34.1 ± 0.01	18.7 ± 0.03
**3c**	41.8 ± 0.04	30.8 ± 0.04	-	31.3 ± 0.01
**3d**	30.2 ± 0.03	11.8 ± 0.03	-	32.5 ± 0.03
**3e**	10.4 ± 0.03	14.9 ± 0.04	28.5 ± 0.02	25.9 ± 0.05
**3f**	14.7 ± 0.01	6.1 ± 0.01	32.5 ± 0.02	19.1 ± 0.04
**3g**	17.6 ± 0.02	9.4 ± 0.02	19.1 ± 0.04	24.6 ± 0.01
**3h**	22.4 ± 0.01	18.6 ± 0.03	-	16.3 ± 0.03
**3i**	13.6 ± 0.01	43.9 ± 0.01	-	20.1 ± 0.01
Celecoxib	7.2 ± 0.01	-	-	-
Quercetin	-	3.3 ± 0.01	10.2 ± 0.01	-
Zileuton	-	-	11.8 ± 0.02	-
Ascorbic acid	-	-	-	4.6 ± 0.01

**Table 5:** Calculated binding energy values for **3b**, **3e**, and **3h**.

Compound	BE (kcal/mol)
**3b**	−53.33
**3e**	−67.77
**3h**	−61.77
Donepezil	−126.18

## Supplementary Materials

The following are available online at https://www.mdpi.com/2218-273X/9/11/736/s1. Figure S1: Copies of ^1^H- and ^13^C-NMR spectra of compounds **1**, **2a**–**i** and **3a**–**i**, and Figure S2: the Lineweaver-Burk and Dixon plots of **3b** and **3e** against AChE, Figure S3: BChE and Figure S4: BACE-1, Figure S5: docking poses of donepezil into BChE and Figure S6: quercetin into BACE-1 as well as Figure S7: cytotoxicity graphs for **3b**, **3e** and doxorubicin against Hek293-T cells.
