# Supplementary material for: Exploring Biological Activity of 4-Oxo-4H-furo[2,3-h]chromene Derivatives as Potential Multi-Target-Directed Ligands Inhibiting Cholinesterases, β-Secretase, Cyclooxygenase-2, and Lipoxygenase-5/15
Source: Biomolecules. 2019 Nov 13;9(11):736. doi: 10.3390/biom9110736 (PMC6920776; doi:10.3390/biom9110736)
Supplement: Supplementary file 1 [file biomolecules-09-00736-s001.pdf]

# Supplementary Materials

**Figure S1:** Copies of $^{1}$H-NMR and $^{13}$C-NMR spectra for compounds **1**, **2a–I** and **3a–i**.

**Figure S2:** Lineweaver-Burk and Dixon plots of **3b** and **3e** against AChE activity.

**Figure S3:** Lineweaver-Burk and Dixon plots of **3b** and **3e** against BChE activity.

**Figure S4:** Lineweaver-Burk and Dixon plots of **3b** and **3e** against BACE-1 activity.

**Figure S4:** Docking pose of donepezil showing interaction with BChE protein residues.

**Figure S5:** Docking pose of quercetin showing interaction with BACE-1 protein residues.

**Figure S6:** Evaluation of toxicity of **3b** and **3e** in Hek293-T cells.

**Figure S1: Copies of $^{1}$H-NMR and $^{13}$C-NMR spectra for compounds 1, 2a–i and 3a–i.**

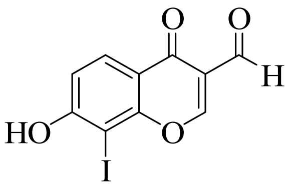


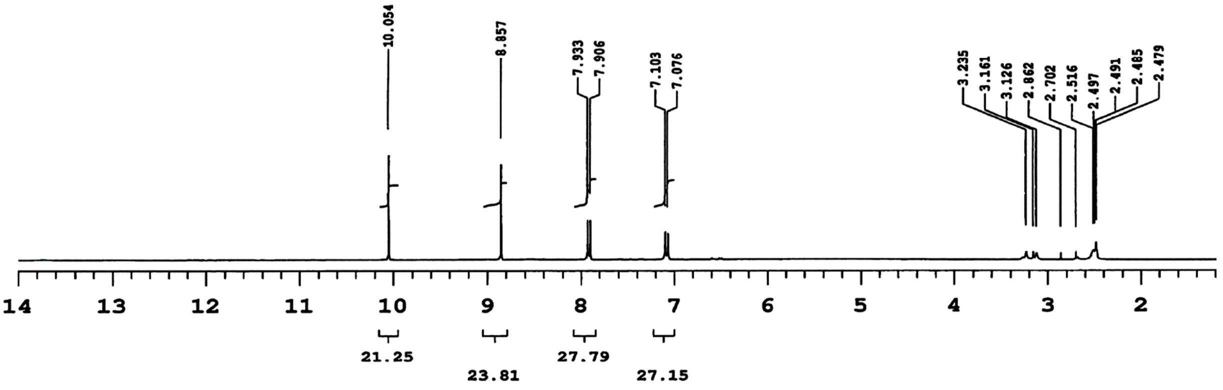


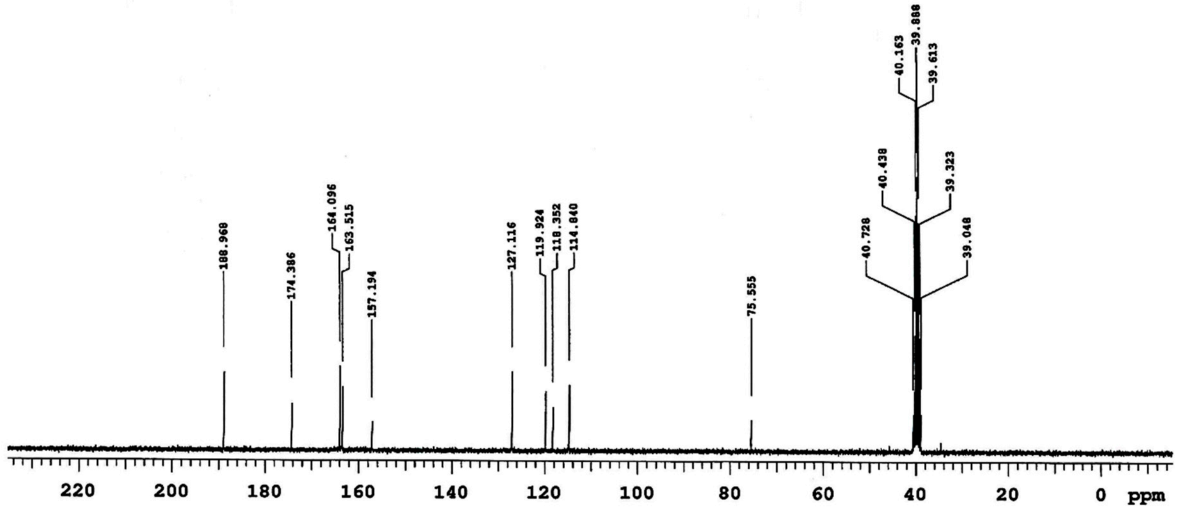


**Figure S1:** $^{1}$H- and $^{13}$C-NMR spectra of **1** in DMSO-$d_6$ at 300 and 75 MHz, respectively.

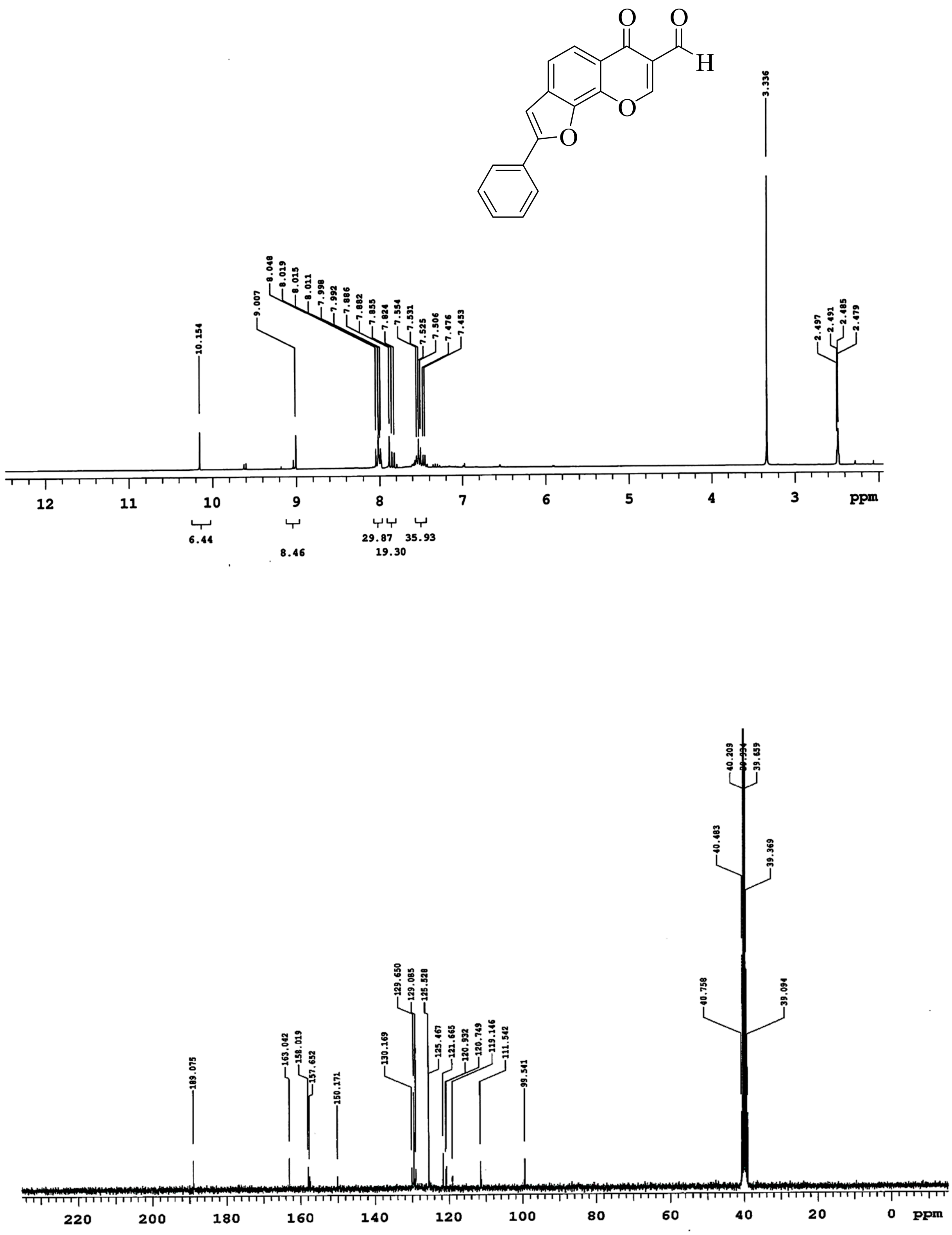


**Figure S2:** $^{1}H$- and $^{13}C$-NMR spectra of **2a** in DMSO-$d_6$ at 300 and 75 MHz, respectively.

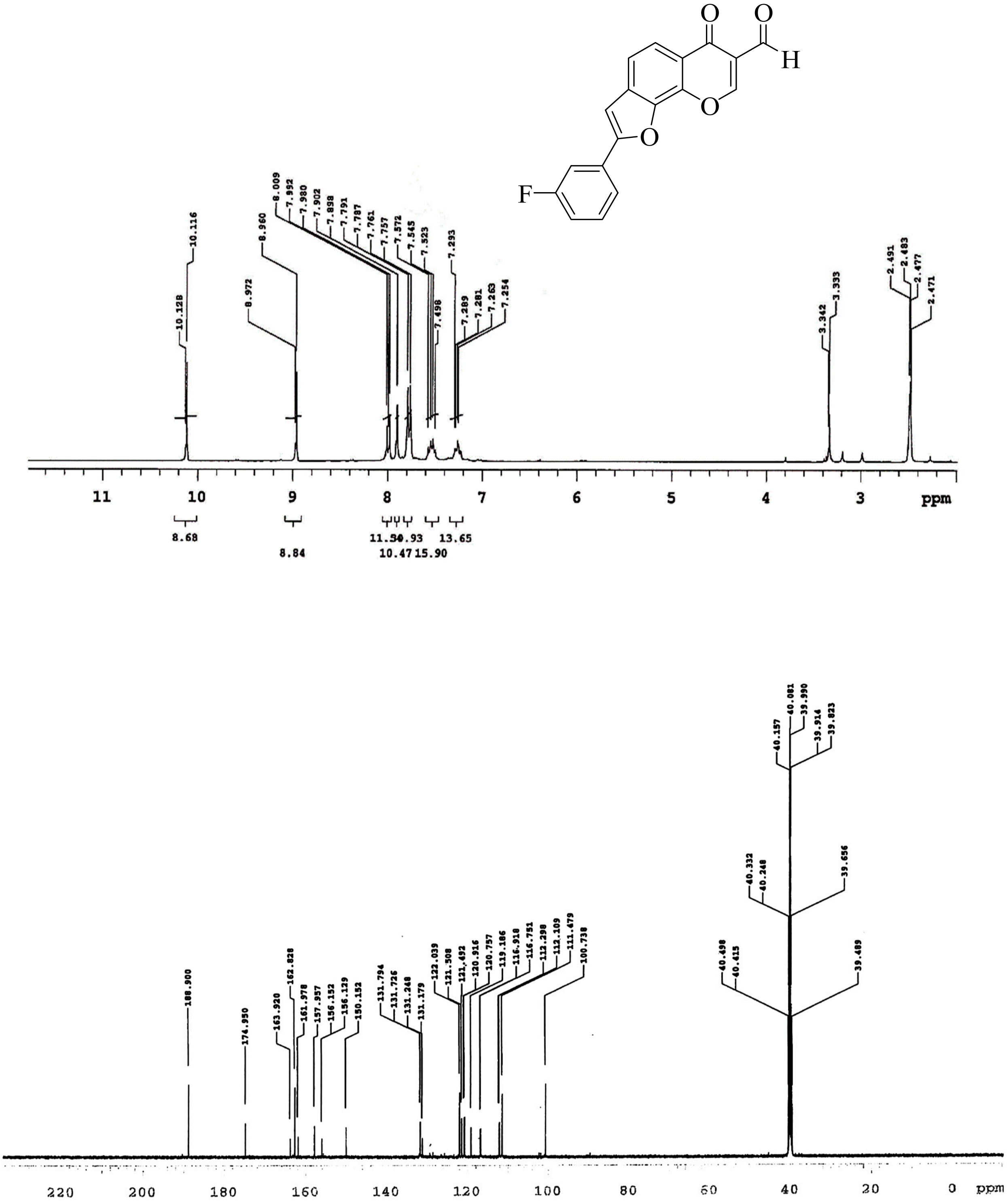


**Figure S3:** $^{1}$H- and $^{13}$C-NMR spectra of **2b** in DMSO-$d_6$ at 300 and 75 MHz, respectively.

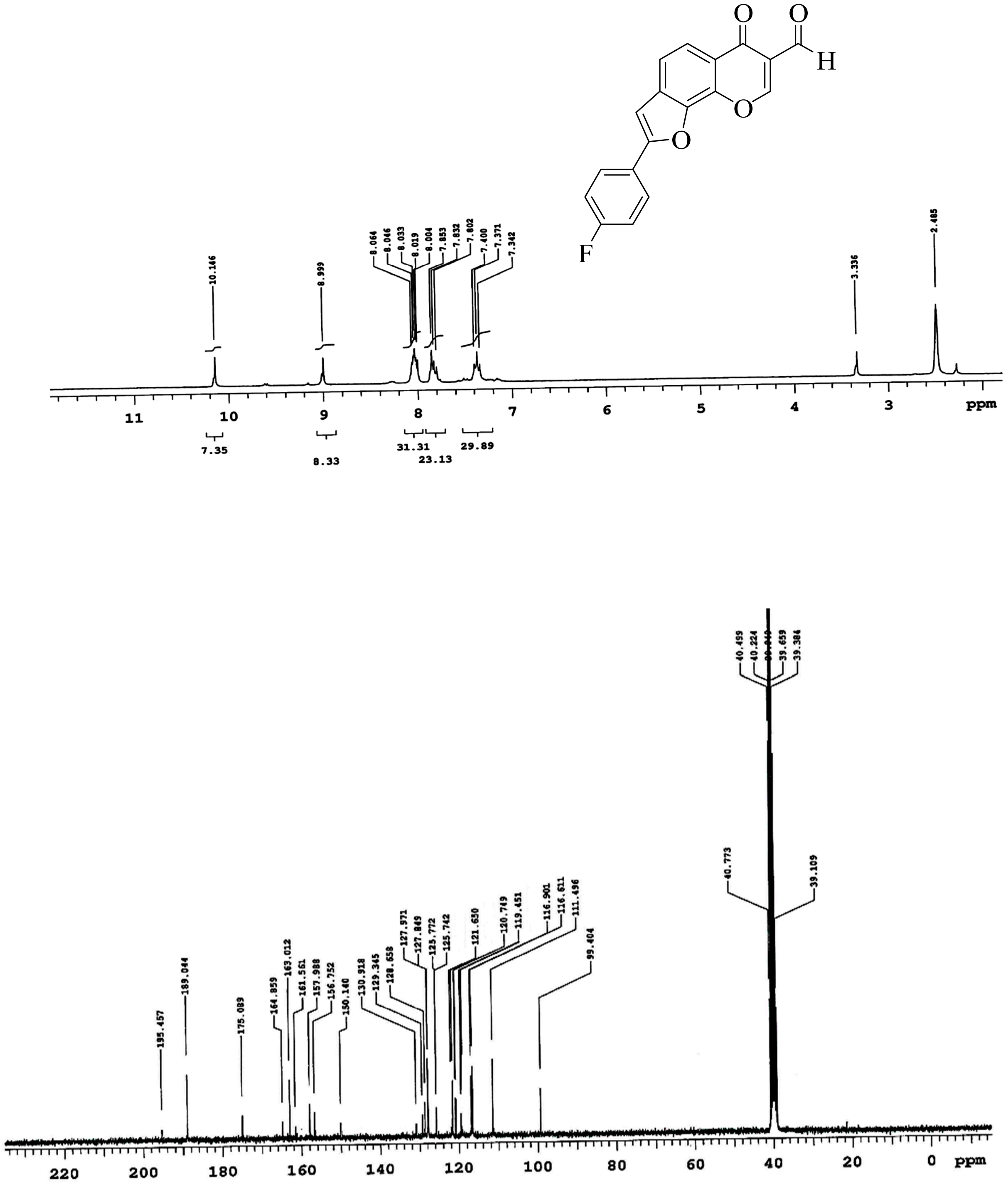


**Figure S4:** $^1$H- and $^{13}$C-NMR spectra of **2c** in DMSO-$d_6$ at 300 and 75 MHz, respectively.

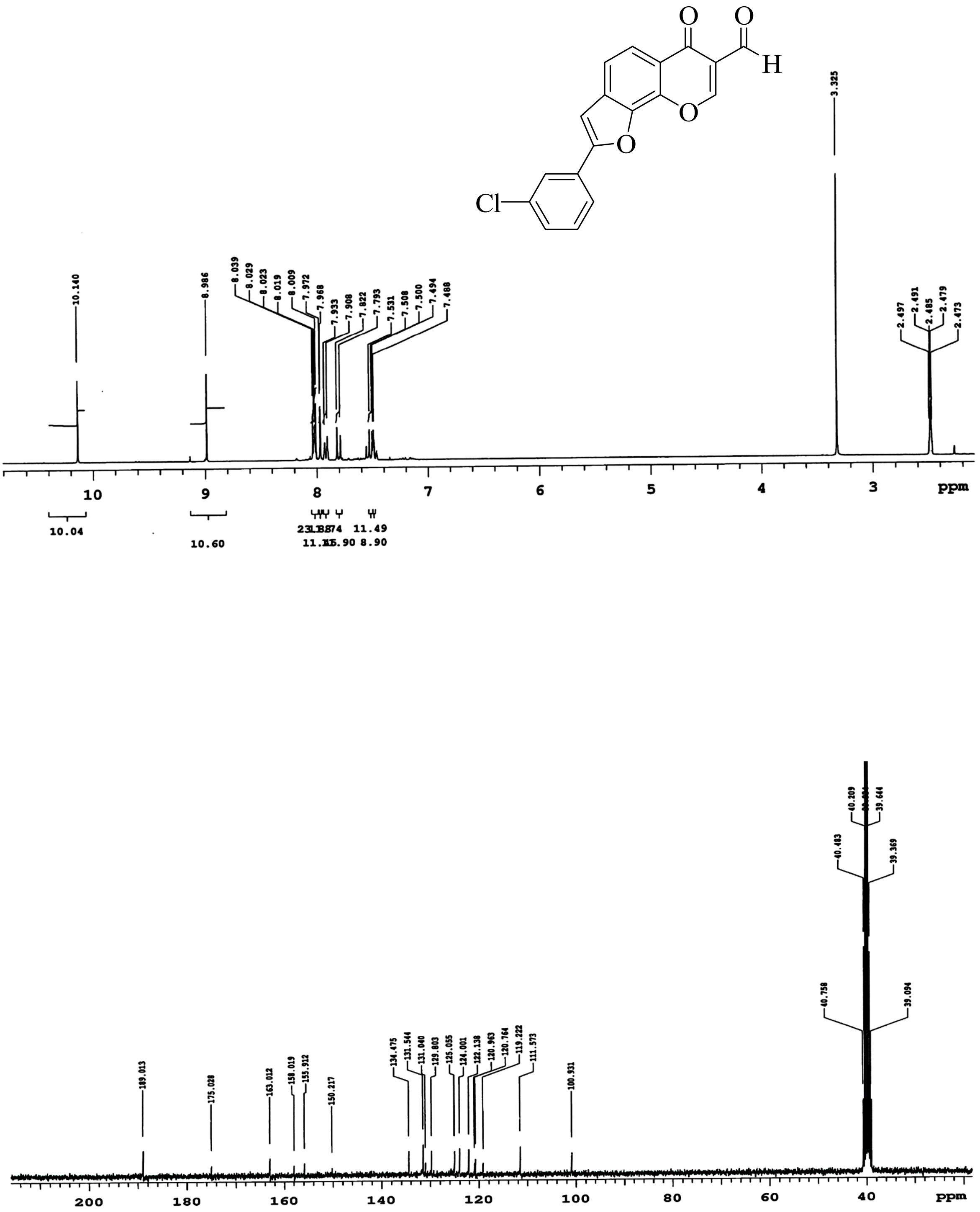


**Figure S5:** $^{1}$H- and $^{13}$C-NMR spectra of **2d** in DMSO-*d*$_6$ at 300 and 75 MHz, respectively.

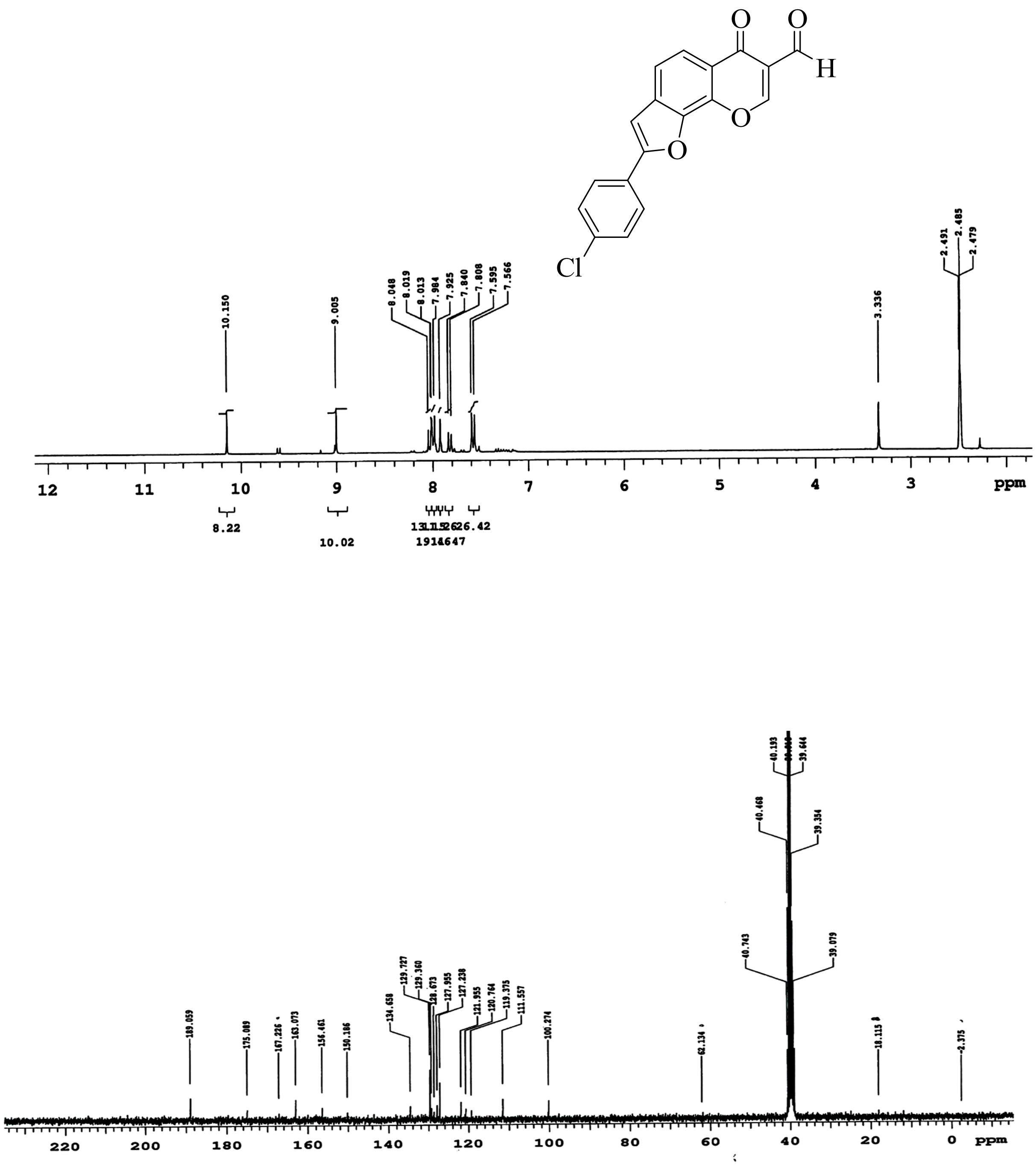


**Figure S6:** $^{1}$H- and $^{13}$C-NMR spectra of **2e** in DMSO-$d_6$ at 300 and 75 MHz, respectively.

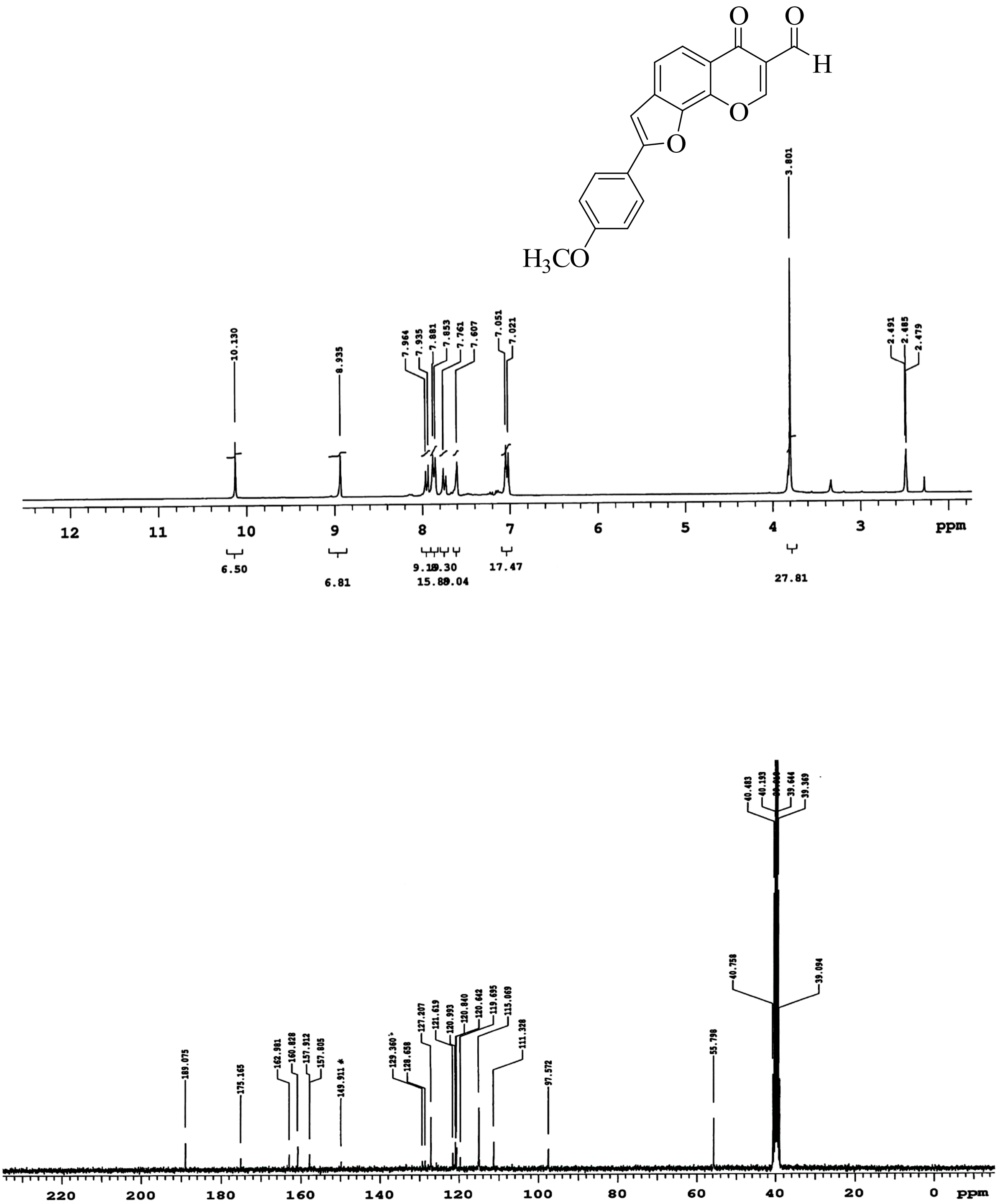


**Figure S7:** $^{1}$H- and $^{13}$C-NMR spectra of **2f** in DMSO-$d_6$ at 300 and 75 MHz, respectively.

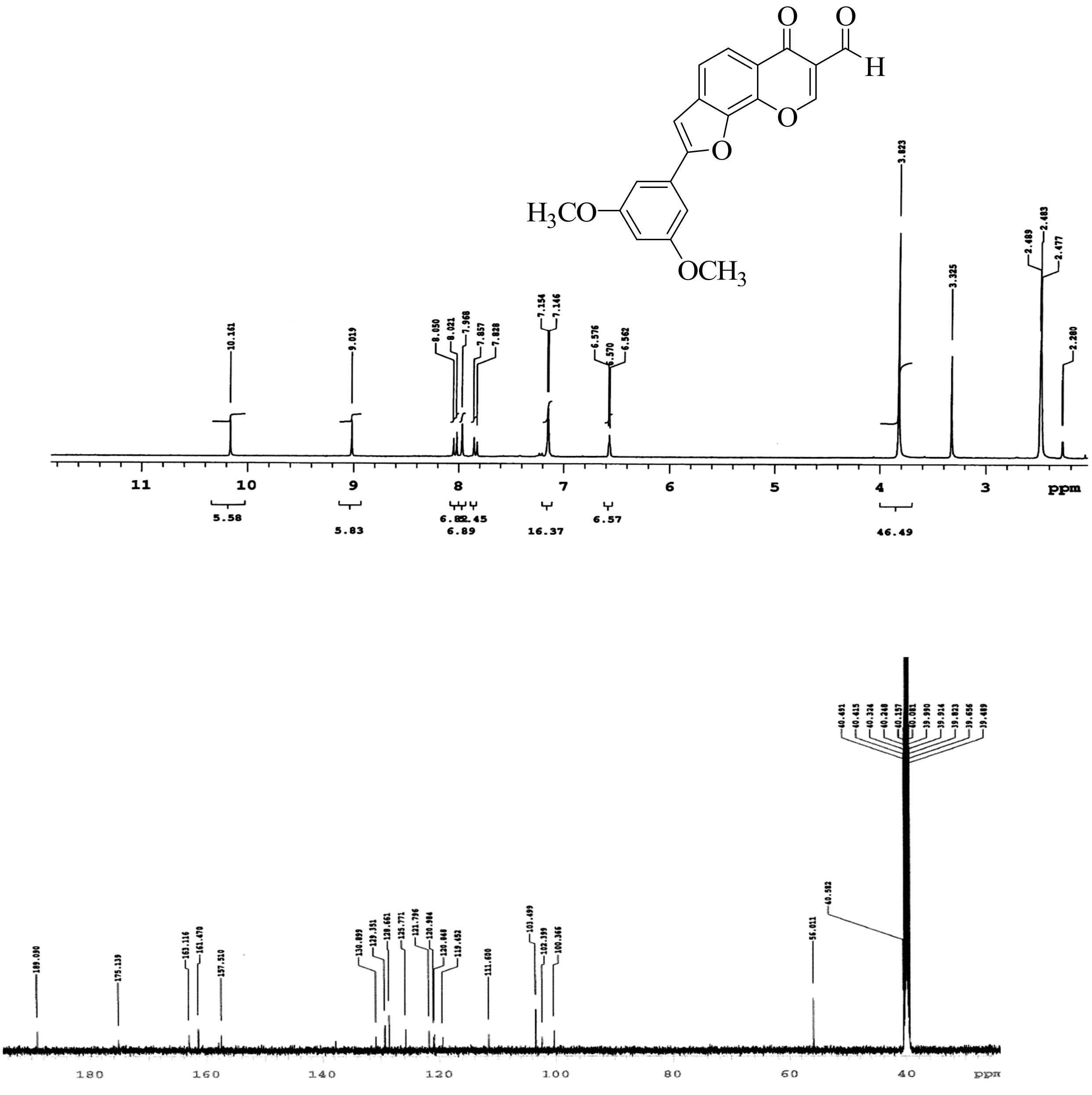


**Figure S8:** $^{1}$H- and $^{13}$C-NMR spectra of **2g** in DMSO-$d_6$ at 300 and 75 MHz, respectively.

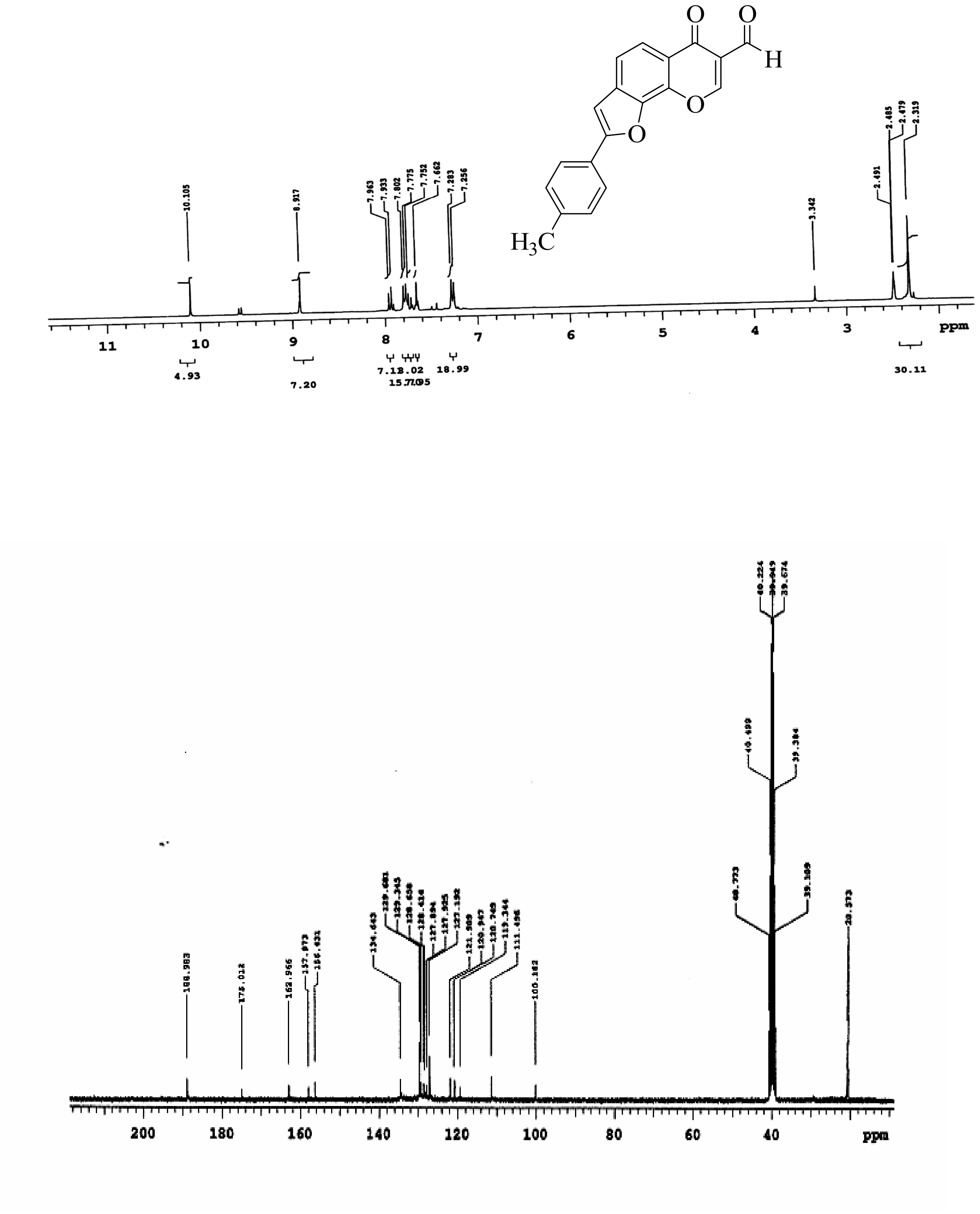


**Figure S9:** $^{1}H$- and $^{13}C$-NMR spectra of **2h** in DMSO-$d_6$ at 300 and 75 MHz, respectively.

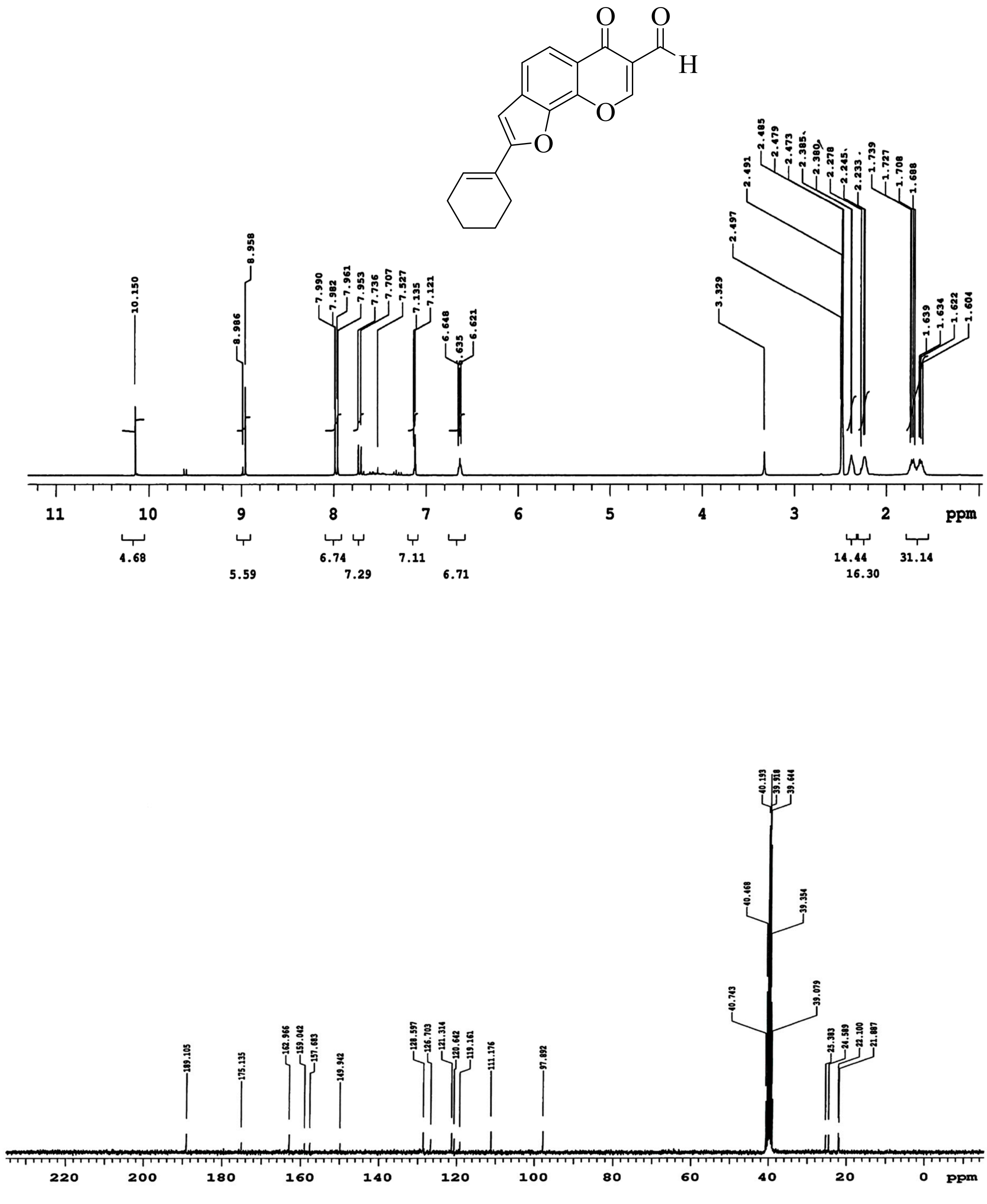


**Figure S10:** $^{1}$H- and $^{13}$C-NMR spectra of **2i** in DMSO-$d_6$ at 300 and 75 MHz, respectively

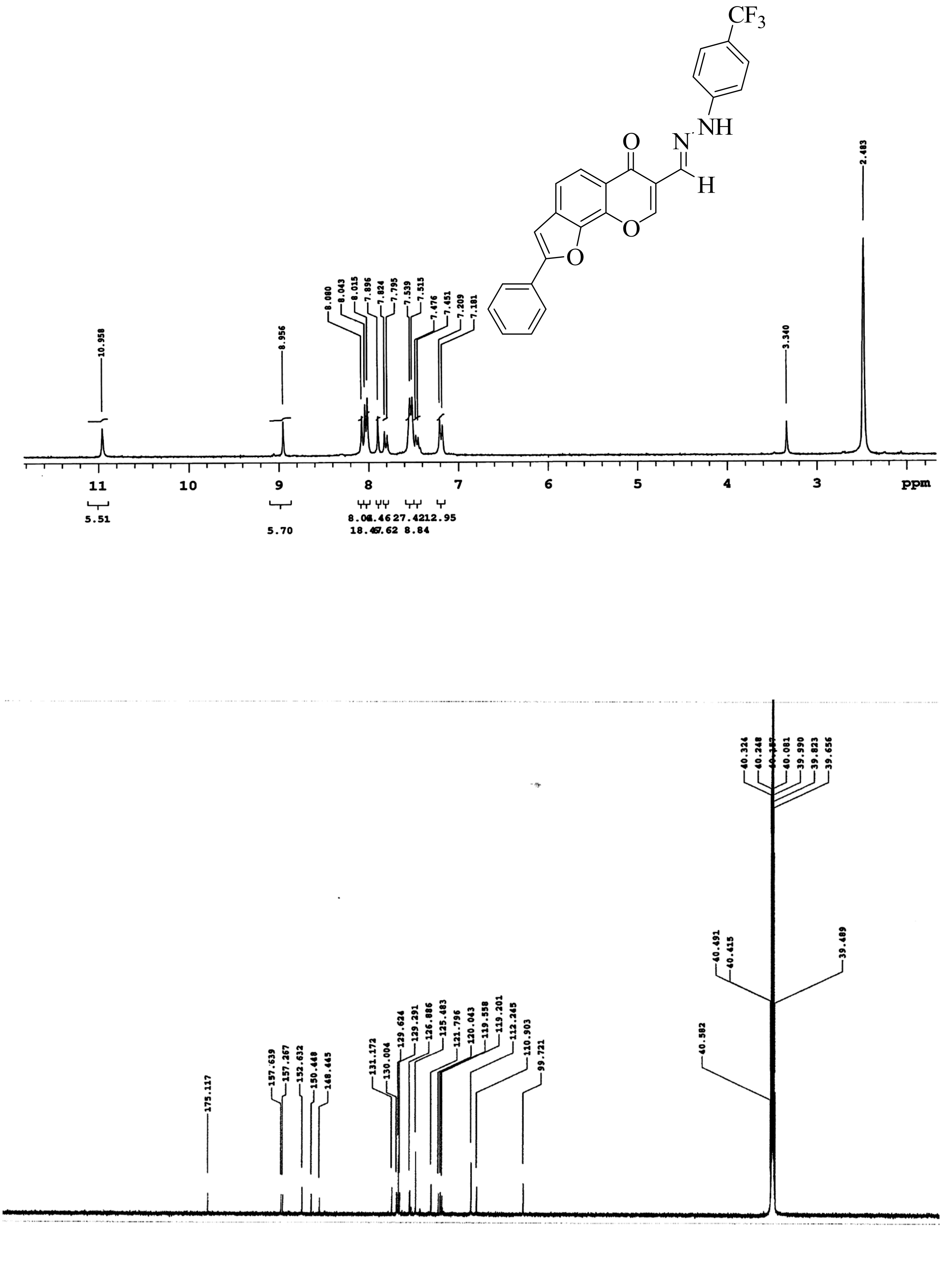


**Figure S11:** ¹H- and ¹³C-NMR spectra of **3a** in DMSO-$d_6$ at 300 and 75 MHz, respectively

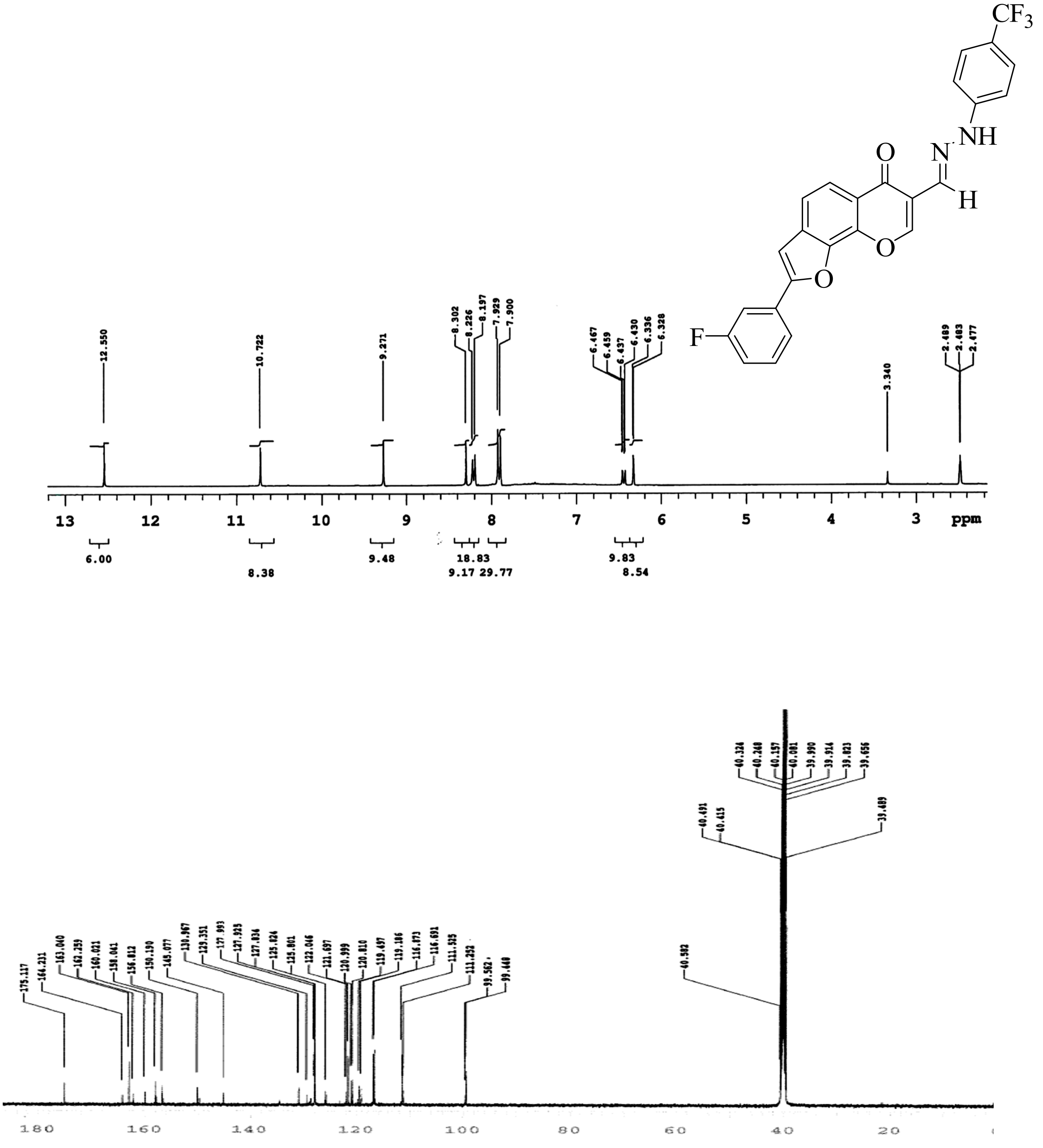


**Figure S12:** $^{1}$H- and $^{13}$C-NMR spectra of **3b** in DMSO-$d_6$ at 300 and 75 MHz, respectively.

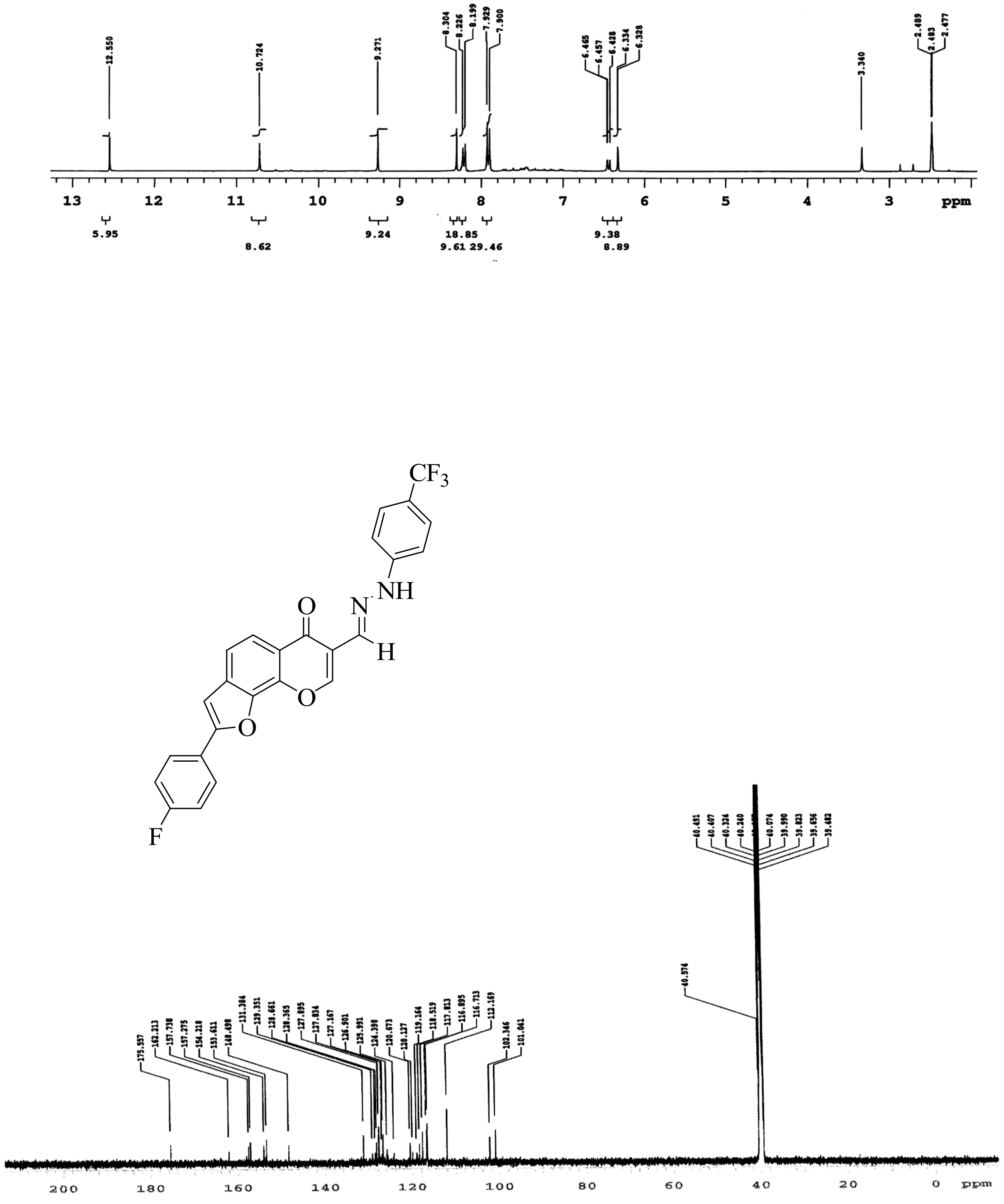


**Figure S13:** ${}^{1}$H- and ${}^{13}$C-NMR spectra of **3c** in DMSO-*d*6 at 300 and 75 MHz, respectively.

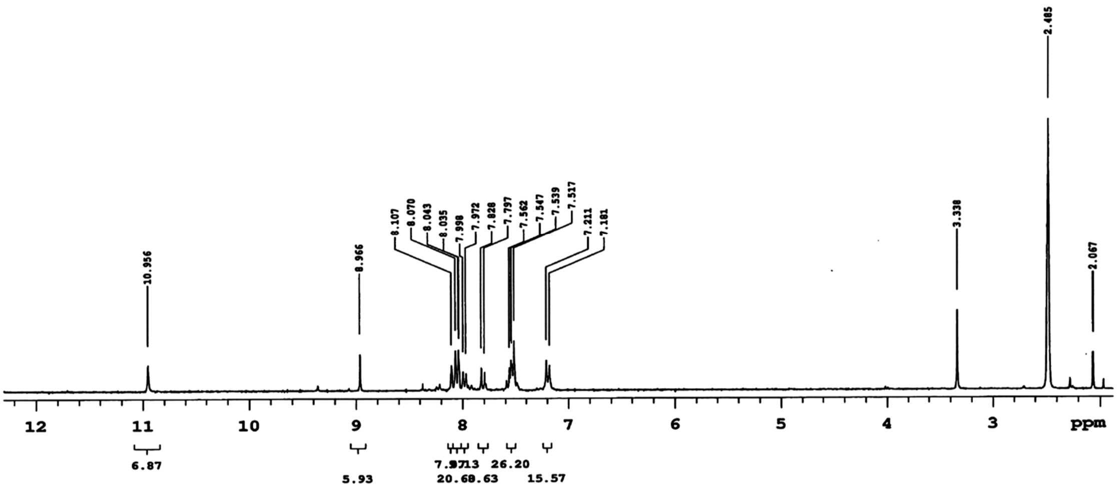


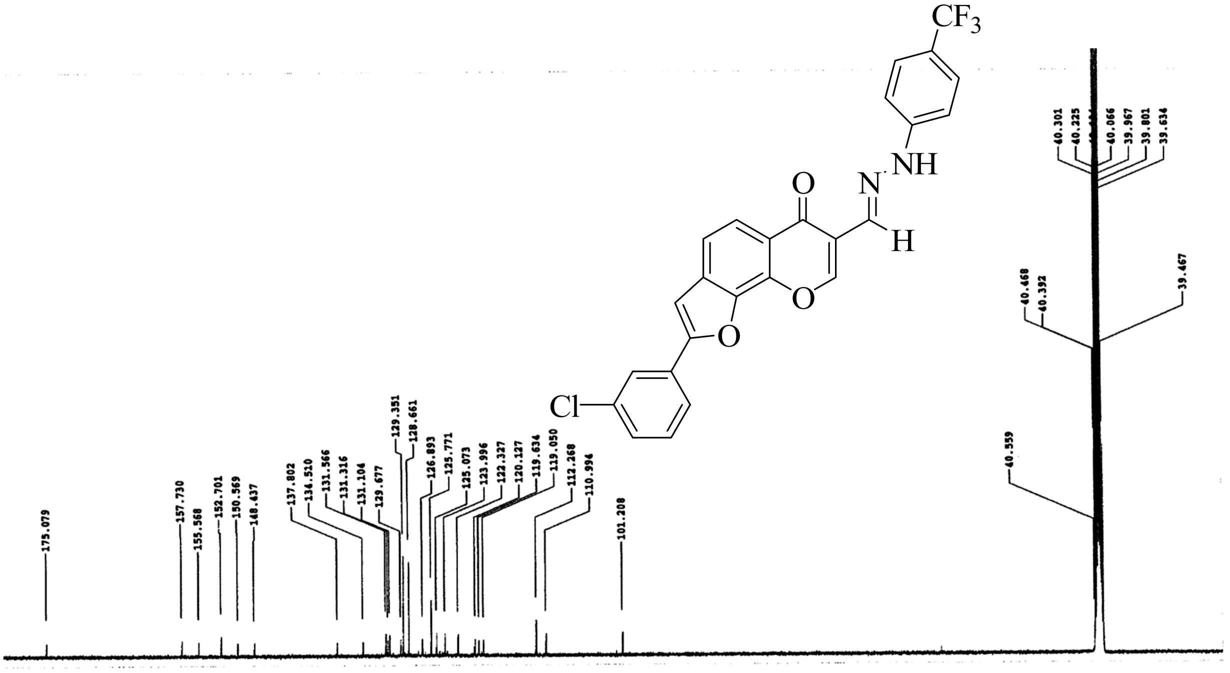


**Figure S14:** $^{1}H$- and $^{13}C$-NMR spectra of **3d** in DMSO-$d_6$ at 300 and 75 MHz, respectively

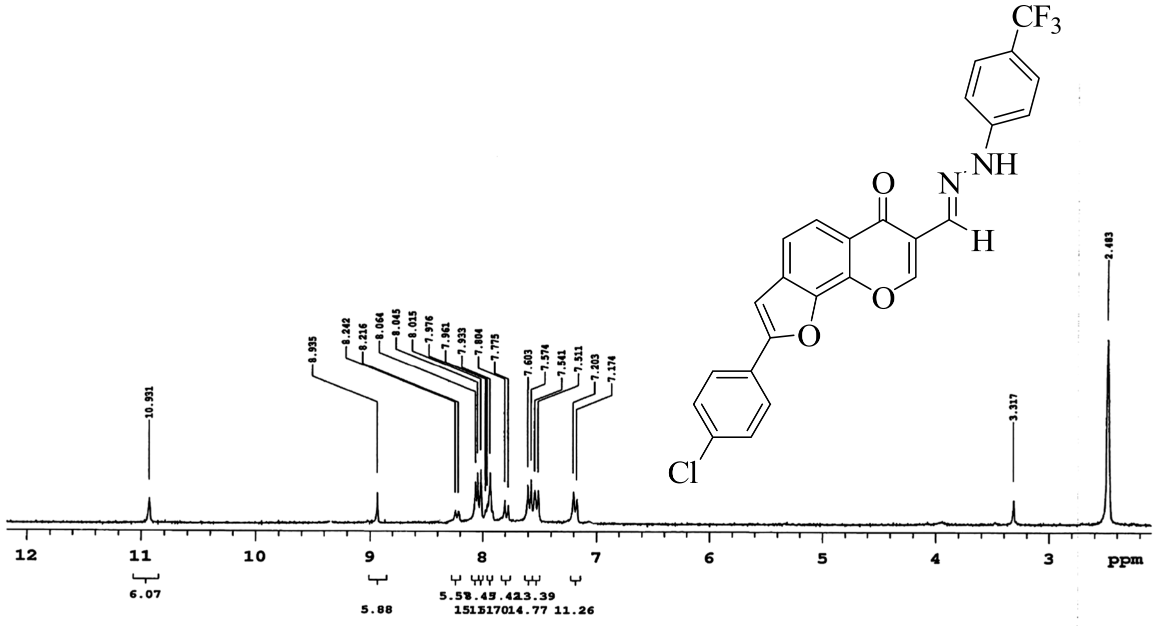

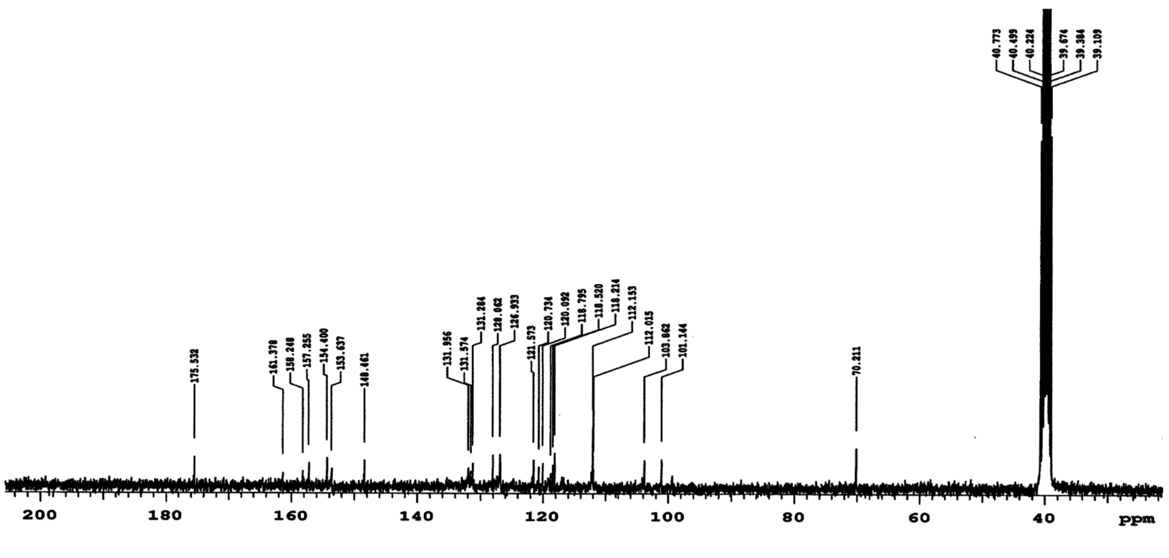

**Figure S16:** $^{1}$H- and $^{13}$C-NMR spectra of **3e** in DMSO-*d*6 at 300 and 75 MHz, respective

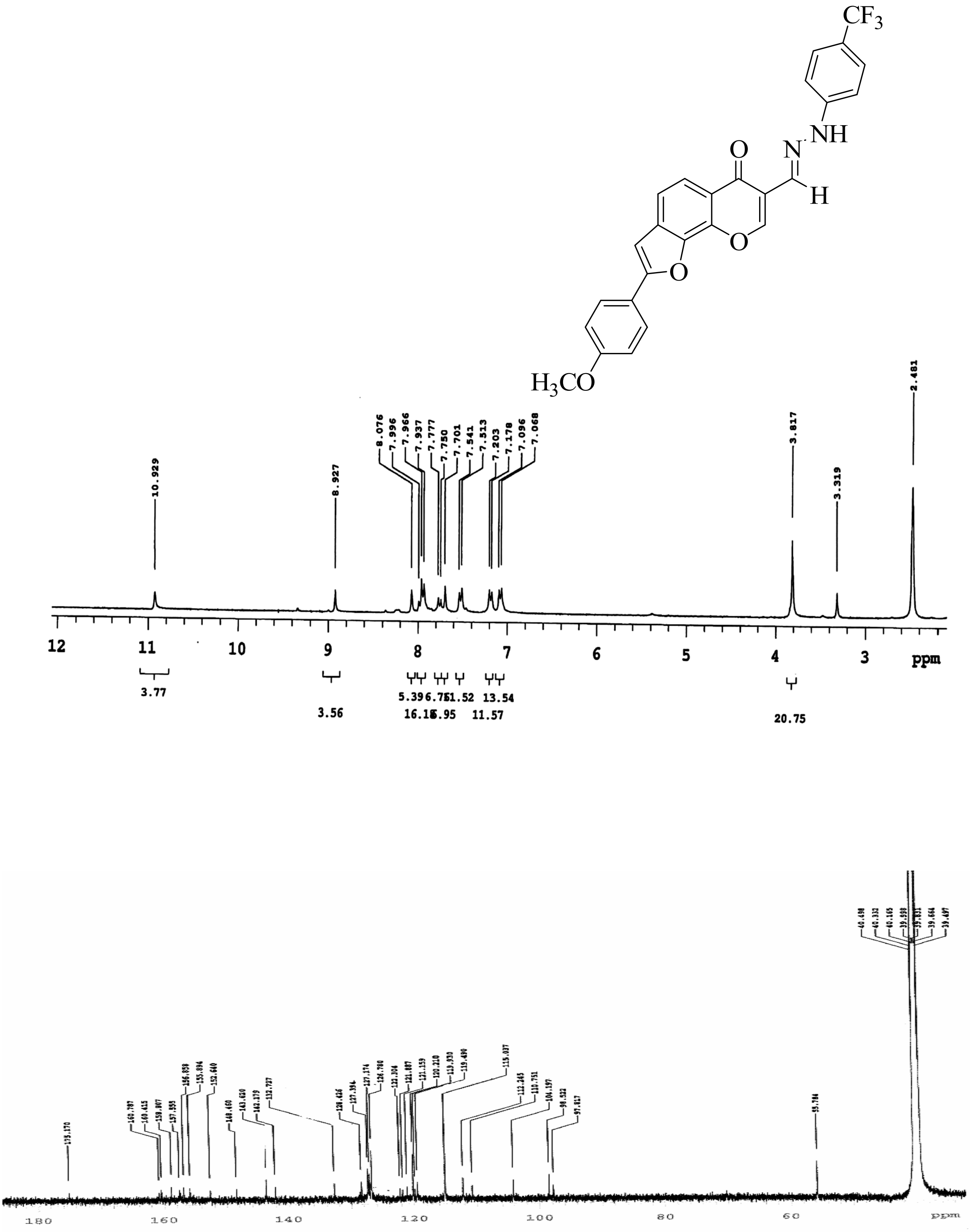


**Figure S1.17:** $^{1}$H- and $^{13}$C-NMR spectra of **3f** in DMSO-$d_6$ at 300 and 75 MHz, respectively.

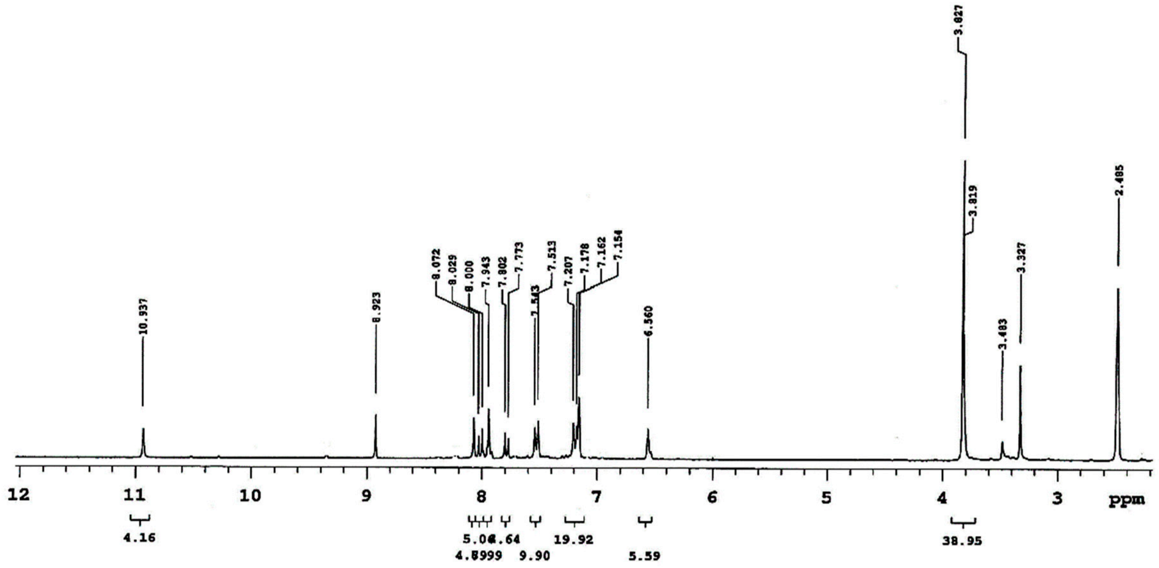

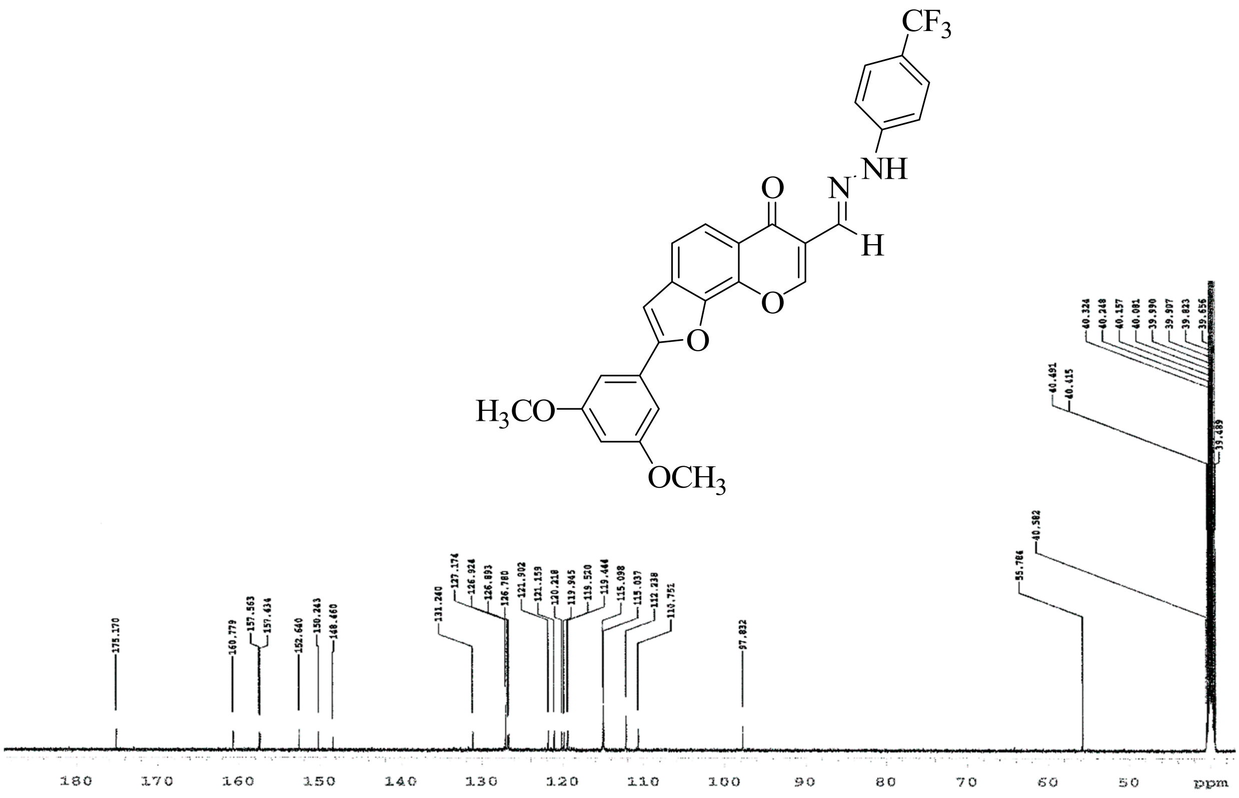


**Figure S18:** $^{1}$H- and $^{13}$C-NMR spectra of **3g** in DMSO-$d_6$ at 300 and 75 MHz, respectively.

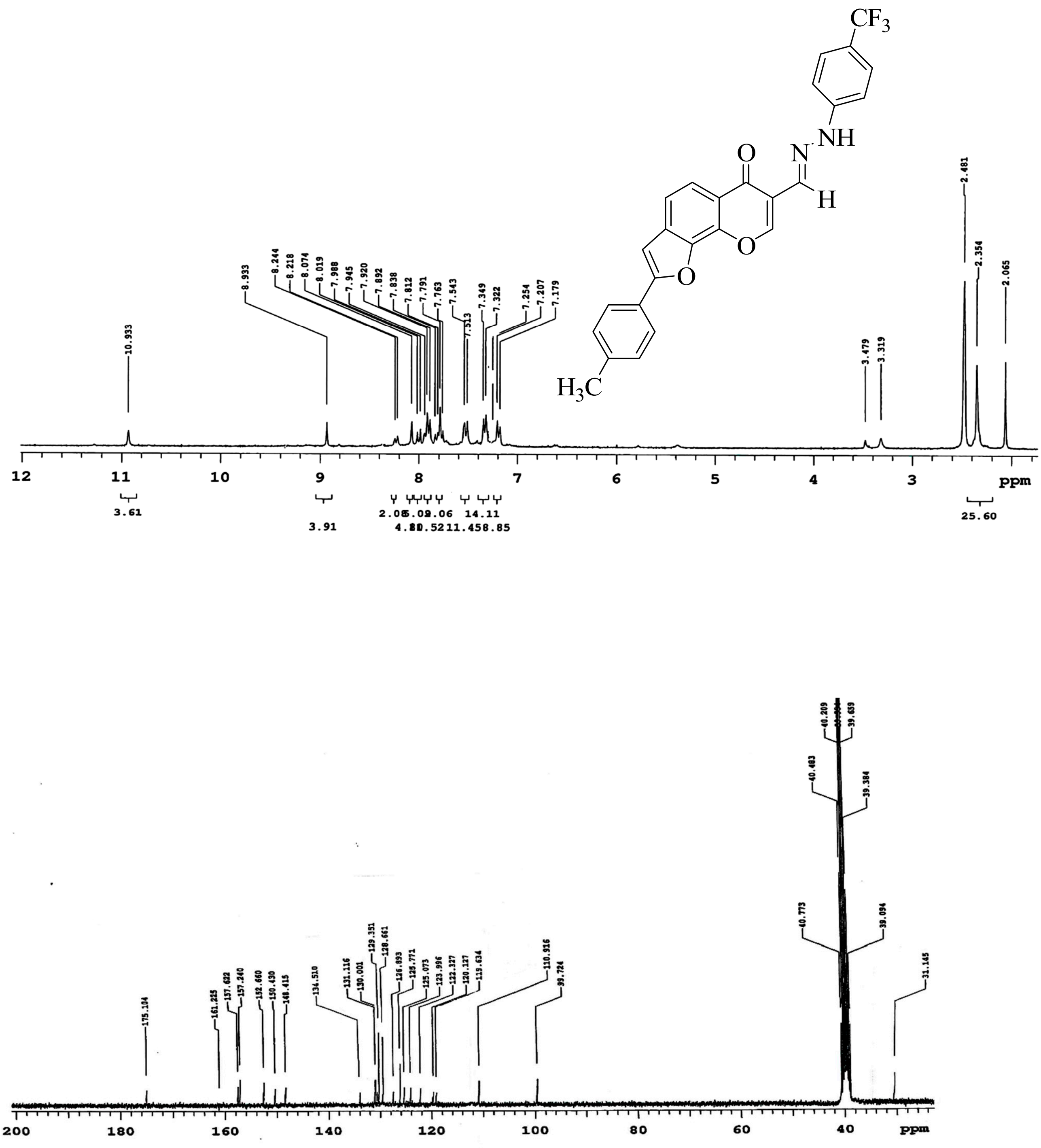


**Figure S1.19:** $^{1}$H- and $^{13}$C-NMR spectra of **3h** in DMSO-$d_6$ at 300 and 75 MHz, respectively.

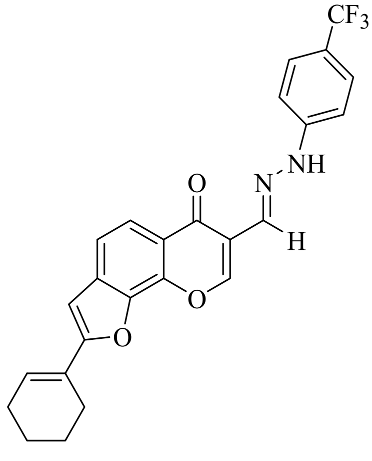


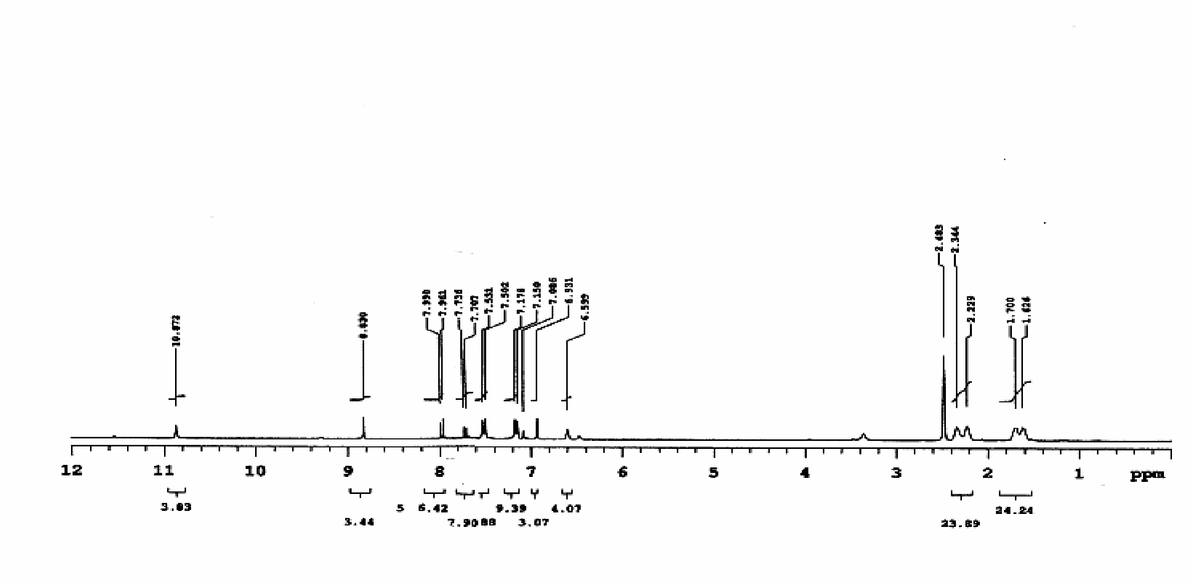


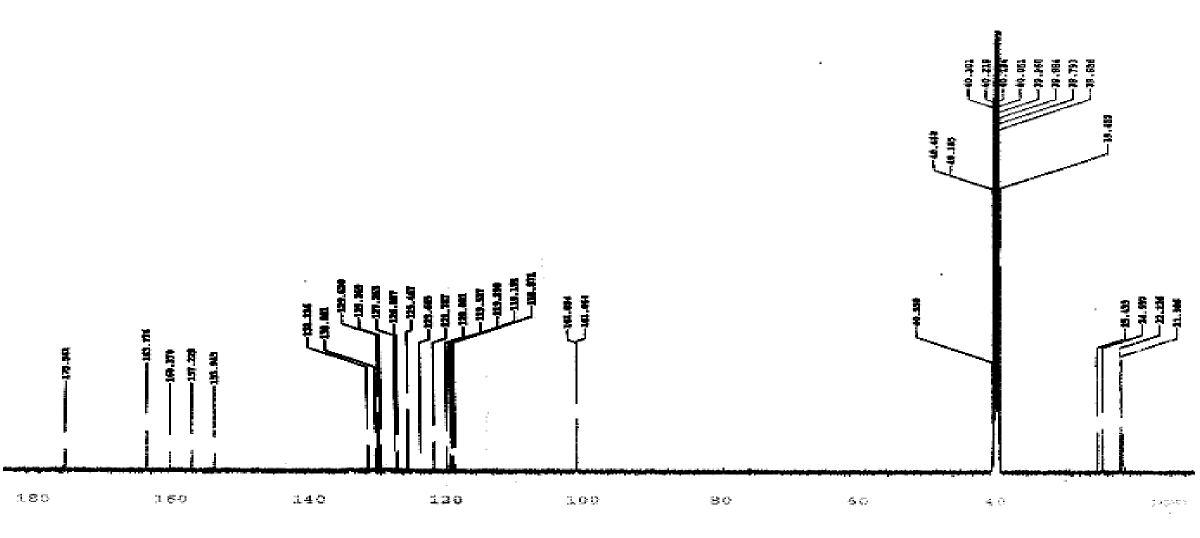


**Figure S1.19:** $^{1}H$- and $^{13}C$-NMR spectra of **3i** in DMSO-$d_6$ at 300 and 75 MHz, respectively.

**Figure S2: Lineweaver-Burk and Dixon plots of 3b and 3e against AChE activity.**

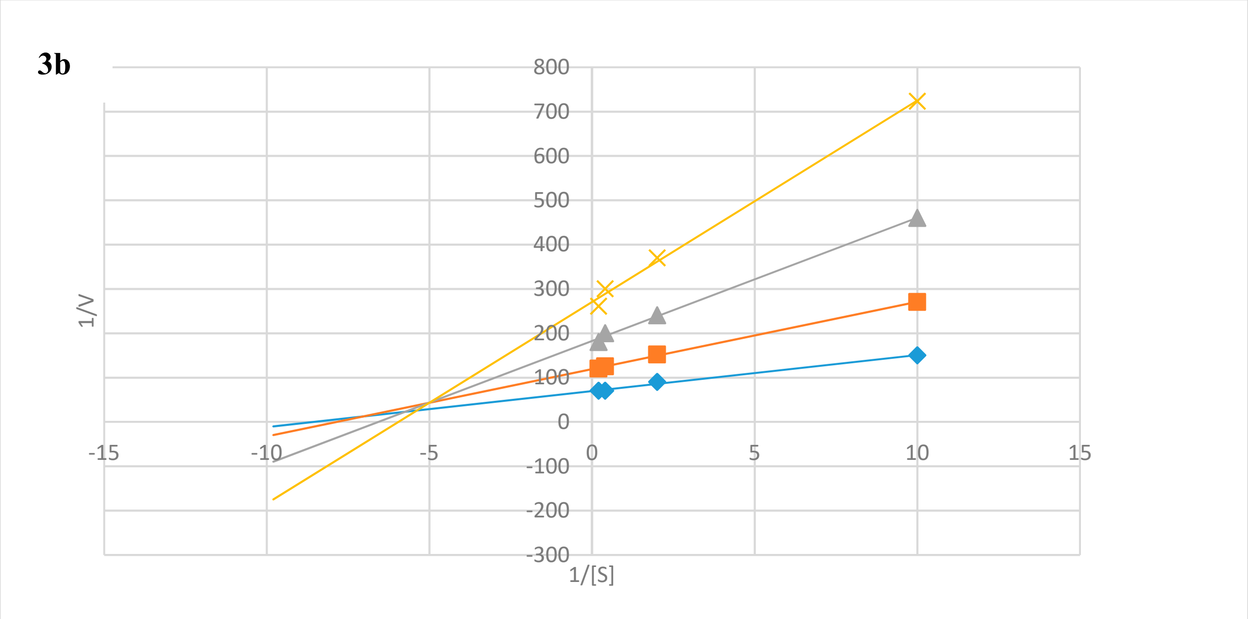


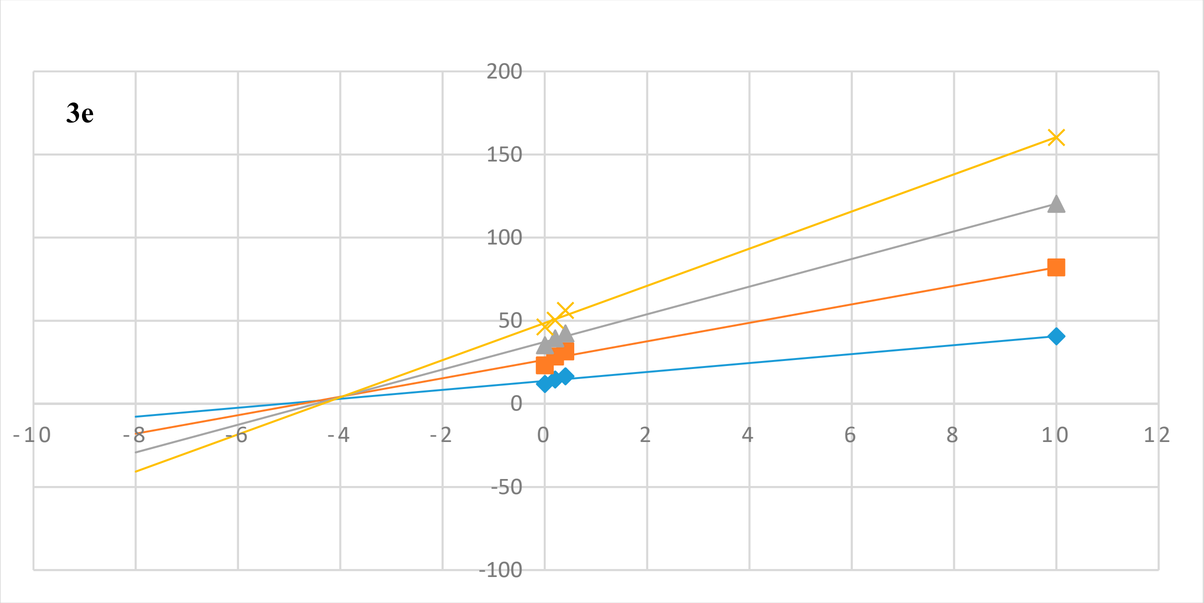


**Figure S2.1:** Lineweaver–Burk plots for inhibition of AChE by **3b** and **3e**. Blue symbols and fitted straight lines represent enzyme activity in the absence of inhibitor, while orange (2.5 µM), grey (3.5 µM) and yellow (5 µM) represent various concentrations of inhibitor.

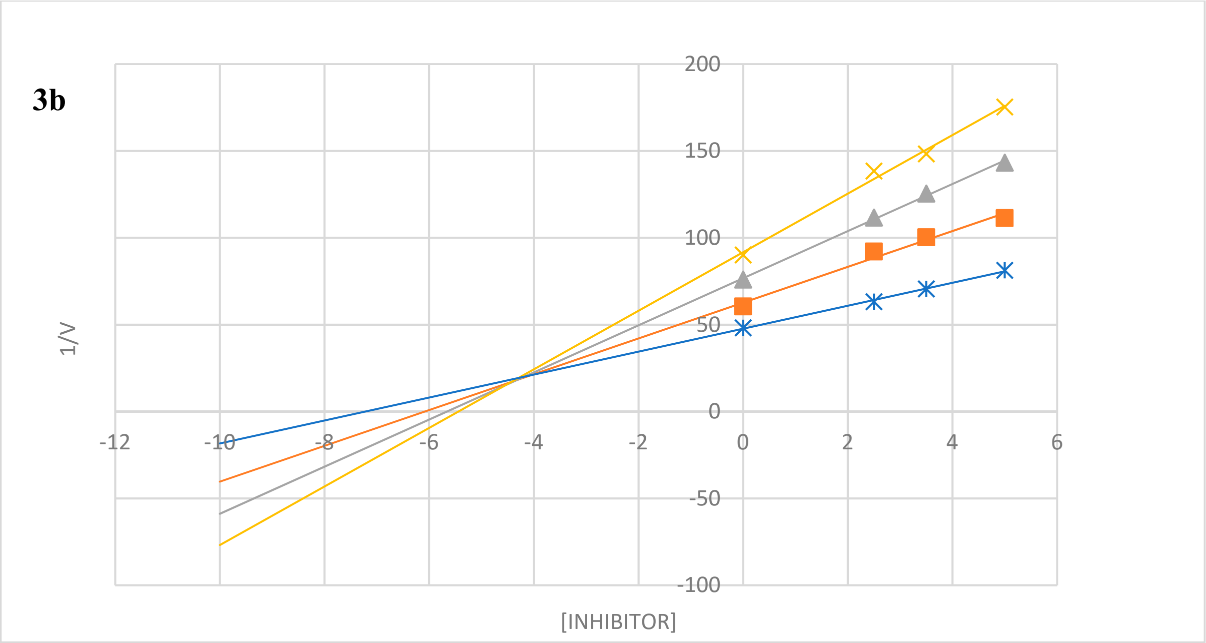


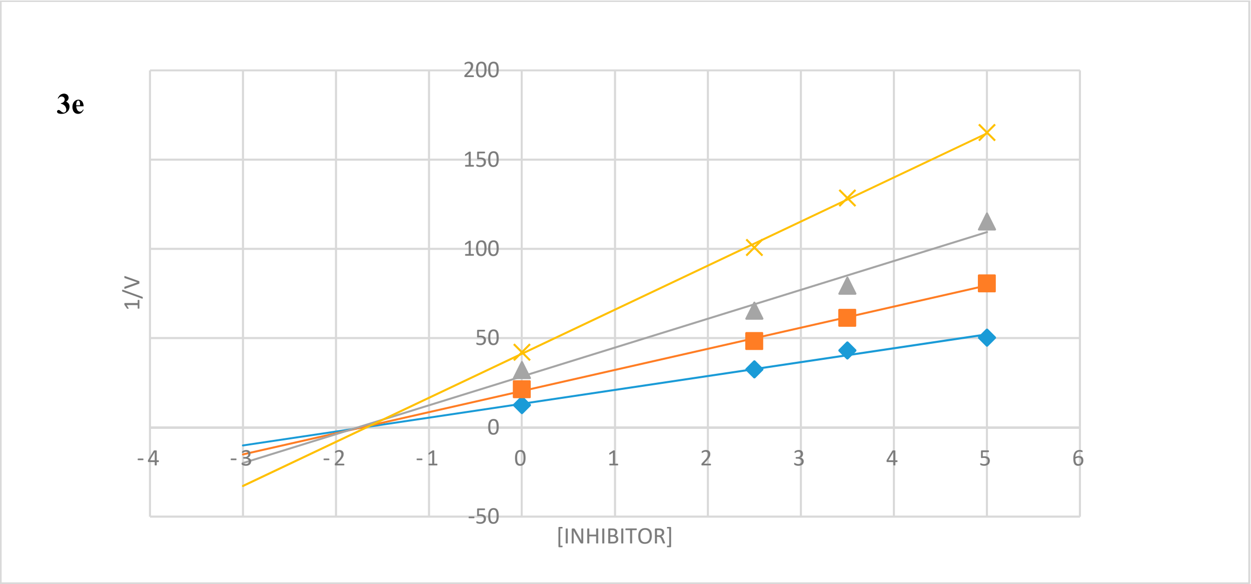


**Figure S2.2:** Dixon plots for inhibition of AChE by **3b** and **3e**. Blue symbols and fitted straight lines represent enzyme activity in the presence of 5mM substrate, while orange (2.5 mM), grey (0.5 mM) and yellow (0.1 mM) represent various concentrations of substrate.

**Figure S3: Lineweaver-Burk and Dixon plots of 3b and 3e against BChE activity**

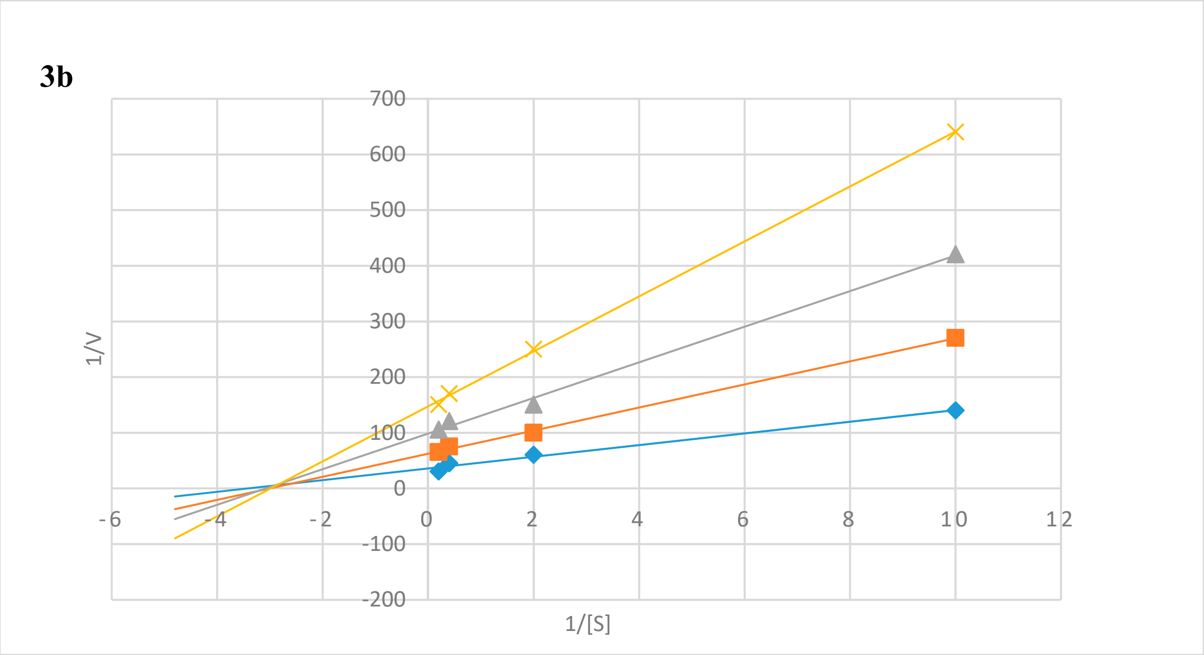


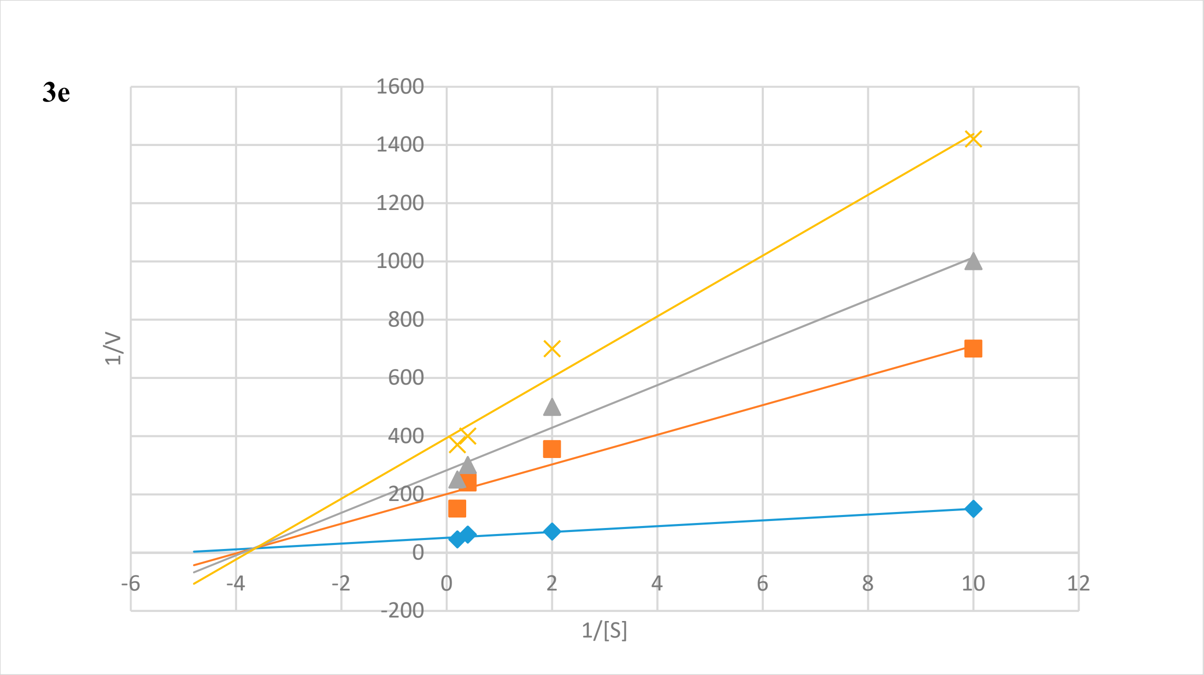


**Figure S3.1:** Lineweaver–Burk plots for inhibition of BChE by **3b** and **3e**. Blue symbols and fitted straight lines represent enzyme activity in the absence of inhibitor, while orange (2.5 µM), grey (3.5 µM) and yellow (5 µM) represent various concentrations of inhibitor.

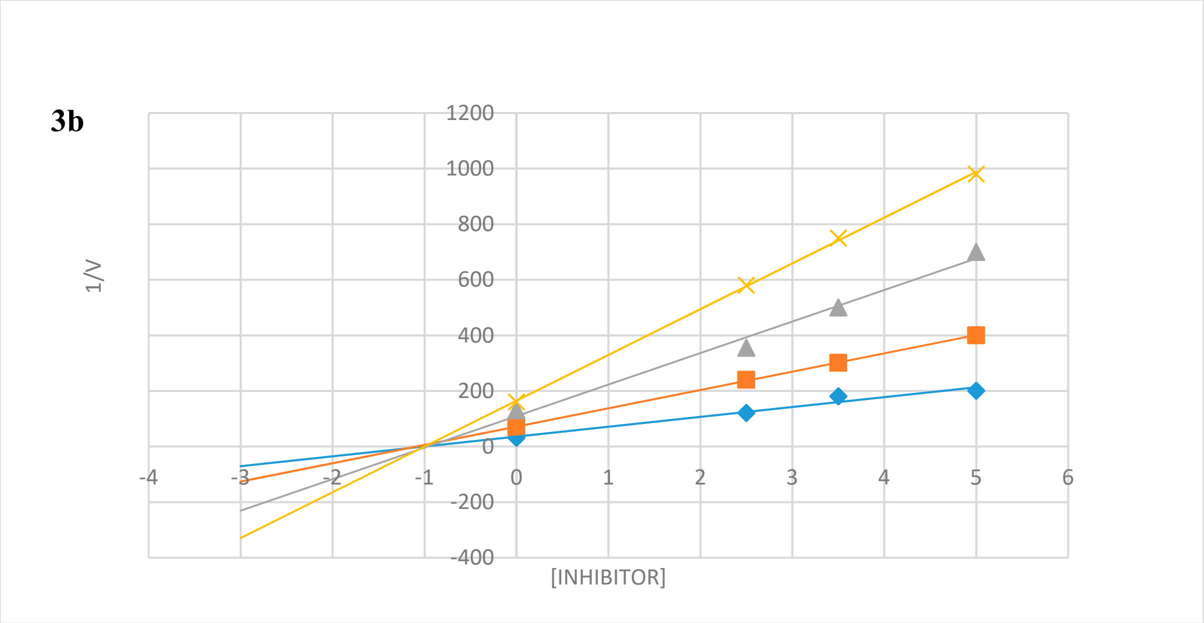


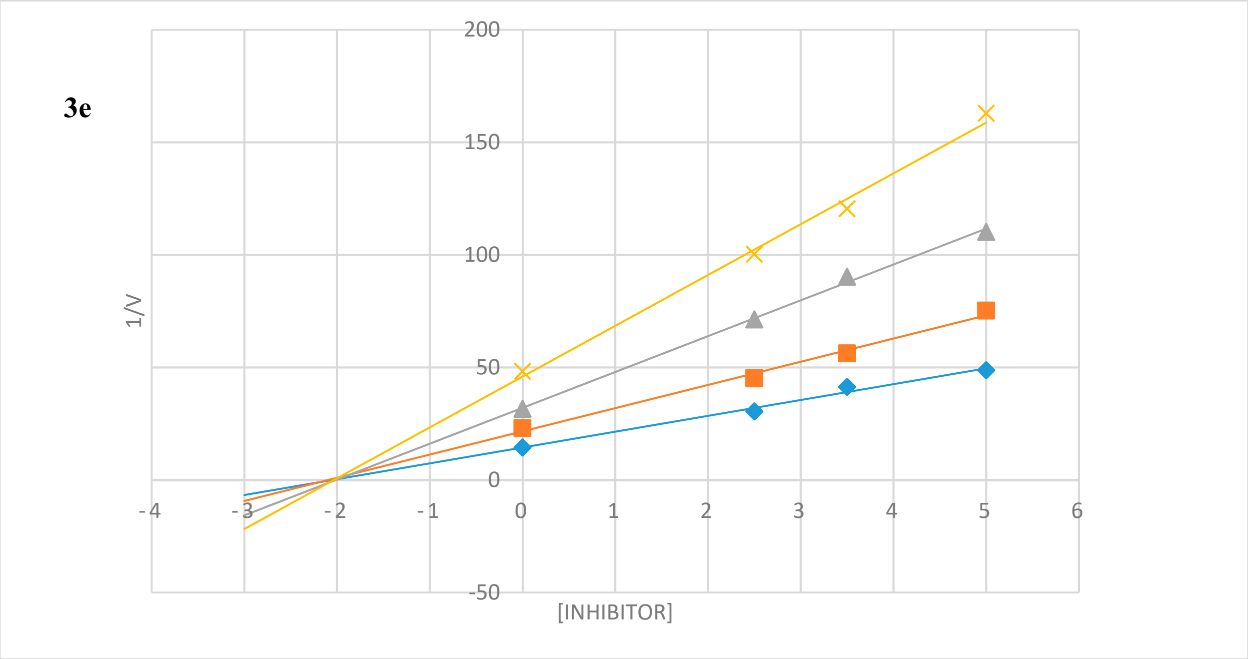


**Figure S3.2:** Dixon plots for inhibition of BChE by **3b** and **3e**. Blue symbols and fitted straight lines represent enzyme activity in the presence of 5mM substrate, while orange (2.5 mM), grey (0.5 mM) and yellow (0.1 mM) represent various concentrations of substrate.

**Figure S4: Lineweaver-Burk and Dixon plots of 3b and 3e against BACE-1 activity.**

**3b**

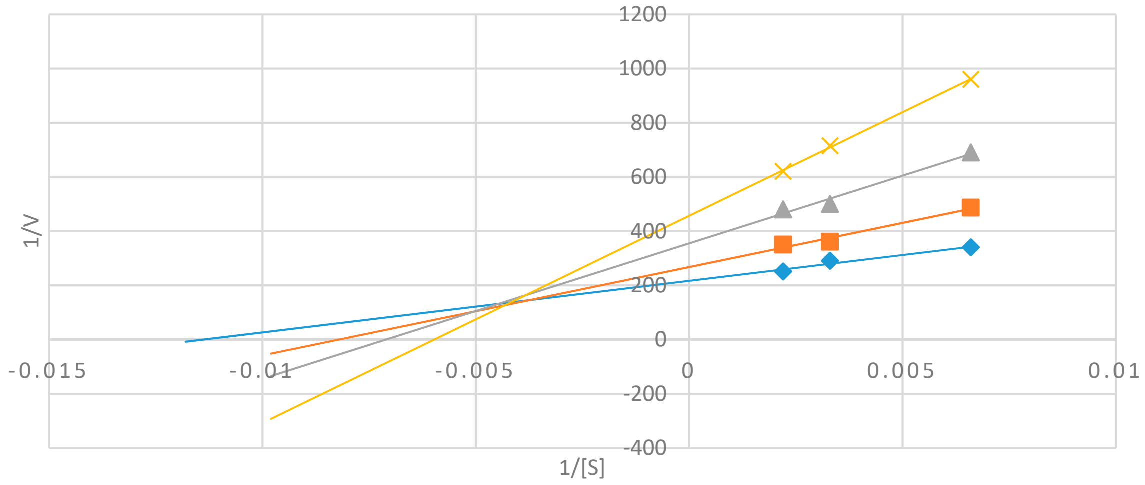


**3e**

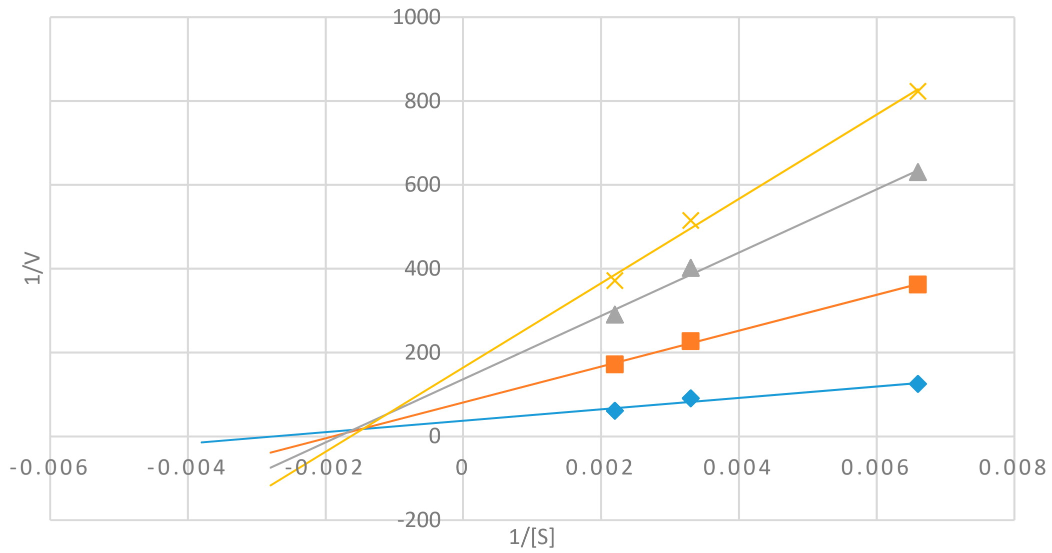


**Figure S4.1:** Lineweaver–Burk plots for inhibition of BACE-1 by **3b** and **3e**. Blue symbols and fitted straight lines represent enzyme activity in the absence of inhibitor, while orange (4 μM), grey (8 μM) and yellow (16 μM) represent various concentrations of inhibitor.

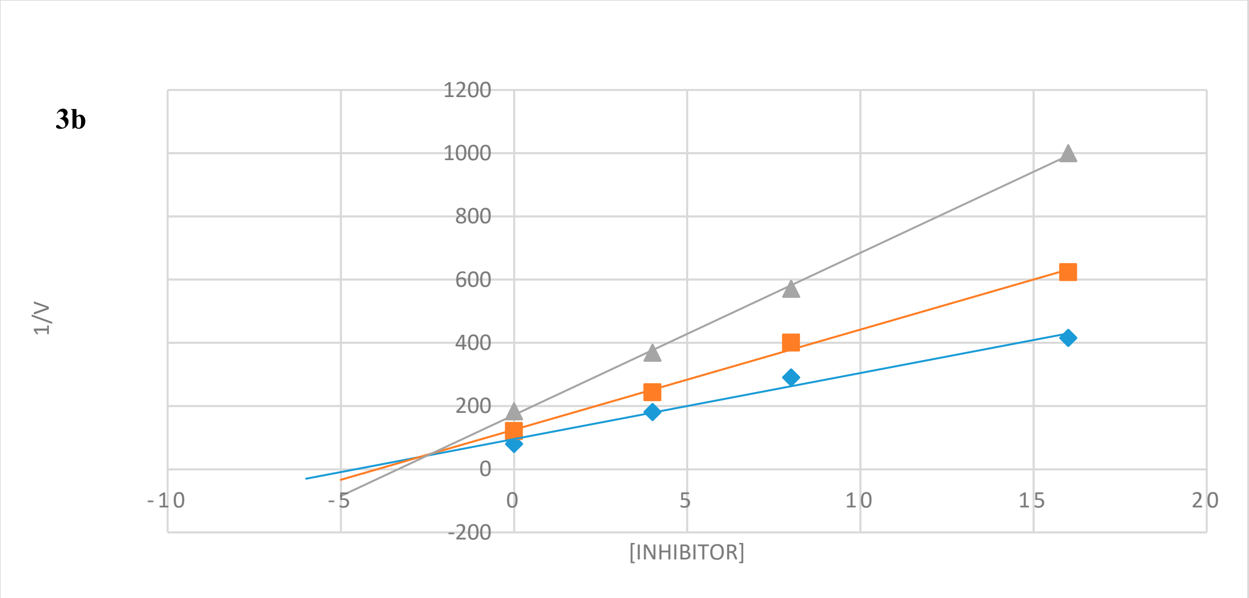


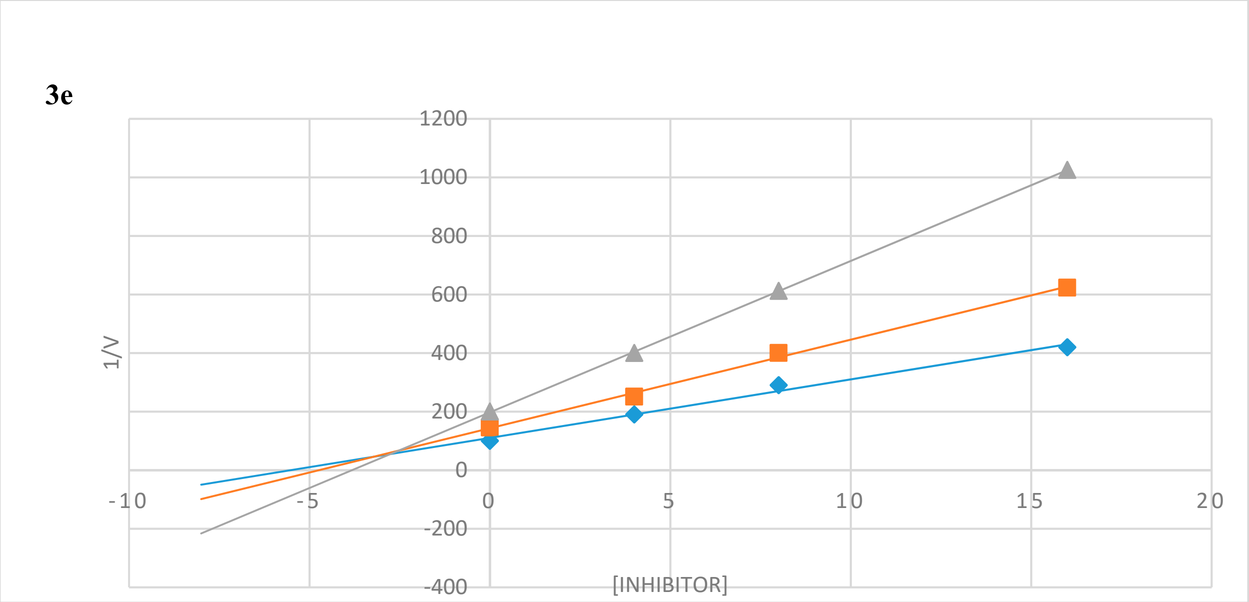


**Figure S4.2:** Dixon plots for inhibition of BACE-1 by **3b** and **3e**. Blue symbols and fitted straight lines represent enzyme activity in the presence of 450 nM substrate and orange and grey, 300 nM and 150 nM substrate, respectively.

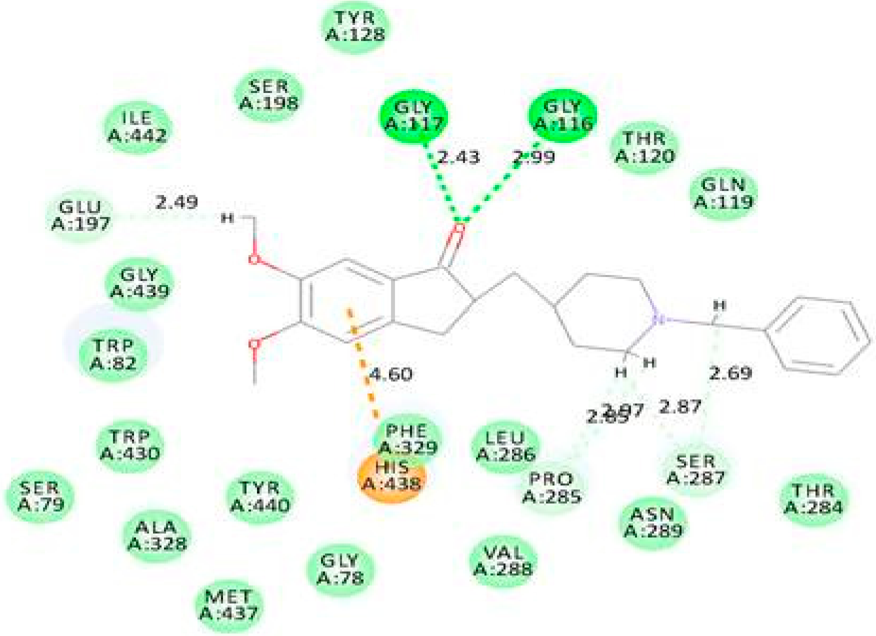


**Figure S5:** Docking pose of donepezil showing interaction with BChE protein residues. . Bright green represents conventional hydrogen bonds, very light green carbon hydrogen bonds and orange- pi-anion or cation interactions.

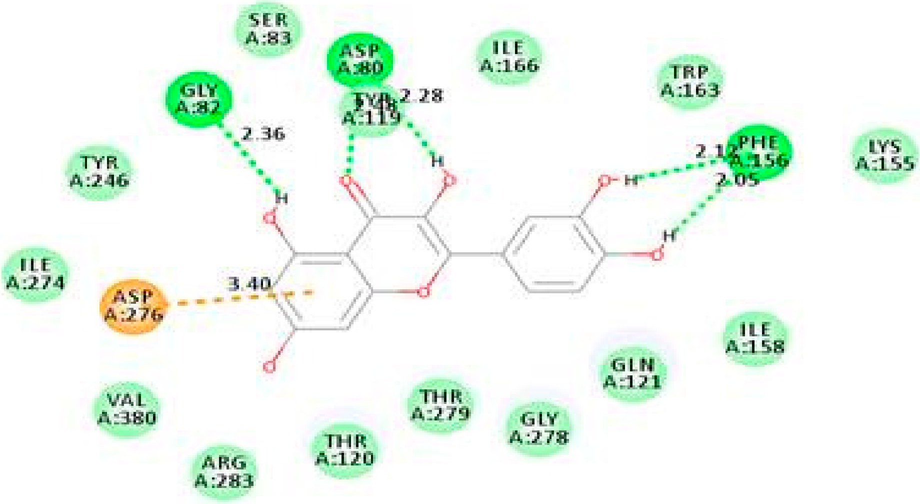


**Figure S6:** Docking pose of quercetin showing interaction with BACE-1 protein residues. Bright green represents conventional hydrogen bonds very light green carbon hydrogen bonds and orange- pi-anion or cation interactions.

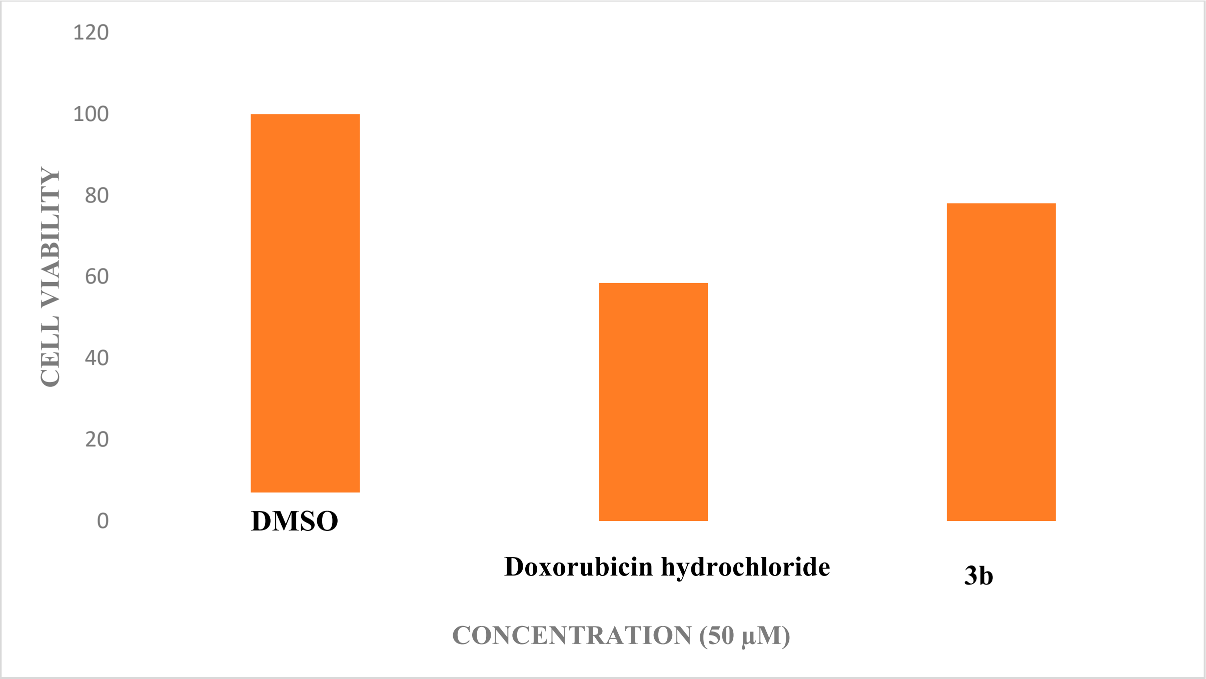


(a)

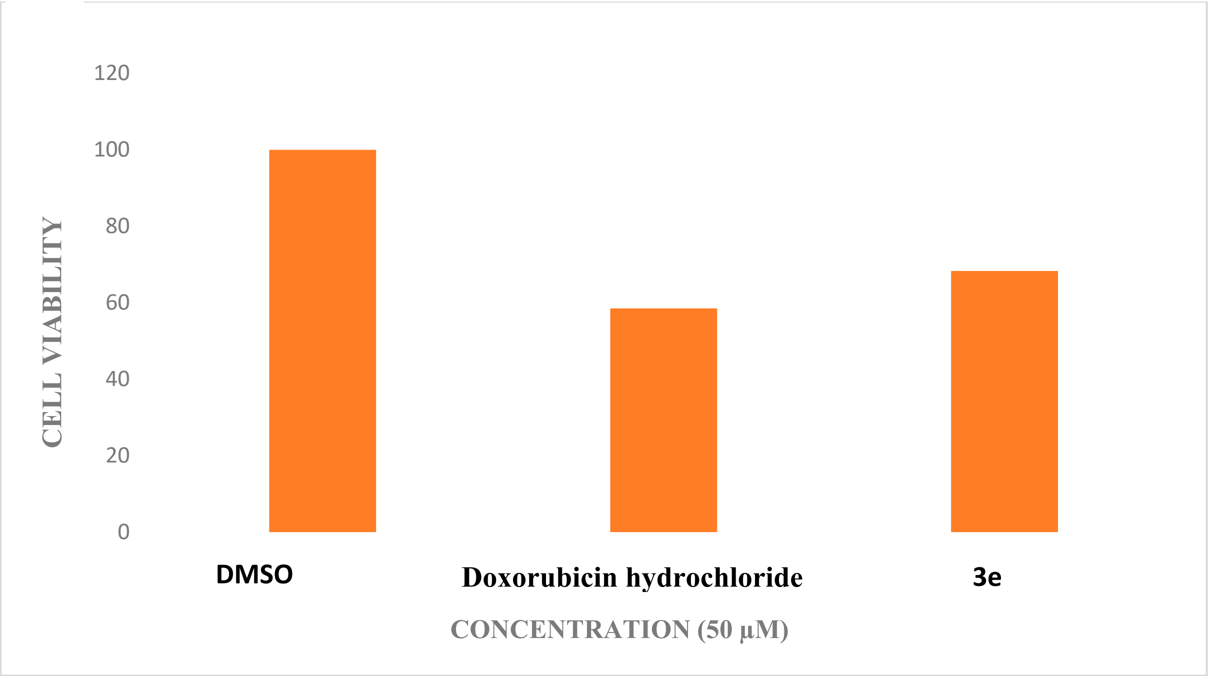


(b)

**Figure S7:** Evaluation of toxicity of **3b** (a) and **3e** (b) in Hek293-T cells.
